# Comparative Genomic Analysis Reveals Gene Content Diversity, Phylogenomic Contour, Putative Virulence Determinants, and Potential Diagnostic Markers within *Pythium insidiosum* Traits

**DOI:** 10.3390/jof9020169

**Published:** 2023-01-27

**Authors:** Weerayuth Kittichotirat, Thidarat Rujirawat, Preecha Patumcharoenpol, Theerapong Krajaejun

**Affiliations:** 1 Bioinformatics and Systems Biology Program, School of Bioresources and Technology and School of Information Technology, King Mongkut’s University of Technology Thonburi, Bangkhuntien, Bangkok 10150, Thailand; 2 Systems Biology and Bioinformatics Research Group, Pilot Plant Development and Training Institute, King Mongkut’s University of Technology Thonburi, Bangkhuntien, Bangkok 10150, Thailand; 3 Research Center, Faculty of Medicine, Ramathibodi Hospital, Mahidol University, Bangkok 10400, Thailand; 4 Interdisciplinary Graduate Program in Bioscience, Faculty of Science, Kasetsart University, Bangkok 10900, Thailand; 5 Department of Pathology, Faculty of Medicine, Ramathibodi Hospital, Mahidol University, 270 Rama 6 Road, Bangkok 10400, Thailand

**Keywords:** *Pythium insidiosum*, pythiosis, genome, diversity, phylogenomics, diagnostic marker

## Abstract

*Pythium insidiosum* has successfully evolved into a human/animal filamentous pathogen, causing pythiosis, a life-threatening disease, worldwide. The specific rDNA-based genotype of *P. insidiosum* (clade I, II, or III) is associated with the different hosts and disease prevalence. Genome evolution of *P. insidiosum* can be driven by point mutations, pass vertically to the offspring, and diverge into distinct lineages, leading to different virulence, including the ability to be unrecognized by the host. We conducted comprehensive genomic comparisons of 10 *P. insidiosum* strains and 5 related *Pythium* species using our online “Gene Table” software to investigate the pathogen’s evolutionary history and pathogenicity. In total, 245,378 genes were found in all 15 genomes and grouped into 45,801 homologous gene clusters. Gene contents among *P. insidiosum* strains varied by as much as 23%. Our results showed a strong agreement between the phylogenetic analysis of 166 core genes (88,017 bp) identified across all genomes and the hierarchical clustering analysis of gene presence/absence profiles, suggesting divergence of *P. insidiosum* into two groups, clade I/II and clade III strains, and the subsequent segregation of clade I and clade II. A stringent gene content comparison using the *Pythium* Gene Table provided 3263 core genes exclusively presented in all *P. insidiosum* strains but no other *Pythium* species, which could involve host-specific pathogenesis and serve as biomarkers for diagnostic purposes. More studies focusing on characterizing the biological function of the core genes (including the just-identified putative virulence genes encoding hemagglutinin/adhesin and reticulocyte-binding protein) are needed to explore the biology and pathogenicity of this pathogen.

## 1. Introduction

*Pythium insidiosum* is an underrecognized oomycete pathogen that belongs to the family Pythiaceae, order Pythiales, phylum Oomycota, clades Stramenopiles/Sar of superkingdom Eukaryota (https://www.ncbi.nlm.nih.gov/taxonomy). It is an etiological agent of a life-threatening infectious disease called pythiosis, reported worldwide, primarily in tropic and subtropic areas [1,2,3,4]. The unavailability of an established diagnostic tool in clinical laboratories and the lack of test efficiency make the detection of *P. insidiosum* in patients challenging [5,6,7,8,9,10,11,12,13,14,15,16]. Because the use of antimicrobial drugs is often ineffective, urgent radical surgery is required to treat pythiosis, aiming for a cure [2,5,17,18]. Patients usually lose an infected organ (i.e., eye, arm, or leg) due to the infection, and many die due to progressive disease [1,2,5]. A novel, feasible, and efficient method for diagnosing and treating pythiosis is needed.

Understanding the biology and pathogenicity of *P. insidiosum*, which is lacking for this pathogen, could lead to the discovery of a better disease-prevention, detection, and control method. Since most pathogenic oomycetes affect plant hosts, how *P. insidiosum* has adapted itself to be a successful human/animal pathogenic oomycete remains an open question [19,20]. Evolutionary processes must play a significant role in this adaptation, which could be traced for a clue by exploring the genome contents. Recent advances in next-generation sequencing technologies allow robust generation of genome data for an organism of interest at high efficiency, short duration, and low cost [21]. So far, the genome sequences of at least 10 genetically and geographically diverse strains of *P. insidiosum* have been sequenced and are publicly accessible [22,23,24,25,26,27,28]. Such genome data provide an opportunity to investigate the evolutionary history, biology, and virulence of *P. insidiosum*.

The current study employed a comparative genomic approach, using a user-friendly bioinformatic software called “Gene Table” developed by our group [19,29], to explore the evolutionary relationships within the *P. insidiosum* lineage. The genomes of several closely-related *Pythium* species (serving as phylogenetic outgroups), such as *Pythium aphanidermatum*, *Pythium arrhenomanes*, *Pythium irregulare*, *Pythium iwayamai*, and *Pythium ultimum* [19,30,31], were incorporated in the comparative analysis to identify species-specific genes of *P. insidiosum*, which could be involved in pythiosis and pathogen adaptation. We also attempted to better understand strain-to-strain genomic variations and identify core genes among different *P. insidiosum* strains/clades, which could potentially be involved in different pathogenesis mechanisms and serve as potential diagnostic and therapeutic markers. We herein present the results.

## 2. Methods

### 2.1. Genome Sequences of P. insidiosum and Related Species

The next-generation-sequencing-derived genomes of 10 *P. insidiosum* strains isolated from humans, animals, and the environment of different locations around the world, as well as 5 other *Pythium* species (i.e., *P. irregulare* strain DAOM BR486, *P. ultimum* strain DAOM BR144, *P. iwayamai* strain DAOM BR242034, *P. aphanidermatum* strain DAOM BR444, and *P. arrhenomanes* strain ATCC 12531) from the public repository, were recruited and used in this study (Table 1). The rDNA ITS-based genotype (i.e., clade I, II, or III) was assigned to all *P. insidiosum* strains. The resulting contig sequences were subjected to gene prediction using the MAKER2 pipeline [32]. All predicted protein sequences were annotated by comparing them to the NCBI non-redundant database using BLASTP [33]. 

### 2.2. Homologous Gene Clusters for Gene-Content Comparison

We compared gene contents across multiple genomes to investigate their genomic variability. All genes found in the genome of 10 *P. insidiosum* strains, together with the genome of 5 other *Pythium* species (Table 1), were subjected to a sequence-similarity-based gene-grouping process using our previously published protocol [35]. We used the following thresholds for each sequence comparison to group genes into the same cluster: BLAST *E*-value of 10^−6^, pairwise sequence identity of at least 30%, and pairwise sequence alignment coverage for both query and subject of at least 50%. These lenient criteria allowed the grouping of distant homologs and helped to minimize false positives in identifying group- or genome-specific genes (only present in a subset of genomes). With such criteria, if a particular gene is still not found in a genome, it is very likely that the gene is truly absent as opposed to present but may be significantly diverged from its corresponding orthologous genes found in other genomes. The final homologous gene cluster result is presented in table format, namely the *Pythium* Gene Table, where each row represents a gene and columns represent the 15 *Pythium* genomes used in this study. Each cell in the table contains information regarding homologous genes or genomic regions found in the corresponding genome.

### 2.3. Core Gene-Based Phylogenomic Analysis

A total of 166 core genes in the genomes of 10 *Pythium insidiosum* strains and 5 other *Pythium* species (Table S1) were used for phylogenetic analysis. These genes were selected by scanning through our homologous gene cluster data for genes present in all 15 genomes that did not possess length variations of more than 45% relative to the longest representative gene in each cluster (or each row in the *Pythium* Gene Table). Nucleotide sequences of each gene from all genomes were aligned using ClustalW version 2 with default parameters [36]. All ClustalW alignment results were then subjected to gap removal and concatenated to produce a single multiple-sequence alignment file. FastTree2 was then used to create a maximum-likelihood tree [37]. A bootstrap analysis was carried out to test the reliability of the tree. Finally, the phylogenetic tree was visualized using FigTree v1.4.4 (http://tree.bio.ed.ac.uk/software/figtree/; accessed on 4 July 2022).

### 2.4. Hierarchical Clustering of the Gene-Presence Profile Data

Our homologous gene cluster result was used to create gene-presence profile data. Values 0 and 1 denoted a gene absence and presence in each genome, respectively. We also set 1 (present) to cases where a gene was not predicted, but a homologous genomic region was found in that genome. Hierarchical clustering of the gene-presence profile data was done using MeV 4.8.1 with default parameters [38]. Finally, the FigTree software was used to draw the hierarchical clustering tree.

### 2.5. Annotation of P. insidiosum-Specific Genes

All translated protein sequences of the genes found only in *P. insidiosum* (*P. insidiosum*-specific)*,* but no other *Pythium* species, were BLAST searched against the NCBI nonredundant protein database (https://www.ncbi.nlm.nih.gov/; accessed on 22 September 2022) to assign the possible function to each protein. The same set of protein sequences were also subjected to a BLAST search against the MvirDB database [39] to identify putative virulence factors in *P. insidiosum*.

## 3. Results and Discussion

### 3.1. Genome Summary and Homologous Gene Cluster Data

The genome data of 10 *P. insidiosum* strains used in this study are summarized in Table 1. These *P. insidiosum* strains were isolated from humans, animals, and the environment, and the genotyping result shows that they cover major clades of the pathogen, including clades I (n = 3), II (n = 5), and III (n = 2). The genome sizes and the number of protein-coding genes of *P. insidiosum* range from 34,541,218 to 65,230,783 bp (mean 44,104,257 ± 9,918,608 bp) and 13,249 to 26,058 genes (mean 17,526 ± 4461 genes), respectively. Nevertheless, the guanine–cytosine percentages (G+C content) in these different *P. insidiosum* genomes were very similar (mean, 57.5%; range, 57.1–57.9%), which was generally higher than that of the other 5 *Pythium* species (mean, 54.4%; range, 52.3–56.9%) and could be considered as a genetic characteristic of this human/animal pathogenic oomycete. On average, the genome sizes of the strains from clade III (56,186,238 bp) appeared to be larger than that of clades I (43,342,325 bp) and II (39,728,624 bp). As shown here, different *P. insidiosum* strains exhibited a high degree of variability in genome size and gene contents, which could give rise to phenotypic and pathogenic diversity between strains of this microbe. For example, the clade I strains are more specific to animals, while the clade II and III strains are notably infectious to humans, showing different clinical features and outcomes [1,6]. More analyses based on a larger number of isolates/strains from all known clades should be carried out to better understand these within-species genomic variations and confirm these associations.

A total of 245,378 genes was found in the genomes of 10 *P. insidiosum* strains and 5 other *Pythium* species (i.e., *P. irregulare*, *P. ultimum*, *P. iwayamai*, *P. aphanidermatum*, and *P. arrhenomanes*). These genes could be grouped into 45,801 unique homologous gene clusters. The homologous gene cluster data used in this study can be assessed using our online tool, *Pythium* Gene Table, which can be found at https://202.28.6.19/cgi-bin/gt/viewer?organism=pythium&build=191128 (accessed on 11 May 2022). A detailed description and instruction manual for the Oomycete Gene Table software, previously reported by our group [29], can be adapted for utilizing the *Pythium* Gene Table. The total number of homologous gene clusters (n = 45,801) is significantly larger than the number of genes in any single genome (ranging from 12,312 to 26,058 genes), suggesting a high degree of gene-content variation across these organisms.

### 3.2. Evolutionary Relationship between P. insidiosum Strains

**Figure 1:**
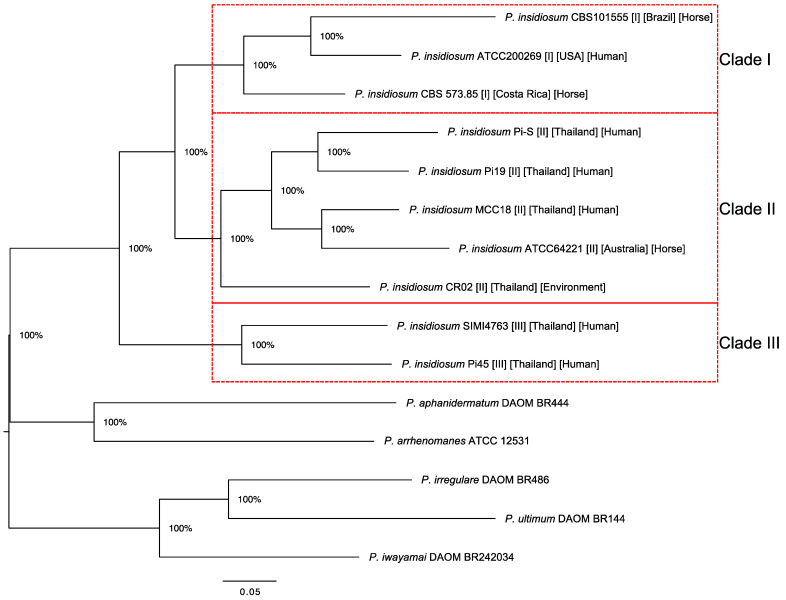
A phylogenetic tree based on 166 single-copy core genes in the genomes of 10 *P. insidiosum* strains and 5 other *Pythium* species. The bootstrap values, used to indicate the reliability of the result, are shown on each branching node. Red boxes depict groups of the *P. insidiosum* strains assigned to rDNA-based genotype clades I, II, and III.

**Figure 2:**
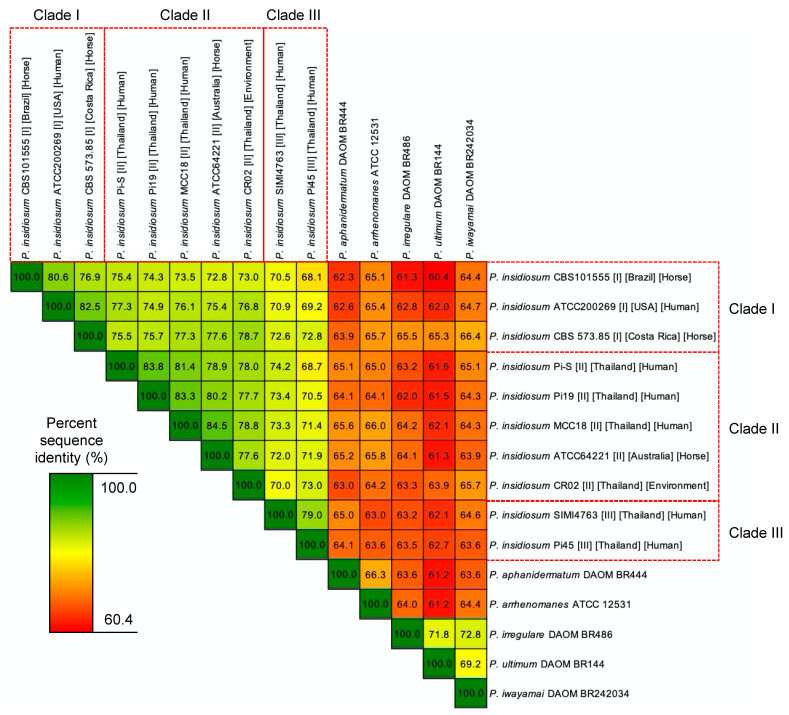
Percent sequence identities between pairs of the genomes from 10 *P. insidiosum* strains and 5 other *Pythium* species based on 166 single-copy core gene sequences. Red boxes show the *P. insidiosum* strains assigned to rDNA-based genotype clades I, II, and III. Color gradience indicates the degree of sequence identity (%).

To investigate the evolutionary relatedness and distinction among various strains of *P. insidiosum* and between *P. insidiosum* and other *Pythium* species, a phylogenetic analysis was performed using a set of 166 core genes found across all organisms. These genes are summarized in Table S1. Concatenation of 166 gap-removed multiple sequence alignments produced a final multiple sequence alignment result of 88,017 bases in length. The maximum-likelihood tree made from this multiple sequence alignment is shown in Figure 1. As expected, *P. insidiosum* strains formed a cluster segregated from other *Pythium* species. Our phylogenetic tree also separated all *P. insidiosum* strains according to their assigned clades I, II, or III. In line with this finding, strains from the same clade exhibited higher percent sequence identity within these 166 core gene sequences, as shown in Figure 2. Specifically, the percent sequence identities among strains within clades I, II, and II ranged between 76.9–82.5% (average, 80.0%), 77.6–84.5% (average, 80.4%), and 79%, respectively. On the other hand, when *P. insidiosum* strains of different clades were compared, the percent sequence identities were generally lower: range, 68.1–78.7%; average, 73.4%. Besides, if comparing *P. insidiosum* and other *Pythium* species, the percent sequence identities significantly dropped to less than 66.5%. Based on these analyses, *P. insidiosum* strains from clade I appeared to be evolutionarily closer to strains from clade II, suggesting a major evolutionary division of clade III strains and the group of clade I and II strains. Additional studies that include more strains from these clades can be carried out to confirm this finding and rule out possible sampling effects.

### 3.3. Gene Content of P. insidiosum

**Figure 3:**
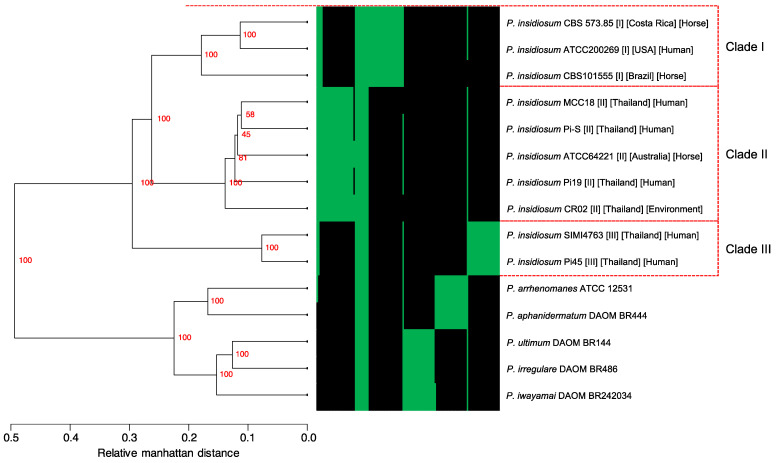
A dendrogram showing the hierarchical clustering result based on 45,801 gene-presence profiles across 10 *P. insidiosum* strains and 5 *Pythium* species. The presence (green) or absence (black) status of 120 selected genes are shown as a heat map next to the dendrogram. The bootstrap values are shown on each dendrogram branching node. Red boxes depict groups of the *P. insidiosum* strains assigned to rDNA-based genotype clades I, II, and III. This result shows that *P. insidiosum* strains from the same clade share higher gene-content similarity.

We next investigated the gene content relatedness and distinction among *P. insidiosum* strains and other *Pythium* species by exploring the presence and absence of each of the 45,801 homologous gene clusters (see above) across the genomes. The result of the hierarchical clustering analysis of gene presence/absence profiles is shown in Figure 3. Consistent with the core-gene-based phylogenetic analysis (Figure 1), the obtained dendrogram showed similar patterns of segregation where *P. insidiosum* strains were distinctive from other *Pythium* species. Besides, the *P. insidiosum* strains formed three major clades, in which the strains from clades I and II revealed similar gene contents and were closely related compared to those in clade III.

**Figure 4:**
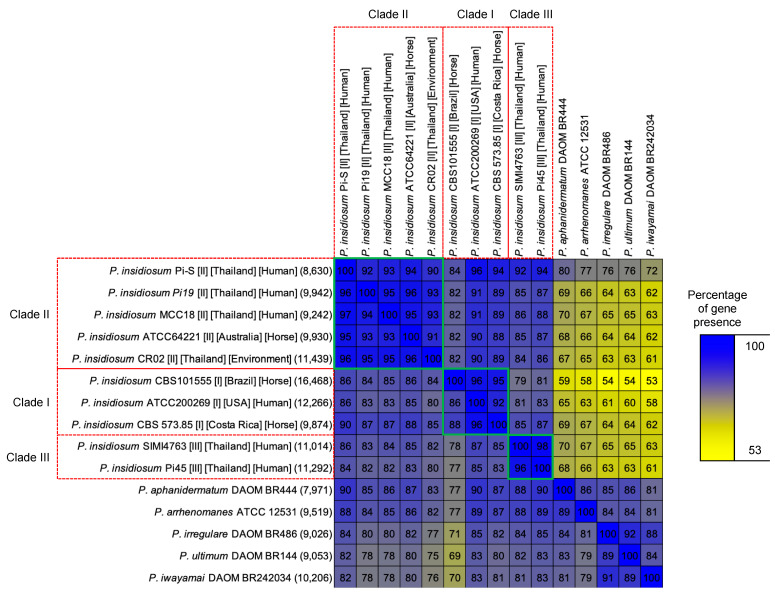
All pairwise gene content comparisons of 10 *P. insidiosum* strains and 5 other *Pythium* species. The number in each parenthesis indicates the total number of non-redundant genes in the genome of each organism used in this analysis. The number in each cell shows the percentage of genes present in the genome shown on the left that is also present in the corresponding genome shown at the top of the table. Red boxes show the *P. insidiosum* strains assigned to rDNA-based genotype clades I, II, and III. Green boxes demonstrate the percent gene presence within the same clade. Color gradience indicates the degree of gene presence (%).

All-against-all comparisons of gene content among pairs of genomes are shown in Figure 4. The gene-content similarity between a pair of genomes was summarized by calculating the percentage of genes in one genome (shown by the row label) that was also present in the other genome (shown by the column label). As demonstrated here, *P. insidiosum* strains from the same clade exhibited high gene-content similarity, ranging from 86% to 98%, with an average of 94% (shown within green squares in Figure 4). This pattern of gene-content similarity correlated with core gene sequence identity as described above (Figure 2). As expected, the similarity in *P. insidiosum*’s gene content decreased as a strain from one clade was compared to a strain from a different clade. Specifically, the gene-content similarity between strains from different clades was between 77% and 96 (average of 85%). Finally, the gene-content similarity between *P. insidiosum* and other *Pythium* species was between 53% and 80% (average, 65%), showing the level of gene content uniqueness of *P. insidiosum* relative to the other *Pythium* species. Such different and unique gene content of *P. insidiosum*, compared with the other *Pythium* species, could be associated with human and animal pathogenicity. This is worth further exploration to identify virulence genes.

### 3.4. Core and Variable Genes of Pythium insidiosum

**Figure 5:**
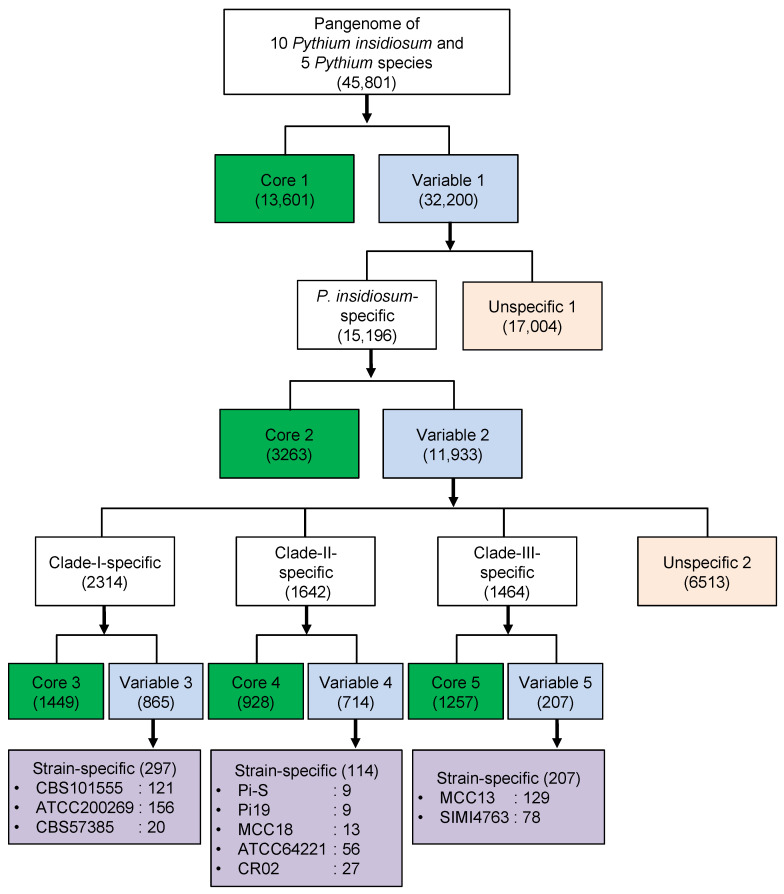
Core and variable genes among 10 *P. insidiosum* strains and 5 other *Pythium* species. Green boxes represent core genes (i.e., Cores 1, 2, 3, 4, and 5) present in all genomes at each level. Light blues show variable genes (i.e., Variables 1, 2, 3, 4, and 5), which present at least one but not all of the genomes at each level. Variable genes can be classified into species-specific, clade-specific, strain-specific, and unspecific groups.

Genome sequence data revealed that different *P. insidiosum* strains possessed a high level of genomic variation. These variations may result from gene acquisitions and deletions as different strains have evolved and adapted to different environments, and consequently gave rise to the modern *P. insidiosum.* Here, we hypothesized that preserved genes might have functional features distinct from genes that are variably present in different genomes. Therefore, we classified the 45,801 unique genes found across the genomes of 10 *P. insidiosum* and 5 other *Pythium* species based on their distribution patterns, as shown in Figure 5.

**Figure 6:**
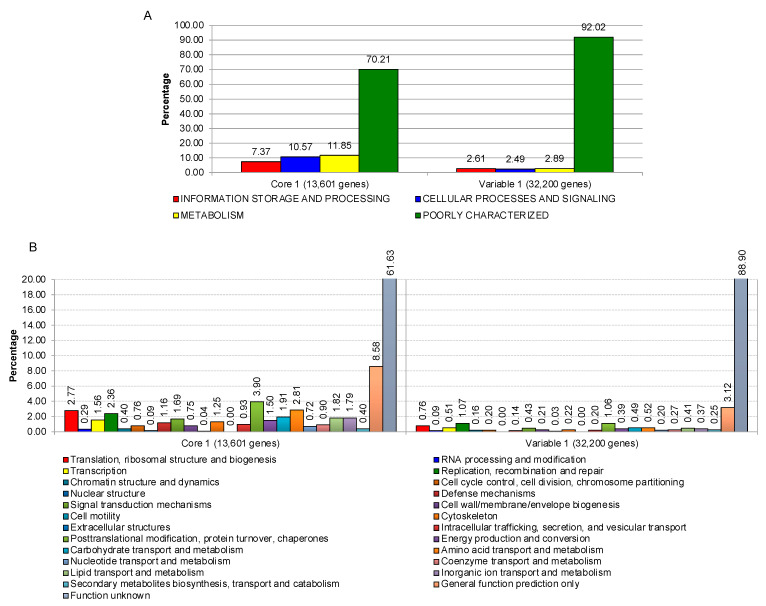
Functional classification of Core 1 and Variable 1 genes, derived from the pangenome analysis of 10 *P. insidiosum* strains and 5 other *Pythium* species, based on clusters of orthologous groups (COG) at superfunctional (**A**) and functional (**B**) levels.

First, we classified genes into a Core 1 group (13,601 genes), which consists of genes that are found in every strain of *P. insidiosum* as well as other included *Pythium* species, and a Variable 1 group (32,200 genes), which is made up of accessory genes that are found in some but not all genomes. Figure 6 shows the functional overview of both groups using functional categories from clusters of orthologous groups (COG) at both superfunctional (Figure 6A) and functional (Figure 6B) levels. More genes from the Variable 1 group were categorized as poorly characterized (92.02% or 29,631 genes) when compared to the Core 1 group (70.21% or 9,549 genes). Since core genes found in all strains and across different species are most likely involved in the essential housekeeping functions, these genes are usually well-studied and characterized. This is shown in our result, where 29.79% of the Core 1 genes can be functionally annotated as opposed to 7.98% of genes in the Variable 1 group (Figure 6A).

Next, we classified the Variable 1 genes into genes found exclusively in *P. insidiosum* but no other species (*P. insidiosum*-specific; 15,196 genes) and otherwise (unspecific 1; 17,004 genes). The *P. insidiosum*-specific genes were further classified into Core 2 (genes found in all *P. insidiosum* genomes; 3263 genes) and Variable 2 (genes found in some *P. insidiosum* genomes; 11,933 genes), as shown in Figure 5. Core 2 and Variable 2 gene groups were generally dominated (more than 95%) by poorly characterized genes based on the COG assignment (Figure S1). We further classified Variable 2 genes into clade-specific and strain-specific genes (Figure 5). As expected, these genes were mostly annotated as poorly characterized based on the COG assignments (Figures S2–S4). Few strain-specific genes, ranging from 9 to 156, were found in each *P. insidiosum* genome, suggesting microevolution existed within the different traits.

*P. insidiosum*-specific core genes (Core 2) are of particular interest as they may provide new insight into the virulence mechanism of this pathogen. In addition, due to their uniqueness, the Core 2 genes may be useful as potential targets for detecting various *P. insidiosum* strains, aiming at improved pythiosis diagnosis. We, therefore, performed a more comprehensive analysis by comparing these 3263 Core 2 genes to the NCBI database to gain additional functional information. Our analysis showed that 1386 (42.5%) genes had hits in the NCBI database (Table S2). Approximately 71.1% of all NCBI hits (986 genes) were described as hypothetical, uncharacterized, or unnamed proteins. We also BLAST searched the Core 2 genes against a microbial virulence factor collection, called the MvirDB database, which contains ~30,000 records assigned as virulence factor IDs (VFID) [39]. Of 3263 Core 2 genes, 53 (1.6%) significantly matched 41 VFIDs, representing putative virulence factors that are shared among genetically diverse *P. insidiosum* strains (Table S3). Among the MvirDB hits, our attention was focused on two putative virulence factors—the reticulocyte-binding protein of *Plasmodium falciparum* (VFID26643, matched by five *P. insidiosum* genes: p-cluster326349, p-cluster293767, p-cluster249718, p-cluster350032, and p-cluster245167) and the hemagglutinin/adhesin of *Bordetella pertussis* (VFID7841, matched by three *P. insidiosum* genes: p-cluster278911, p-cluster346852, and p-cluster288943). Since patients with vascular pythiosis usually present with arterial occlusion partly caused by blood-clot formation, resulting in a gangrenous leg [2,5], these putative hemagglutinins/adhesins and reticulocyte-binding proteins identified in *P. insidiosum* might be involved in such pathology. Further studies that aim to elucidate the functions of these *P. insidiosum*-specific core virulent genes are therefore needed to better understand the pathogenesis of this microbe.

## 4. Conclusions

The availability of genome data of diverse *P. insidiosum* strains has suggested that this species comprises several genetically distinct groups of strains. The rDNA ITS-based genotyping data (i.e., clade I, II, or III) has shown evidence that specific strains are associated with a different susceptible host and disease prevalence [1,6,40,41], suggesting possible variable virulence potential and mechanisms among different strains.

The main objective of this study was to carry out comprehensive genomic comparisons of *P. insidiosum* strains to provide a valuable and organized genomic dataset for further investigation into the potential virulence mechanism of this pathogen. Regarding the evolutionary relationship among strains, our results showed a strong agreement between phylogenetic analysis based on core genes and clustering analysis based on gene content. Specifically, our results suggest a divergence of two groups represented by clade I/II and clade III strains and the subsequent segregation of clade I and clade II. Since this divergence pattern was also observed in gene-content distribution, the gain and loss of genes may have occurred early in the evolution and become fixed in the genome.

The gene content in the *P. insidiosum* genome can vary as much as 23% between strains. Therefore, thousands of genes may present in one strain but not another. While this might be a result of a genetic abnormality such as aneuploidy, further investigations are needed to explore this possibility. Nevertheless, this extensive gene-content variation can potentially give rise to numerous biological and pathogenic properties among *P. insidiosum* strains. Therefore, different strategies for adaptation to the human host may have evolved and be employed by different lineages of *P. insidiosum*. While the genes exclusively present in all *P. insidiosum* strains but not any other species (i.e., Core 2 genes), including just-identified (i.e., hemagglutinin/adhesin and reticulocyte-binding protein) and known (i.e., elicitin [42,43]) putative virulence factors, the actual biological functions of these genes are still unknown. Some of these genes, as well as the clade-specific genes, may encode novel virulence factors. In addition, some of the Core 2 genes could serve as pan-*P. insidiosum* biomarkers for developing a more efficient diagnostic tool. More studies focusing on characterizing the biological function of these genes are needed to explore this possibility.

## Figures and Tables

**Table 1:** Genome summary of 10 *P. insidiosum* strains and 5 other *Pythium* species (i.e., *P. irregulare*, *P. ultimum*, *P. iwayamai*, *P. aphanidermatum*, and *P. arrhenomanes*) used in this study.

Organisms and Strains (Genotype: clade I, II, or III)	Country of Origin	Source	Number of Contigs	Total Contig Length (bp)	G+C Content (%)	Numbers of Protein Coding Genes	Total Coding Sequence Length	Average CDS Size (bp)	Coding Density (%)	N50 Length (bp)	Accession Number [Reference]
*P. insidiosum* CBS 573.85 (I)	Costa Rica	Horse	11,223	35,561,321	57.7	14,487	18,305,243	1264	51	12,261	BCFO00000000.1 [24]
*P. insidiosum* CBS 101555 (I)	Brazil	Horse	60,602	48,855,945	57.3	23,254	15,797,443	679	32	953	BCFP00000000.1 [25]
*P. insidiosum* ATCC200269 (I)	USA	Human	8992	45,609,708	57.3	20,359	31,572,237	1551	69	13,382	BCFN00000000.1 [23]
*P. insidiosum* Pi-S (II)	Thailand	Human	1192	53,239,050	57.9	14,962	22,867,201	1528	43	146,252	BBXB00000000.1 [28]
*P. insidiosum* Pi19 (II)	Thailand	Human	14,576	35,372,432	57.2	13,895	16,104,157	1159	46	6208	BCFS00000000.1 [23]
*P. insidiosum* MCC18 (II)	Thailand	Human	11,084	34,541,218	57.2	13,249	16,709,445	1261	48	8946	BCFT00000000.1 [23]
*P. insidiosum* CR02 (II)	Thailand	Environment	22,560	37,673,126	57.1	15,231	15,695,021	1030	42	3553	BCFR00000000.1 [24]
*P. insidiosum* ATCC64221 (II)	Australia	Horse	13,060	37,817,292	57.6	14,424	17,868,042	1239	47	11,370	BCFQ01000000.1 [22]
*P. insidiosum* SIMI4763 (III)	Thailand	Human	15,162	47,141,692	57.6	19,340	24,053,325	1244	51	11,187	BCFU00000000.1 [23]
*P. insidiosum* Pi45 (III)	Thailand	Human	17,277	65,230,783	57.8	26,058	33,835,683	1298	52	14,374	BCFM00000000.1 [26]
*P. arrhenomanes* ATCC 12531	-	-	10,972	44,672,625	56.9	13,805	18,531,402	1342	41	9784	AKXY00000000.2 [31]
*P. irregulare* DAOM BR486	-	-	5887	42,968,084	53.8	13,805	20,666,825	1497	48	23,217	JAADWQ000000000.1 [34]
*P. iwayamai* DAOM BR242034	-	-	11,542	43,200,612	55.1	14,875	19,758,311	1328	46	11,008	AKYA00000000.2 [31]
*P. ultimum* DAOM BR144	-	-	975	44,913,582	52.3	15,322	20,145,968	1315	45	837,833	ADOS00000000.1 [30]
*P. aphanidermatum* DAOM BR444	-	-	1774	35,876,849	53.8	12,312	18,112,821	1471	50	37,384	AKXX00000000.2 [31]

## Data Availability

The draft genome sequences of 10 *P. insidiosum* strains are retrievable from the DDBJ/NCBI databases through the accession numbers BCFO00000000.1, BCFP00000000.1, BCFN00000000.1, BBXB00000000.1, BCFS00000000.1, BCFT00000000.1, BCFR00000000.1, BCFQ01000000.1, BCFU00000000.1, and BCFM00000000.1. The draft genome data of 5 other *Pythium* species are also available in the DDBJ/NCBI databases through the accession numbers AKXX00000000.2 (*P. aphanidermatum*), AKXY00000000.2 (*P. arrhenomanes*), JAADWQ000000000.1 (*P. irregulare*), AKYA00000000.2 (*P. iwayamai*), and ADOS00000000.1 (*P. ultimum*).

## Supplementary Materials

The following supporting information can be downloaded at: https://www.mdpi.com/article/10.3390/jof9020169/s1, Figure S1: Functional classification of Core 2 and Variable 2 genes using Clusters of Orthologous Groups (COG) according to (A) Superfunctional and (B) Functional categories.; Figure S2: Functional classification of Core 3 and Variable 3 genes using Clusters of Orthologous Groups (COG) according to (A) Superfunctional and (B) Functional categories.; Figure S3: Functional classification of Core 4 and Variable 4 genes using Clusters of Orthologous Groups (COG) according to (A) Superfunctional and (B) Functional categories.; Figure S4: Functional classification of Core 5 and Variable 5 genes using Clusters of Orthologous Groups (COG) according to (A) Superfunctional and (B) Functional categories.; Table S1: Summary of 166 single copy core genes present in the genomes of 10 *P. insidiosum* strains and other 5 *Pythium* species. This set of genes is used in the phylogenetic analysis. All sequences can be accessed by searching our *Pythium* Gene Table Tool (https://202.28.6.19/cgi-bin/gt/viewer?organism=pythium&build=191128) using the Cluster ID.; Table S2: BLAST search hits of the *P. insidiosum*-specific Core 2 gene-translated proteins against the NCBI database.; Table S3: BLAST search hits of the *P. insidiosum*-specific Core 2 gene-translated proteins against the MvirDB database.
