# Supplementary material for: Comparative Genomic Analysis Reveals Gene Content Diversity, Phylogenomic Contour, Putative Virulence Determinants, and Potential Diagnostic Markers within Pythium insidiosum Traits
Source: J Fungi (Basel). 2023 Jan 27;9(2):169. doi: 10.3390/jof9020169 (PMC9962146; doi:10.3390/jof9020169)
Supplement: Supplementary file 1 [file jof-09-00169-s001.zip › 221128_Supplementary Figures S1-4 (PDF).pdf]

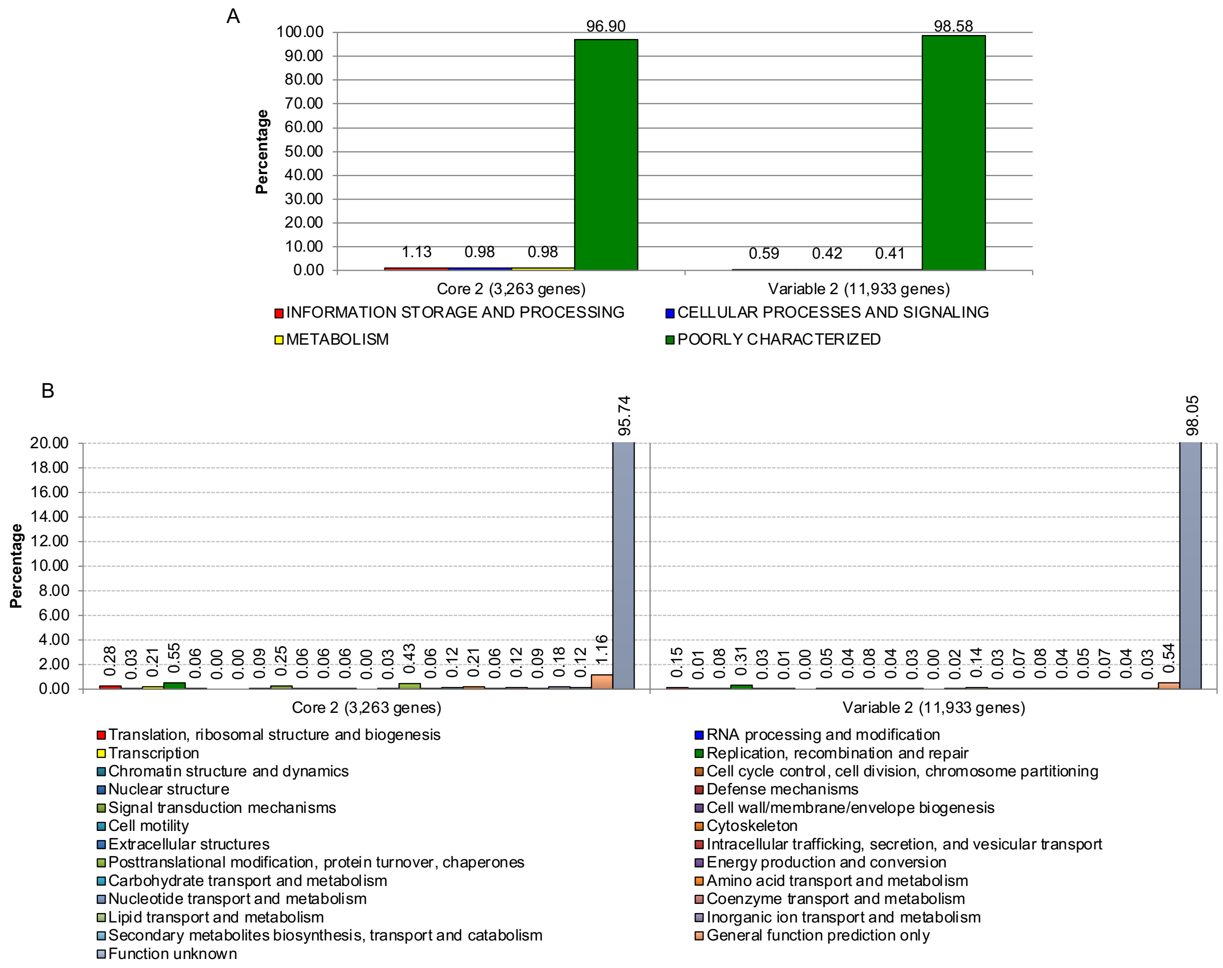

Supplementary Figure S1. Functional classification of Core 2 and Variable 2 genes using Clusters of Orthologous Groups (COG) according to (A) Superfunctional and (B) Functional categories.

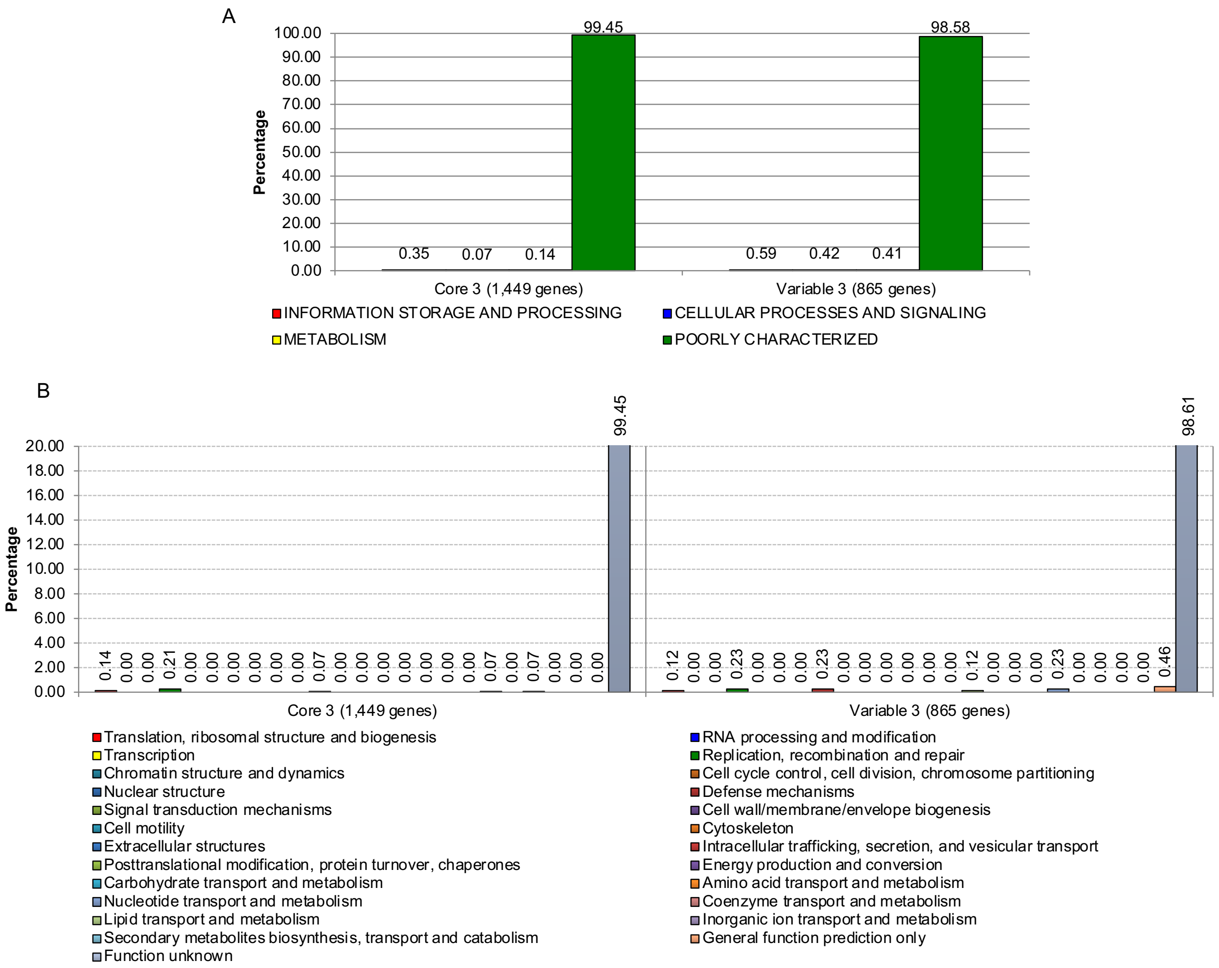

Supplementary Figure S2. Functional classification of Core 3 and Variable 3 genes using Clusters of Orthologous Groups (COG) according to (A) Superfunctional and (B) Functional categories.

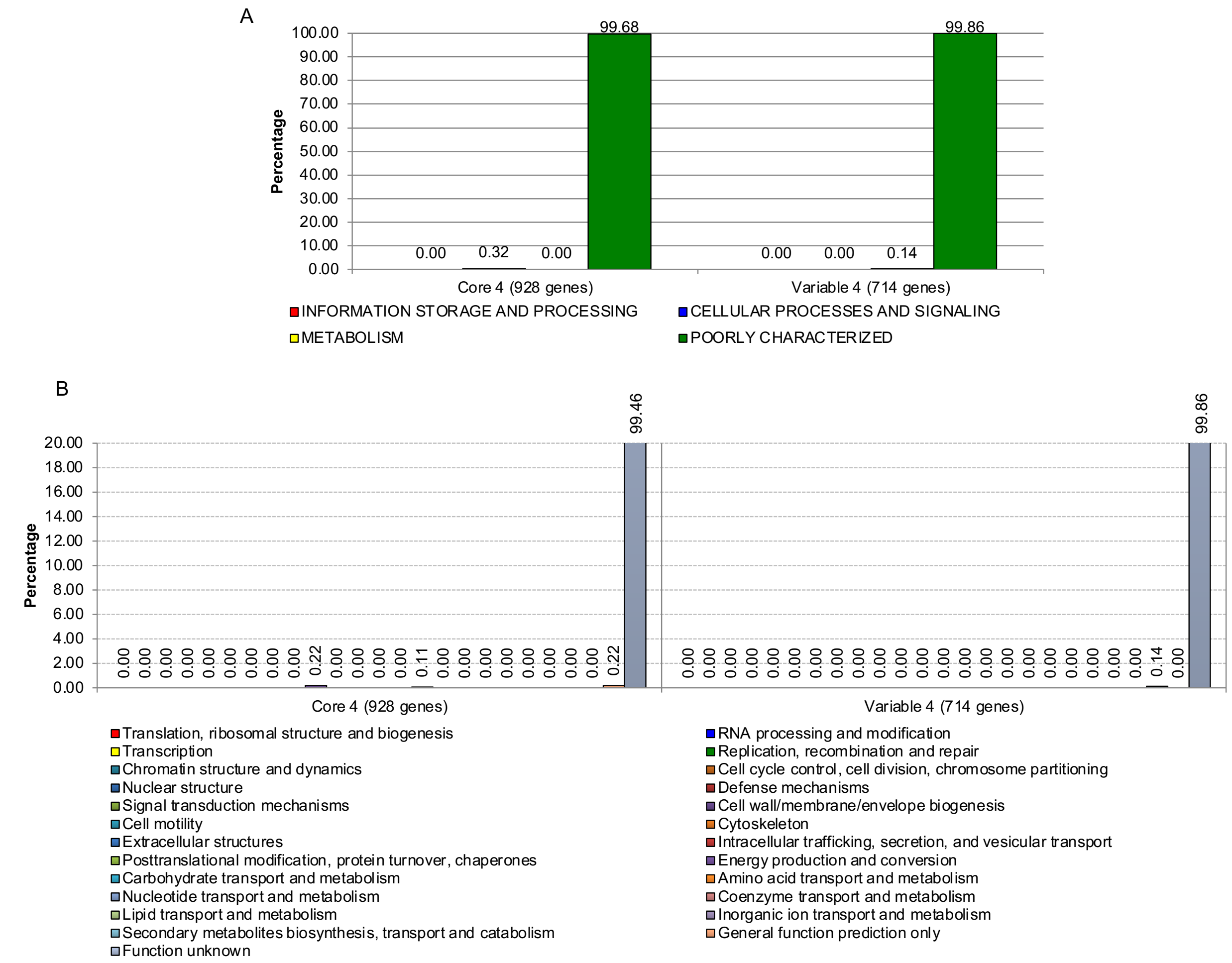

Supplementary Figure S3. Functional classification of Core 4 and Variable 4 genes using Clusters of Orthologous Groups (COG) according to (A) Superfunctional and (B) Functional categories.

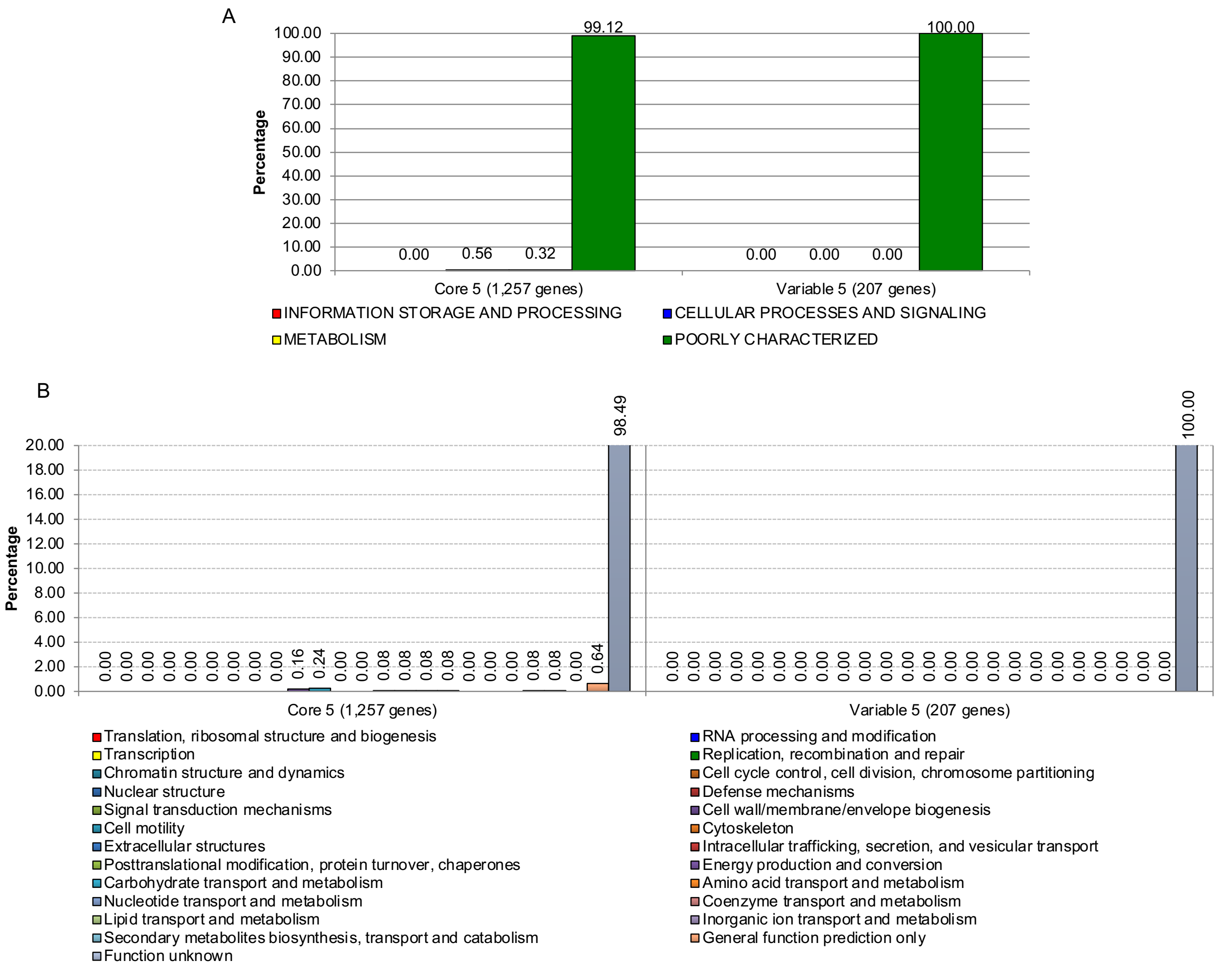

Supplementary Figure S4. Functional classification of Core 5 and Variable 5 genes using Clusters of Orthologous Groups (COG) according to (A) Superfunctional and (B) Functional categories.
