# Supplementary material for: Comparative Genomic Analysis Reveals Gene Content Diversity, Phylogenomic Contour, Putative Virulence Determinants, and Potential Diagnostic Markers within Pythium insidiosum Traits
Source: J Fungi (Basel). 2023 Jan 27;9(2):169. doi: 10.3390/jof9020169 (PMC9962146; doi:10.3390/jof9020169)
Supplement: Supplementary file 1 [file jof-09-00169-s001.zip › 221128_Supplementary Table S1 ok (PDF).pdf]

Supplementary Table S1. Summary of 166 single copy core genes present in the genomes of 10 *P. insidiosum* strains and other 5 *Pythium* species. This set of genes is used in the phylogenetic analysis. All sequences can be accessed by searching our *Pythium* Gene Table Tool (https://202.28.6.19/cgi-bin/gt/viewer?organism=pythium&build=191128) using the Cluster ID.

Cluster ID	Expected Length (bp)	Product Description
p-cluster058321	13248	Dynein heavy chain
p-cluster331378	9453	Pre-mrna-processing-splicing factor 8
p-cluster356241	6276	Dna-directed rna polymerase i subunit rpa2, putative
p-cluster039728	4905	Type i inositol 3
p-cluster116446	4761	P-glycoprotein
p-cluster471970	4602	Wd domain-containing protein
p-cluster045117	4422	Chromatin-remodeling complex atpase chain, putative
p-cluster339836	4251	Glycoside hydrolase, family 15:p (iss)
p-cluster279513	4179	Myosin
p-cluster130998	4125	Atp-binding cassette (abc) superfamily
p-cluster323678	4125	Rna polymerase-associated protein ctr9
p-cluster333890	4098	Elongation factor tu gtp-binding domain-containing protein 1
p-cluster051218	3954	Sphingosine/diacylglycerol kinase-like enzyme
p-cluster399594	3723	Alpha-glucosidase
p-cluster318336	3711	Argonaute4 (ago4)
p-cluster163438	3654	Guanylate-binding protein, n-terminal domain containing protein
p-cluster206380	3609	Beta-galactosidase 5
p-cluster525305	3549	Isoleucine-trna ligase
p-cluster076910	3540	Phosphatase 2c and cyclic nucleotide-binding/kinase domain-containing protein
p-cluster012200	3465	Elongation factor
p-cluster045331	3441	Choline dehydrogenase
p-cluster055029	3426	Nonsense-mediated mrna decay protein
p-cluster001065	3408	Importin
p-cluster053633	3360	Exportin-t, putative
p-cluster195878	3255	Glucose regulated protein /bip
p-cluster015208	3251	Membrane alanine aminopeptidase
p-cluster346298	3189	Atp-dependent helicase
p-cluster317760	3162	Gamma-aminobutyric acid type b receptor subunit 2
p-cluster475259	3156	Dna replication licensing factor mcm2
p-cluster361250	3090	Pc22g01270 protein
p-cluster328407	3042	Transposase
p-cluster269244	2931	Croquemort-like mating protein m82
p-cluster485873	2916	Protein kinase
p-cluster127561	2901	Serine protease family s33, putative
p-cluster389477	2847	Transmembrane protein
p-cluster472752	2847	Rac family serine/threonine-protein kinase
p-cluster107154	2844	Heat shock protein 101

p-cluster188262	2835	Beta-glucan synthesis-associated protein
p-cluster335763	2800	Peptidase
p-cluster366428	2775	Proton-dependent oligopeptide transporter (pot) family
p-cluster029102	2667	Ap-1 complex subunit gamma-1
p-cluster467699	2646	Vacuolar protein sorting-associated protein
p-cluster324621	2592	Filamentous muscle protein titin - homo sapiens
p-cluster216398	2562	Cytochrome p450
p-cluster221544	2532	Hypothetical protein
p-cluster343472	2532	Glucose transporter, putative
p-cluster064043	2496	Monoglyceride lipase
p-cluster428614	2481	Multicopper oxidase, types 2 and 3
p-cluster204602	2478	Transmembrane protein
p-cluster225249	2475	Lactoylglutathione lyase
p-cluster040513	2439	Hect e3 ubiquitin ligase
p-cluster209228	2412	Seryl-trna synthetase
p-cluster318916	2409	Abc transporter b family member 2
p-cluster150334	2406	Myosin
p-cluster214680	2398	Gk16278
p-cluster172856	2349	Flagellar outer dynein arm-docking complex protein 1
p-cluster261793	2310	Dynein intermediate chain 2
p-cluster464442	2307	Serine protease family s15
p-cluster015876	2277	Ars-binding protein
p-cluster055792	2277	Trna wybutosine-synthesizing protein
p-cluster052259	2211	Mitogen-activated protein kinase mmk1
p-cluster474738	2204	Dynamin-2
p-cluster468363	2172	Regulator of g-protein signalling 13
p-cluster465546	2166	Calcineurin-like phosphoesterase
p-cluster200538	2157	Manganese lipoxygenase
p-cluster172193	2130	Eukaryotic translation initiation factor 2-alpha kinase
p-cluster048812	2100	Bifunctional arginine demethylase and lysyl-hydroxylase psr
p-cluster478543	2043	Dna repair protein rada
p-cluster491652	1983	Atp-dependent dna helicase
p-cluster482351	1971	2-isopropylmalate synthase
p-cluster468864	1957	Cystathionine beta-lyase
p-cluster063775	1955	Cell 5a endo-1,4-betaglucanase
p-cluster035708	1947	Glycosyltransferase protein
p-cluster471444	1941	Succinate dehydrogenase
p-cluster049548	1926	Folate-biopterin transporter (fbt) family
p-cluster263090	1917	Acyltransferase family
p-cluster456928	1908	Guanine nucleotide-binding protein-like 3
p-cluster202210	1857	Ppat5
p-cluster044367	1851	Aspartyl-trna synthetase
p-cluster083996	1848	Lipoprotein
p-cluster314653	1836	Mitogen-activated protein kinase
p-cluster480091	1818	Aldehyde dehydrogenase family 7 member a1

p-cluster284283	1791	Glycoside hydrolase
p-cluster363995	1791	Conserved domain protein
p-cluster521855	1776	Dihydroxyacetone kinase
p-cluster032512	1760	Serine hydroxymethyltransferase, mitochondrial precursor
p-cluster078738	1752	Gtp-binding protein
p-cluster448144	1749	Tubulin epsilon chain
p-cluster456232	1746	Protein transporter sec61 subunit alpha
p-cluster098646	1716	Protein disulfide-isomerase
p-cluster156309	1701	Apopolysialoglycoprotein
p-cluster527580	1701	Ketol-acid reductoisomerase
p-cluster209791	1698	Diaminopimelate decarboxylase
p-cluster454427	1695	Cysteine protease family c01a
p-cluster216275	1647	Methyltransfer with n-terminal ankyrin repeats
p-cluster093883	1620	Endoplasmic oxidoreductin
p-cluster464180	1617	Carbohydrate-binding protein
p-cluster083707	1615	Protein arginine n-methyltransferase 1
p-cluster537893	1611	Myosin protein
p-cluster281601	1590	Aspartyl protease family a22b, putative
p-cluster244249	1589	Atpase
p-cluster088343	1578	Methionine aminopeptidase
p-cluster472207	1578	Rio kinase
p-cluster084750	1566	Putative glycosyll hydrolase family 7 protein
p-cluster363486	1545	Chaperone protein dnaj
p-cluster474794	1495	Dolichyl-diphosphooligosaccharide-protein glycosylltransferase subunit
p-cluster024898	1494	H/aca ribonucleoprotein complex subunit 4
p-cluster066675	1491	Alanine aminotransferase 2
p-cluster060842	1488	Hypothetical protein
p-cluster007982	1470	Mitochondrial carrier (bou s-adenosylmethionine carrier)
p-cluster144037	1461	Hypothetical protein
p-cluster238286	1461	Rcg36077
p-cluster494057	1458	Ruvb-like dna helicase tip49b
p-cluster533270	1424	Dtdp-glucose 4,6-dehydratase, partial
p-cluster336520	1413	Ring finger protein 2
p-cluster061982	1374	Secreted protein
p-cluster221716	1371	Trna (guanine-n(1)-)-methyltransferase
p-cluster050283	1368	Elmo domain-containing protein 1
p-cluster057388	1299	Neuroguidin-like protein
p-cluster040839	1278	Gpi-anchored serine rich tenascin-like glycoprotein
p-cluster058644	1272	Heat shock protein 40 like protein putative
p-cluster382712	1257	Aquaporin-9-like protein
p-cluster043843	1224	Hsftype dnabinding protein putative
p-cluster070734	1221	Rxlr-like protein
p-cluster142396	1194	Abc transporter a family member 1
p-cluster106683	1155	Zinc (zn2)-iron (fe2) permease (zip) family
p-cluster325048	1155	Cell cycle checkpoint protein hrad1

p-cluster382026	1147	Replication factor c subunit 4
p-cluster052799	1146	Sbds family rrna metabolism protein
p-cluster474914	1143	Flagellar associated protein
p-cluster387232	1140	Alcohol dehydrogenase
p-cluster038257	1128	Splicing factor spf30
p-cluster058602	1119	Ribosomal rna methyltransferase
p-cluster460559	1098	Wd domain-containing protein, putative
p-cluster055760	1068	G protein alpha subunit
p-cluster423472	1056	Mitochondrial carrier (mc) family
p-cluster334604	1038	Sulfate permease (sulp) family
p-cluster236500	1023	Nitrilase/cyanide hydratase and apolipo protein n-acyltransferase-like protein
p-cluster297716	1020	Atp10 protein
p-cluster043368	994	14-3-3 protein epsilon
p-cluster486277	987	Phytanoyl-coa dioxygenase domain-containing protein, putative
p-cluster514879	987	Protein containing a histone methylation dot1 motif
p-cluster059281	960	Proteasome subunit beta, putative
p-cluster048080	912	Hypothetical protein
p-cluster055631	906	Phosphoglycerate mutase
p-cluster262726	876	Hypothetical protein
p-cluster340750	834	Nucleoside diphosphate kinase
p-cluster179849	831	An-nup2 nuclear pore complex protein (eurofung)
p-cluster072961	830	Bromodomain containing protein
p-cluster045576	828	Start domain containing protein
p-cluster045120	810	Scp-like extracellular protein
p-cluster028519	789	40s ribosomal protein s2
p-cluster456633	756	Electron transfer flavoprotein subunit beta
p-cluster539586	755	60s ribosomal protein l10
p-cluster227024	714	Nucleoredoxin
p-cluster005056	669	60s ribosomal protein l12
p-cluster459791	660	Hypothetical protein
p-cluster055734	624	Rdgb/ham1 family non-canonical purine ntp pyrophosphatase
p-cluster238070	621	Hypothetical protein
p-cluster102792	588	Hypothetical protein
p-cluster473236	555	Nop16
p-cluster042208	543	60s ribosome subunit biogenesis protein nip7
p-cluster476665	522	Prefoldin subunit
p-cluster041842	498	Hypothetical protein
p-cluster472019	451	Dynein light chain flagellar outer arm
p-cluster540466	397	13 kda ribonucleoprotein-associated protein
