# Supplementary material for: Comparative Genomic Analysis Reveals Gene Content Diversity, Phylogenomic Contour, Putative Virulence Determinants, and Potential Diagnostic Markers within Pythium insidiosum Traits
Source: J Fungi (Basel). 2023 Jan 27;9(2):169. doi: 10.3390/jof9020169 (PMC9962146; doi:10.3390/jof9020169)
Supplement: Supplementary file 1 [file jof-09-00169-s001.zip › 221128_Supplementary Table S2 (PDF).pdf]

Supplementary Table S2. BLAST search hits of the *P. insidiosum*-specific Core 2 gene-translated proteins against the NCBI database.

Query gene ID	Hit ID	Hit Description [Organism]	Alignment Sequence Identity (%)	Query Alignment Coverage (%)	Hit Alignment Coverage (%)
p-cluster001303	KAE8998458.1	hypothetical protein PF011_g15043 [Phytophthora fragariae]	50.00	83.02	61.29
p-cluster001728	TMW60999.1	hypothetical protein Poli38472_014460 [Pythium oligandrum]	50.00	77.27	7.92
p-cluster018222	TMW69373.1	hypothetical protein Poli38472_001529 [Pythium oligandrum]	62.93	71.65	74.32
p-cluster039179	TMW59523.1	hypothetical protein Poli38472_004592 [Pythium oligandrum]	44.39	100.00	100.00
p-cluster039866	KAG2788710.1	hypothetical protein Pcac1_g1988 [Phytophthora cactorum]	28.45	78.92	65.71
p-cluster050845	CCI42269.1	unnamed protein product [Albugo candida]	58.49	56.06	78.24
p-cluster054880	RLN81655.1	hypothetical protein BBJ28_00023336 [Nothophytophthora sp. Chile5]	44.44	40.98	92.71
p-cluster059876	ETK77921.1	hypothetical protein, variant 9 [Phytophthora parasitica]	34.79	73.56	90.72
p-cluster065519	KAE9260640.1	hypothetical protein PF008_g33055, partial [Phytophthora fragariae]	35.86	75.92	65.44
p-cluster087539	TMW61198.1	hypothetical protein Poli38472_013661 [Pythium oligandrum]	37.90	61.54	57.94
p-cluster100908	KAE8881855.1	hypothetical protein PF003_g34081 [Phytophthora fragariae]	36.91	94.19	57.65
p-cluster102076	TMW57199.1	hypothetical protein Poli38472_003124 [Pythium oligandrum]	51.41	99.59	7.50
p-cluster102759	TMW64513.1	hypothetical protein Poli38472_011393 [Pythium oligandrum]	37.88	93.80	90.93
p-cluster142193	GAX93486.1	leukotriene A-4 hydrolase/aminopeptidase [Pythium insidiosum]	92.86	100.00	30.52
p-cluster147664	KAE8971438.1	hypothetical protein PR002_g26828 [Phytophthora rubi]	38.14	71.32	100.00
p-cluster163192	GAX99532.1	CMGC/MAPK protein kinase [Pythium insidiosum]	86.32	91.33	44.42
p-cluster164819	GAY06237.1	30S ribosomal protein S5, putative [Pythium insidiosum]	92.90	100.00	53.99
p-cluster180945	TMW68037.1	hypothetical protein Poli38472_007709 [Pythium oligandrum]	36.46	96.15	17.08
p-cluster183229	TMW66283.1	hypothetical protein Poli38472_004048 [Pythium oligandrum]	64.06	44.87	28.65
p-cluster203568	RLN46173.1	hypothetical protein BBJ28_00024994, partial [Nothophytophthora sp. Chile5]	47.62	40.65	24.80
p-cluster204558	TMW62862.1	hypothetical protein Poli38472_005480 [Pythium oligandrum]	76.63	100.00	100.00
p-cluster204650	RLN86472.1	hypothetical protein BBJ28_00004694, partial [Nothophytophthora sp. Chile5]	47.44	16.89	39.90
p-cluster204835	KAG7391207.1	hypothetical protein PHYPSEUDO_005568 [Phytophthora pseudosyringae]	29.91	35.27	97.74
p-cluster204852	TMW63771.1	hypothetical protein Poli38472_002712 [Pythium oligandrum]	68.01	37.92	99.74
p-cluster204941	GAX92592.1	phox-like domain containing protein [Pythium insidiosum]	100.00	76.07	29.11
p-cluster204950	KAH7463611.1	hypothetical protein KRP23_12805 [Phytophthora ramorum]	49.14	20.42	63.33
p-cluster205114	GAX92781.1	TKL protein kinase [Pythium insidiosum]	100.00	100.00	100.00
p-cluster205359	TMW66178.1	hypothetical protein Poli38472_003943 [Pythium oligandrum]	46.43	98.25	18.30
p-cluster205371	RLN65146.1	hypothetical protein BBJ29_008150 [Phytophthora kernoviae]	36.02	77.33	44.88
p-cluster205709	TMW63406.1	hypothetical protein Poli38472_002347 [Pythium oligandrum]	58.86	54.74	16.18
p-cluster206806	TMW60227.1	hypothetical protein Poli38472_000269 [Pythium oligandrum]	69.52	58.53	93.88
p-cluster206897	GAX94545.1	choline dehydrogenase [Pythium insidiosum]	100.00	100.00	100.00
p-cluster207288	TMW64647.1	hypothetical protein Poli38472_011527 [Pythium oligandrum]	54.89	48.94	86.38
p-cluster207786	TMW67610.1	hypothetical protein Poli38472_011230 [Pythium oligandrum]	57.78	12.06	3.08
p-cluster207788	TMW67572.1	hypothetical protein Poli38472_011192 [Pythium oligandrum]	34.64	47.72	22.45
p-cluster207851	GAX95495.1	cysteine-rich protein [Pythium insidiosum]	81.67	88.24	86.03
p-cluster207871	TYZ61662.1	hypothetical protein PybrP1_010661 [[Pythium] brassicae (nom. inval.)]	69.23	100.00	100.00
p-cluster207936	GAX95617.1	peptidyl-prolyl cis-trans isomerase-like 3 [Pythium insidiosum]	100.00	100.00	100.00
p-cluster207937	GAX95626.1	transmembrane protein putative [Pythium insidiosum]	100.00	100.00	100.00
p-cluster207977	GAX95668.1	replication factor C subunit 5 [Pythium insidiosum]	100.00	100.00	100.00
p-cluster208074	GAX95775.1	60S ribosomal protein [Pythium insidiosum]	62.32	89.47	8.72
p-cluster208130	XP_008915474.1	hypothetical protein PPTG_18978 [Phytophthora parasitica INRA-310]	34.03	95.77	84.10
p-cluster208270	TMW67282.1	hypothetical protein Poli38472_012398 [Pythium oligandrum]	70.21	43.52	6.29
p-cluster208296	TMW66429.1	hypothetical protein Poli38472_004194 [Pythium oligandrum]	57.97	45.70	15.80
p-cluster208339	TMW66389.1	hypothetical protein Poli38472_004154 [Pythium oligandrum]	59.60	47.14	23.13
p-cluster208391	GAX96069.1	60S ribosomal protein L39-like protein [Pythium insidiosum]	100.00	100.00	100.00
p-cluster208491	ETP01743.1	hypothetical protein F441_21074 [Phytophthora parasitica CJ01A1]	25.00	82.22	62.63
p-cluster208867	TMW66766.1	hypothetical protein Poli38472_014078 [Pythium oligandrum]	38.50	85.64	83.12
p-cluster209018	TMW56063.1	hypothetical protein Poli38472_008711 [Pythium oligandrum]	56.63	68.60	35.04
p-cluster209655	XP_033123387.1	mucin-5AC-like, partial [Anneissia japonica]	48.77	26.49	20.80
p-cluster210093	TMW59388.1	hypothetical protein Poli38472_004457 [Pythium oligandrum]	63.60	52.03	87.36
p-cluster210140	TMW59280.1	hypothetical protein Poli38472_004349 [Pythium oligandrum]	78.57	99.12	98.58
p-cluster210292	TYZ60173.1	hypothetical protein PybrP1_012082 [[Pythium] brassicae (nom. inval.)]	86.89	95.31	100.00
p-cluster210431	GAX98074.1	TKL/DRK protein kinase [Pythium insidiosum]	77.24	100.00	85.26
p-cluster210653	GAX95883.1	pepsin-like aspartic protease A1 [Pythium insidiosum]	90.87	90.65	57.93

p-cluster210803	GAY06760.1	G-protein coupled receptor [Pythium insidiosum]	99.70	62.16	62.63
p-cluster210848	TMW67472.1	hypothetical protein Poli38472_011092 [Pythium oligandrum]	33.60	96.32	97.80
p-cluster210897	GAX98572.1	TKL protein kinase [Pythium insidiosum]	100.00	100.00	100.00
p-cluster211408	TMW55760.1	hypothetical protein Poli38472_010642 [Pythium oligandrum]	55.51	82.53	44.42
p-cluster211605	GAY04368.1	calpain-like protein [Pythium insidiosum]	70.27	57.81	4.44
p-cluster211849	GAX99548.1	glycine cleavage system T protein [Pythium insidiosum]	100.00	100.00	100.00
p-cluster211896	TMW58287.1	hypothetical protein Poli38472_011875 [Pythium oligandrum]	53.85	54.82	9.89
p-cluster212213	TMW68886.1	hypothetical protein Poli38472_001042 [Pythium oligandrum]	62.77	94.00	26.15
p-cluster212607	GAX96425.1	heat shock transcription factor [Pythium insidiosum]	63.89	100.00	68.84
p-cluster212859	KAF1315764.1	Mitochondrial protein, partial [Globisporangium splendens]	66.03	99.37	99.36
p-cluster212877	TMW62170.1	hypothetical protein Poli38472_009663 [Pythium oligandrum]	38.90	100.00	94.81
p-cluster213051	TMW64781.1	hypothetical protein Poli38472_011661 [Pythium oligandrum]	83.27	47.09	22.07
p-cluster213250	RMX64787.1	hypothetical protein DD238_006774 [Peronospora effusa]	51.79	67.09	31.46
p-cluster213566	TYZ64052.1	hypothetical protein PybrP1_011910 [[Pythium] brassicae (nom. inval.)]	87.18	95.12	96.30
p-cluster213600	TMW59944.1	hypothetical protein Poli38472_005013 [Pythium oligandrum]	58.68	33.80	38.46
p-cluster213797	TMW58043.1	hypothetical protein Poli38472_013517 [Pythium oligandrum]	47.47	63.68	60.72
p-cluster213849	TMW63618.1	hypothetical protein Poli38472_002559 [Pythium oligandrum]	38.40	94.70	6.12
p-cluster214160	TMW60882.1	hypothetical protein Poli38472_000924 [Pythium oligandrum]	27.24	83.98	41.79
p-cluster214439	GAY02110.1	elicitor-like transglutaminase [Pythium insidiosum]	100.00	100.00	100.00
p-cluster214765	GAY02426.1	ribonuclease T2 family protein [Pythium insidiosum]	98.86	100.00	100.00
p-cluster214900	GAY03407.1	phosphatidylinositol 3 and 4-kinase-like protein, partial [Pythium insidiosum]	100.00	92.19	7.41
p-cluster214954	RLN85082.1	hypothetical protein BBJ28_00001109 [Nothophytophthora sp. Chile5]	40.85	92.42	11.42
p-cluster215005	TMW64953.1	hypothetical protein Poli38472_009120 [Pythium oligandrum]	58.39	89.51	14.27
p-cluster215696	CAF1092469.1	unnamed protein product [Didymodactylos carnosus]	35.42	35.76	91.00
p-cluster216037	TMW67722.1	hypothetical protein Poli38472_007394 [Pythium oligandrum]	34.30	57.71	42.95
p-cluster216082	TMW59707.1	hypothetical protein Poli38472_004776 [Pythium oligandrum]	25.06	75.72	69.43
p-cluster216133	TMW55321.1	hypothetical protein Poli38472_013212 [Pythium oligandrum]	50.12	92.99	81.97
p-cluster216391	TMW66051.1	hypothetical protein Poli38472_003816 [Pythium oligandrum]	52.05	39.57	55.42
p-cluster216745	TMW65750.1	hypothetical protein Poli38472_008392 [Pythium oligandrum]	60.45	79.91	87.50
p-cluster216772	GAX93845.1	thioredoxin-like protein, putative [Pythium insidiosum]	98.33	18.35	30.93
p-cluster216792	GAY04474.1	nucleoporin-like protein [Pythium insidiosum]	100.00	100.00	100.00
p-cluster216885	GAY04552.1	ankyrin [Pythium insidiosum]	57.02	81.65	54.16
p-cluster217076	KAF4030060.1	hypothetical protein GN244_ATG18147 [Phytophthora infestans]	94.12	100.00	45.21
p-cluster217493	KAI3654286.1	hypothetical protein MP228_001005 [Amoeboaphelidium protococcarum]	45.61	27.27	26.89
p-cluster217501	GAY05172.1	cystathionine beta-synthase [Pythium insidiosum]	100.00	100.00	100.00
p-cluster217705	GAY05379.1	mucin-21, partial [Pythium insidiosum]	100.00	100.00	100.00
p-cluster217794	GAY05468.1	Myblike DNAbinding domain containing protein putativ [Pythium insidiosum]	100.00	100.00	100.00
p-cluster217846	GAX95883.1	pepsin-like aspartic protease A1 [Pythium insidiosum]	61.80	67.96	99.77
p-cluster218100	GAY03537.1	zinc transporter, putative [Pythium insidiosum]	98.92	28.70	19.25
p-cluster218139	ETP49795.1	hypothetical protein, variant 8 [Phytophthora parasitica P10297]	41.59	87.50	32.38
p-cluster218429	KAF4041413.1	Roadblock/LC7 domain [Phytophthora infestans]	62.81	29.16	86.51
p-cluster218544	TMW59482.1	hypothetical protein Poli38472_004551 [Pythium oligandrum]	46.58	98.67	15.41
p-cluster218669	TMW69261.1	hypothetical protein Poli38472_001417 [Pythium oligandrum]	51.15	79.88	29.57
p-cluster218727	GAX97986.1	putative membrane protein [Pythium insidiosum]	100.00	100.00	100.00
p-cluster218838	KAF1330383.1	hypothetical protein FI667_g5117, partial [Globisporangium splendens]	50.39	100.00	6.42
p-cluster218863	RLN96816.1	hypothetical protein BBJ28_00012064 [Nothophytophthora sp. Chile5]	35.92	98.96	3.38
p-cluster218935	TMW61209.1	hypothetical protein Poli38472_013672 [Pythium oligandrum]	29.38	74.13	25.52
p-cluster219169	TMW56750.1	hypothetical protein Poli38472_006760 [Pythium oligandrum]	50.00	51.97	4.10
p-cluster219352	KAH7468750.1	hypothetical protein KRP23_11126 [Phytophthora ramorum]	56.92	78.31	51.59
p-cluster219357	TMW56261.1	hypothetical protein Poli38472_008909 [Pythium oligandrum]	43.96	81.98	20.54
p-cluster219554	KAG6576592.1	rho gtpase activating protein 2 [Phytophthora cinnamomi]	46.24	41.10	18.45
p-cluster219591	GAX95356.1	CUG-BP- and ETR-3-like factor, putative [Pythium insidiosum]	76.47	87.50	19.54
p-cluster219755	TMW57286.1	hypothetical protein Poli38472_003211 [Pythium oligandrum]	51.85	90.00	16.35
p-cluster219914	TMW58704.1	hypothetical protein Poli38472_010263 [Pythium oligandrum]	56.62	76.79	38.02
p-cluster220007	RLN61831.1	hypothetical protein BBP00_00005153 [Phytophthora kernoviae]	57.55	88.24	8.91
p-cluster220156	GAX98858.1	peptidyl-prolyl cis-trans isomerase D [Pythium insidiosum]	87.11	56.03	96.52
p-cluster220205	TMW66386.1	hypothetical protein Poli38472_004151 [Pythium oligandrum]	35.50	61.25	49.21
p-cluster220386	TMW63531.1	hypothetical protein Poli38472_002472 [Pythium oligandrum]	35.86	78.98	25.04
p-cluster220416	KAG3119242.1	hypothetical protein PI125_g2181 [Phytophthora idaei]	43.44	53.48	54.91
p-cluster220625	TMW66084.1	hypothetical protein Poli38472_003849 [Pythium oligandrum]	42.71	91.00	20.67
p-cluster220770	TMW59710.1	hypothetical protein Poli38472_004779 [Pythium oligandrum]	53.13	40.67	93.77
p-cluster220885	ETK72756.1	hypothetical protein, variant [Phytophthora parasitica]	39.39	54.08	36.30

p-cluster221015	GAY03849.1	transmembrane protein, putative [Pythium insidiosum]	74.76	27.84	11.56
p-cluster221088	TMW56348.1	hypothetical protein Poli38472_006358 [Pythium oligandrum]	58.33	54.62	13.35
p-cluster221106	GAY00566.1	DEAD/DEAH box RNA helicase, putative [Pythium insidiosum]	80.75	100.00	21.11
p-cluster221119	TMW67307.1	hypothetical protein Poli38472_012423 [Pythium oligandrum]	35.85	33.89	31.06
p-cluster221149	TMW63627.1	hypothetical protein Poli38472_002568 [Pythium oligandrum]	64.38	50.00	8.34
p-cluster221293	TMW64642.1	hypothetical protein Poli38472_011522 [Pythium oligandrum]	48.11	28.16	52.36
p-cluster221515	TMW59271.1	hypothetical protein Poli38472_004340 [Pythium oligandrum]	30.02	78.69	33.77
p-cluster221694	TMW67953.1	hypothetical protein Poli38472_007625 [Pythium oligandrum]	52.24	42.61	16.82
p-cluster221713	GAX93818.1	Basophilic leukemia-expressed protein Bles03 [Pythium insidiosum]	81.52	78.88	100.00
p-cluster221967	TMW65117.1	hypothetical protein Poli38472_009284 [Pythium oligandrum]	33.33	78.77	29.52
p-cluster222004	GAY04439.1	multidrug resistance protein 1-like protein [Pythium insidiosum]	97.30	35.60	46.50
p-cluster222199	TMW64742.1	hypothetical protein Poli38472_011622 [Pythium oligandrum]	44.23	57.99	10.35
p-cluster222499	TMW58005.1	hypothetical protein Poli38472_013479 [Pythium oligandrum]	40.34	32.57	11.83
p-cluster222543	GAY03954.1	transmembrane protein, putative [Pythium insidiosum]	82.93	44.66	86.97
p-cluster222885	TMW58361.1	hypothetical protein Poli38472_009920 [Pythium oligandrum]	34.93	86.67	36.38
p-cluster223246	OWY94159.1	hypothetical protein PHMEG_00036190, partial [Phytophthora megakarya]	38.55	33.71	51.60
p-cluster223304	TMW61862.1	hypothetical protein Poli38472_010925 [Pythium oligandrum]	65.75	51.06	23.08
p-cluster223550	RLN92880.1	hypothetical protein BBJ28_00010072 [Nothophytophthora sp. Chile5]	50.54	73.39	10.13
p-cluster223679	TMW65782.1	hypothetical protein Poli38472_008424 [Pythium oligandrum]	59.42	86.25	16.22
p-cluster224260	UIZ21497.1	hypothetical protein KXD40_000527 [Peronospora effusa]	50.82	79.22	4.01
p-cluster224462	ETL85385.1	hypothetical protein L917_15071, partial [Phytophthora parasitica]	29.60	60.43	64.83
p-cluster224463	TMW68261.1	hypothetical protein Poli38472_005729 [Pythium oligandrum]	100.00	13.04	8.54
p-cluster224536	TMW57910.1	hypothetical protein Poli38472_013384 [Pythium oligandrum]	41.11	84.91	20.77
p-cluster224777	TMW60106.1	hypothetical protein Poli38472_000148 [Pythium oligandrum]	56.94	79.76	11.73
p-cluster224968	GAY02183.1	inositol polyphosphate 5phosphatase putative [Pythium insidiosum]	100.00	68.16	98.56
p-cluster225305	RLN94751.1	hypothetical protein BBJ28_00014395 [Nothophytophthora sp. Chile5]	37.70	60.30	28.61
p-cluster225530	GAY03270.1	proteasomal ubiquitin receptor ADRM1like protein put [Pythium insidiosum]	91.94	86.71	24.60
p-cluster225604	RLN45516.1	hypothetical protein BBJ28_00021378 [Nothophytophthora sp. Chile5]	32.93	30.66	32.72
p-cluster226302	KAG1710562.1	hypothetical protein DVH05_013288 [Phytophthora capsici]	35.97	99.17	44.27
p-cluster226627	GAY04708.1	TKL protein kinase [Pythium insidiosum]	36.36	45.64	17.01
p-cluster226791	GAY05005.1	M96 mating-specific protein family [Pythium insidiosum]	98.58	15.10	64.98
p-cluster226857	TMW69597.1	hypothetical protein Poli38472_001753 [Pythium oligandrum]	27.11	62.85	79.12
p-cluster227261	GAY06546.1	transmembrane protein, putative, partial [Pythium insidiosum]	94.83	45.67	4.15
p-cluster227359	TMW55697.1	hypothetical protein Poli38472_010579 [Pythium oligandrum]	36.81	94.83	24.41
p-cluster227399	KAE9034024.1	hypothetical protein PR002_g8356 [Phytophthora rubi]	62.50	39.85	76.03
p-cluster227533	TMW56240.1	hypothetical protein Poli38472_008888 [Pythium oligandrum]	37.73	93.19	20.84
p-cluster228300	RHY68101.1	hypothetical protein DYB30_013809, partial [Aphanomyces astaci]	36.68	88.24	72.59
p-cluster228567	TMW55070.1	hypothetical protein Poli38472_013832 [Pythium oligandrum]	56.00	43.10	4.62
p-cluster228775	TMW63506.1	hypothetical protein Poli38472_002447 [Pythium oligandrum]	55.23	32.01	57.17
p-cluster228823	TMW68151.1	hypothetical protein Poli38472_007823 [Pythium oligandrum]	35.31	93.92	34.81
p-cluster228964	GAX92812.1	integrator complex subunit, putative [Pythium insidiosum]	85.26	97.94	9.52
p-cluster229097	RLN89474.1	hypothetical protein BBJ28_00021748 [Nothophytophthora sp. Chile5]	54.37	67.32	17.08
p-cluster229306	TMW65289.1	hypothetical protein Poli38472_007931 [Pythium oligandrum]	26.29	85.88	28.45
p-cluster229407	TMW68695.1	hypothetical protein Poli38472_006163 [Pythium oligandrum]	44.86	100.00	15.67
p-cluster229487	NQW04709.1	hypothetical protein [Acidobacteria bacterium]	48.64	40.15	83.97
p-cluster229548	RLN10179.1	hypothetical protein BBJ28_00007240 [Nothophytophthora sp. Chile5]	31.48	41.37	14.74
p-cluster229591	TMW69413.1	hypothetical protein Poli38472_001569 [Pythium oligandrum]	43.88	95.65	30.02
p-cluster229630	RHZ04960.1	hypothetical protein DYB31_004227, partial [Aphanomyces astaci]	36.89	44.97	81.38
p-cluster229658	TMW61534.1	hypothetical protein Poli38472_012725 [Pythium oligandrum]	79.66	27.31	12.94
p-cluster230104	TMW57635.1	hypothetical protein Poli38472_003560 [Pythium oligandrum]	44.60	95.21	24.38
p-cluster230163	GAY02456.1	AGC protein kinase [Pythium insidiosum]	98.72	22.54	5.04
p-cluster230253	TMW60992.1	hypothetical protein Poli38472_014453 [Pythium oligandrum]	92.50	23.26	2.89
p-cluster230348	TMW55752.1	hypothetical protein Poli38472_010634 [Pythium oligandrum]	66.67	40.70	12.99
p-cluster230463	POM68463.1	Hypothetical protein PHPALM_15376 [Phytophthora palmivora var. palmivora]	40.61	50.99	65.51
p-cluster230498	TMW63447.1	hypothetical protein Poli38472_002388 [Pythium oligandrum]	70.45	21.67	10.95
p-cluster230517	TMW65279.1	hypothetical protein Poli38472_007921 [Pythium oligandrum]	39.78	91.63	17.82
p-cluster230530	KAF1330427.1	Myosin-like protein, partial [Globisporangium splendens]	82.72	28.13	3.88
p-cluster230780	TMW69126.1	hypothetical protein Poli38472_001282 [Pythium oligandrum]	39.90	57.41	16.31
p-cluster230895	KAF0734564.1	hypothetical protein Ae201684_008806 [Aphanomyces euteiches]	40.51	56.12	24.68
p-cluster230938	RLN72494.1	hypothetical protein BBJ28_00004551 [Nothophytophthora sp. Chile5]	50.45	100.00	8.94
p-cluster231055	TMW55711.1	hypothetical protein Poli38472_010593 [Pythium oligandrum]	48.68	54.92	10.20
p-cluster231159	GAX98015.1	pantothenate kinase [Pythium insidiosum]	100.00	43.27	30.13

p-cluster231268	XP_008910552.1	hypothetical protein PPTG_23659 [Phytophthora parasitica INRA-310]	87.30	82.89	92.65
p-cluster231297	ETP54764.1	hypothetical protein F442_00610 [Phytophthora parasitica P10297]	37.70	55.86	20.66
p-cluster231298	TMW60820.1	hypothetical protein Poli38472_000862 [Pythium oligandrum]	75.20	47.35	21.66
p-cluster231483	TMW62077.1	hypothetical protein Poli38472_009570 [Pythium oligandrum]	52.31	82.28	10.15
p-cluster231917	GAY00382.1	vtype hatpase putative [Pythium insidiosum]	96.97	98.51	9.01
p-cluster232054	TMW65391.1	hypothetical protein Poli38472_008033 [Pythium oligandrum]	42.95	62.07	20.16
p-cluster232157	KAE8893540.1	hypothetical protein PF003_g22535 [Phytophthora fragariae]	49.28	38.42	7.44
p-cluster232161	GAY02111.1	elicitor-like transglutaminase [Pythium insidiosum]	36.94	48.40	15.09
p-cluster232321	KAE9351580.1	hypothetical protein PR003_g4815 [Phytophthora rubi]	29.05	98.50	12.89
p-cluster232544	GAY01427.1	polycomb-like protein [Pythium insidiosum]	96.51	100.00	10.18
p-cluster232741	KUF98440.1	hypothetical protein AM588_10011501 [Phytophthora nicotianae]	75.56	100.00	58.44
p-cluster232775	RHZ04960.1	hypothetical protein DYB31_004227, partial [Aphanomyces astaci]	42.55	77.05	37.25
p-cluster232874	TMW57498.1	hypothetical protein Poli38472_003423 [Pythium oligandrum]	55.93	62.77	21.93
p-cluster232888	TMW65934.1	hypothetical protein Poli38472_003699 [Pythium oligandrum]	35.77	99.21	13.37
p-cluster232897	GAY01996.1	peroxisomal multifunctional enzyme, putative [Pythium insidiosum]	100.00	68.81	7.34
p-cluster233045	GAX95191.1	glycosylltransferase-like domain-containing protein 1-like protein [Pythium insidiosum]	94.44	100.00	3.75
p-cluster233075	GAX97205.1	polycomb-like protein [Pythium insidiosum]	100.00	100.00	10.13
p-cluster233076	TMW67471.1	hypothetical protein Poli38472_011091 [Pythium oligandrum]	34.86	100.00	29.85
p-cluster233211	KAF1317592.1	Dead/deah box RNA helicase, partial [Globisporangium splendens]	44.79	87.96	6.50
p-cluster233316	KAE9294806.1	hypothetical protein PF008_g24441 [Phytophthora fragariae]	37.20	21.84	39.13
p-cluster233538	KAF1334511.1	Cmgc/mapk protein kinase, partial [Globisporangium splendens]	75.08	31.00	41.75
p-cluster233784	GAY00460.1	serine protease family S33, putative [Pythium insidiosum]	96.45	70.78	52.19
p-cluster233915	GAX99781.1	2oxoglutarate and iron-dependent oxygenase domain containing 1, putative [Pythium insidiosum]	97.42	52.66	100.00
p-cluster233975	GAX92883.1	oxidoreductase [Pythium insidiosum]	95.01	18.34	97.07
p-cluster233985	GAY02539.1	radial spoke protein [Pythium insidiosum]	97.04	32.72	98.90
p-cluster234218	KAF1334268.1	hypothetical protein FI667_g2339, partial [Globisporangium splendens]	37.42	98.98	53.52
p-cluster234360	TMW60015.1	hypothetical protein Poli38472_000057 [Pythium oligandrum]	28.53	99.03	42.30
p-cluster234411	TMW55163.1	hypothetical protein Poli38472_013925 [Pythium oligandrum]	51.55	59.51	18.59
p-cluster234464	GAX95191.1	glycosylltransferase-like domain-containing protein 1-like protein [Pythium insidiosum]	37.60	94.94	41.32
p-cluster234720	KUF91150.1	hypothetical protein AM588_10005102 [Phytophthora nicotianae]	37.66	25.96	87.43
p-cluster234829	TMW67146.1	hypothetical protein Poli38472_012262 [Pythium oligandrum]	52.24	55.56	14.89
p-cluster235016	TMW67327.1	hypothetical protein Poli38472_012443 [Pythium oligandrum]	76.92	18.76	66.89
p-cluster235031	TMW61447.1	hypothetical protein Poli38472_012638 [Pythium oligandrum]	52.08	17.02	13.33
p-cluster235269	TMW60632.1	hypothetical protein Poli38472_000674 [Pythium oligandrum]	44.39	62.29	63.41
p-cluster235279	TMW60366.1	hypothetical protein Poli38472_000408 [Pythium oligandrum]	52.54	36.73	40.00
p-cluster235356	RLN93308.1	hypothetical protein BBJ28_00002099 [Nothophytophthora sp. Chile5]	33.22	99.44	58.48
p-cluster235403	KAF1322418.1	hypothetical protein FI667_g11305, partial [Globisporangium splendens]	76.79	13.93	7.04
p-cluster235406	TMW64191.1	hypothetical protein Poli38472_012813 [Pythium oligandrum]	32.67	89.62	51.39
p-cluster235462	ETM42995.1	hypothetical protein L914_11448 [Phytophthora parasitica]	38.26	98.65	30.17
p-cluster235648	KAF1332558.1	DNA-binding protein, partial [Globisporangium splendens]	74.39	46.65	78.60
p-cluster235676	TMW57619.1	hypothetical protein Poli38472_003544 [Pythium oligandrum]	32.62	94.93	87.12
p-cluster235696	XP_002902977.1	conserved hypothetical protein [Phytophthora infestans T30-4]	41.18	81.12	51.83
p-cluster235704	GAY03502.1	thymidylate synthase [Pythium insidiosum]	86.55	33.45	100.00
p-cluster235865	GAX98339.1	folic acid synthesis protein fol1, putative [Pythium insidiosum]	99.05	16.33	13.46
p-cluster235945	TMW63758.1	hypothetical protein Poli38472_002699 [Pythium oligandrum]	52.73	30.92	17.68
p-cluster236024	TMW61862.1	hypothetical protein Poli38472_010925 [Pythium oligandrum]	65.75	24.41	23.08
p-cluster236256	GAX93503.1	sister chromatid cohesion protein DCC1, putative [Pythium insidiosum]	98.33	32.91	53.89
p-cluster236267	TMW65535.1	hypothetical protein Poli38472_008177 [Pythium oligandrum]	34.85	95.61	50.35
p-cluster236321	KUF95642.1	hypothetical protein AM587_10013825 [Phytophthora nicotianae]	36.64	65.98	54.83
p-cluster236542	GAY06457.1	hydroxyacyl-coenzyme A dehydrogenase, mitochondrial precursor [Pythium insidiosum]	71.29	73.55	19.20
p-cluster236600	XP_009523264.1	hypothetical protein PHYSODRAFT_328648 [Phytophthora sojae]	49.33	55.15	7.11
p-cluster236845	XP_024579170.1	tubby-like protein [Plasmopara halstedii]CEG42801.1 tubby-like protein [Plasmopara halstedii]	78.57	51.22	7.07
p-cluster237003	TMW65548.1	hypothetical protein Poli38472_008190 [Pythium oligandrum]	34.46	83.50	37.48
p-cluster237114	TMW59723.1	hypothetical protein Poli38472_004792 [Pythium oligandrum]	43.82	53.46	48.57
p-cluster237638	OQR86084.1	hypothetical protein ACHHYP_10996 [Achlya hypogyna]	54.11	90.55	17.45
p-cluster237651	RLO02166.1	hypothetical protein DYB28_011910, partial [Aphanomyces astaci]	39.82	28.53	69.14
p-cluster238034	TMW66362.1	hypothetical protein Poli38472_004127 [Pythium oligandrum]	35.59	44.33	60.94
p-cluster238309	TMW67416.1	hypothetical protein Poli38472_011036 [Pythium oligandrum]	25.92	83.83	68.02
p-cluster238319	KAG2888480.1	hypothetical protein PC115_g20038, partial [Phytophthora cactorum]	72.22	27.27	19.05
p-cluster238711	GAY01882.1	ser/thr kinase putative [Pythium insidiosum]	93.75	18.64	36.25
p-cluster238816	GAY03794.1	F-box protein, putative [Pythium insidiosum]	97.54	17.97	32.28
p-cluster238864	TMW64298.1	hypothetical protein Poli38472_012920 [Pythium oligandrum]	75.28	34.14	74.86

p-cluster239331	TMW64441.1	hypothetical protein Poli38472_013063 [Pythium oligandrum]	62.44	65.12	32.53
p-cluster239431	GAX95438.1	LOW QUALITY PROTEIN: paternally-expressed gene 3 protein-like protein [Pythium insidiosum]	95.29	60.98	74.76
p-cluster239806	TMW55309.1	hypothetical protein Poli38472_013200 [Pythium oligandrum]	42.72	39.16	50.47
p-cluster240148	TMW66066.1	hypothetical protein Poli38472_003831 [Pythium oligandrum]	45.83	48.25	51.54
p-cluster240373	TMW58349.1	hypothetical protein Poli38472_009908 [Pythium oligandrum]	56.33	31.34	20.00
p-cluster240592	GAY03738.1	AGC protein kinase, partial [Pythium insidiosum]	90.35	100.00	44.49
p-cluster240664	GAY02043.1	protein CREG1 precursor [Pythium insidiosum]	92.46	54.35	92.28
p-cluster240905	TMW58777.1	hypothetical protein Poli38472_006922 [Pythium oligandrum]	57.59	100.00	15.08
p-cluster241238	GAX96046.1	biotin synthase [Pythium insidiosum]	99.73	46.41	20.40
p-cluster241477	GAX92552.1	protease inhibitor EpiC4 [Pythium insidiosum]	33.62	94.12	47.09
p-cluster241634	KAG6621911.1	metalloprotease family [Phytophthora cinnamomi]	49.38	39.54	93.17
p-cluster241689	TMW56094.1	hypothetical protein Poli38472_008742 [Pythium oligandrum]	63.89	36.76	37.22
p-cluster241841	TMW56336.1	hypothetical protein Poli38472_006346 [Pythium oligandrum]	38.94	41.36	12.25
p-cluster241926	GAY02998.1	COP9 signalosome complex subunit, putative [Pythium insidiosum]	98.80	45.96	27.57
p-cluster241955	TMW58040.1	hypothetical protein Poli38472_013514 [Pythium oligandrum]	30.43	100.00	18.04
p-cluster241956	TMW58041.1	hypothetical protein Poli38472_013515 [Pythium oligandrum]	60.36	33.41	97.92
p-cluster242271	KAF0695726.1	hypothetical protein As57867_013412 [Aphanomyces stellatus]	28.26	98.10	14.05
p-cluster242625	GAY06700.1	replication protein [Pythium insidiosum]	98.21	80.71	92.49
p-cluster242781	CAF9914021.1	hypothetical protein ALECFALPRED_009209 [Alectoria fallacina]	29.19	28.12	30.26
p-cluster242886	KAF0730381.1	hypothetical protein Ae201684_012374 [Aphanomyces euteiches]	40.65	93.60	76.28
p-cluster242890	RLN86357.1	hypothetical protein BBJ28_00004565 [Nothophytophthora sp. Chile5]	31.74	36.68	32.19
p-cluster242942	XP_009518349.1	hypothetical protein PHYSODRAFT_324321 [Phytophthora sojae]	32.42	43.51	49.81
p-cluster243028	TMW59513.1	hypothetical protein Poli38472_004582 [Pythium oligandrum]	62.57	27.76	57.30
p-cluster243157	XP_009845798.1	hypothetical protein H257_18442 [Aphanomyces astaci]	29.94	37.84	83.85
p-cluster243274	TMW57697.1	hypothetical protein Poli38472_014300 [Pythium oligandrum]	44.86	53.93	4.46
p-cluster243278	ETO83702.1	hypothetical protein F444_02319 [Phytophthora parasitica P1976]	64.10	14.13	15.92
p-cluster243546	TMW59325.1	hypothetical protein Poli38472_004394 [Pythium oligandrum]	48.80	90.71	17.91
p-cluster243767	TMW66477.1	hypothetical protein Poli38472_004242 [Pythium oligandrum]	43.02	88.59	14.00
p-cluster243819	TMW67670.1	hypothetical protein Poli38472_011290 [Pythium oligandrum]	53.95	47.50	15.58
p-cluster243971	XP_009845798.1	hypothetical protein H257_18442 [Aphanomyces astaci]	32.14	39.38	86.98
p-cluster244264	TMW56237.1	hypothetical protein Poli38472_008885 [Pythium oligandrum]	23.60	96.79	28.55
p-cluster244370	TMW68011.1	hypothetical protein Poli38472_007683 [Pythium oligandrum]	28.49	81.08	46.90
p-cluster244418	TMW64732.1	hypothetical protein Poli38472_011612 [Pythium oligandrum]	25.61	72.14	14.37
p-cluster245001	TMW66487.1	hypothetical protein Poli38472_004252 [Pythium oligandrum]	33.53	78.28	33.54
p-cluster245026	KAF1327813.1	Ubiquinol cytochrome c reductase subunit qcr9, partial [Globisporangium splendens]	60.53	34.23	74.49
p-cluster245102	XP_002895278.1	putative E3 ubiquitin-protein ligase MARCH6 [Phytophthora infestans T30-4]	62.39	48.44	8.47
p-cluster245147	TMW66487.1	hypothetical protein Poli38472_004252 [Pythium oligandrum]	45.02	90.52	21.47
p-cluster245167	TMW66819.1	hypothetical protein Poli38472_011935 [Pythium oligandrum]	46.67	71.33	5.28
p-cluster245357	RLN70580.1	hypothetical protein BBJ28_00017399 [Nothophytophthora sp. Chile5]	35.92	54.44	30.73
p-cluster245535	TMW69170.1	hypothetical protein Poli38472_001326 [Pythium oligandrum]	42.86	70.61	17.03
p-cluster245610	RLN72535.1	hypothetical protein BBJ28_00017053 [Nothophytophthora sp. Chile5]	33.48	87.45	52.71
p-cluster245695	KAE8901877.1	hypothetical protein PF003_g13847 [Phytophthora fragariae]	42.11	59.72	68.91
p-cluster245781	TMW69170.1	hypothetical protein Poli38472_001326 [Pythium oligandrum]	60.89	96.22	14.26
p-cluster245832	TMW69518.1	hypothetical protein Poli38472_001674 [Pythium oligandrum]	40.59	85.09	28.83
p-cluster246066	TMW66169.1	hypothetical protein Poli38472_003934 [Pythium oligandrum]	35.04	66.86	6.45
p-cluster246172	TMW55894.1	hypothetical protein Poli38472_008542 [Pythium oligandrum]	52.33	100.00	5.27
p-cluster246478	GAX94240.1	ser/thr kinase [Pythium insidiosum]	98.91	100.00	8.14
p-cluster246548	TMW59163.1	hypothetical protein Poli38472_007308 [Pythium oligandrum]	58.59	36.03	99.41
p-cluster246673	TMW64264.1	hypothetical protein Poli38472_012886 [Pythium oligandrum]	63.89	33.03	11.73
p-cluster246768	TMW67809.1	hypothetical protein Poli38472_007481 [Pythium oligandrum]	64.63	20.97	11.31
p-cluster246899	KAE9338728.1	hypothetical protein PF008_g11932 [Phytophthora fragariae]	27.73	86.71	71.91
p-cluster246924	TMW63743.1	hypothetical protein Poli38472_002684 [Pythium oligandrum]	45.74	85.86	19.90
p-cluster247018	GAX93117.1	40S ribosomal protein S15 [Pythium insidiosum]	100.00	29.40	75.97
p-cluster247097	RLN70580.1	hypothetical protein BBJ28_00017399 [Nothophytophthora sp. Chile5]	34.42	13.12	33.57
p-cluster247166	GAY03096.1	glutamate-tRNA ligase [Pythium insidiosum]	98.97	40.59	10.84
p-cluster247250	TMW60682.1	hypothetical protein Poli38472_000724 [Pythium oligandrum]	42.38	44.93	77.92
p-cluster247308	TMW63204.1	hypothetical protein Poli38472_002145 [Pythium oligandrum]	57.75	46.90	10.00
p-cluster247323	TMW59125.1	hypothetical protein Poli38472_007270 [Pythium oligandrum]	40.85	34.10	71.53
p-cluster247535	XP_009536759.1	hypothetical protein PHYSODRAFT_306431 [Phytophthora sojae]	40.64	28.93	32.85
p-cluster247949	GAX96550.1	protein kinase putative [Pythium insidiosum]	81.98	41.61	83.66
p-cluster248202	TMW65756.1	hypothetical protein Poli38472_008398 [Pythium oligandrum]	36.75	92.51	50.39
p-cluster248460	KAF1329430.1	hypothetical protein FI667_g6071, partial [Globisporangium splendens]	52.11	23.39	37.77

p-cluster248690	KAG3102054.1	hypothetical protein PI125_g14292 [Phytophthora idaei]	47.79	50.19	31.12
p-cluster248844	TMW68722.1	hypothetical protein Poli38472_006190 [Pythium oligandrum]	39.24	89.71	24.51
p-cluster249043	GAY05523.1	DNA polymerase epsilon subunit, putative [Pythium insidiosum]	72.44	36.15	28.72
p-cluster249109	TMW64771.1	hypothetical protein Poli38472_011651 [Pythium oligandrum]	57.16	43.30	99.66
p-cluster249178	TMW59500.1	hypothetical protein Poli38472_004569 [Pythium oligandrum]	70.43	43.89	11.83
p-cluster249283	RHY82303.1	hypothetical protein DYB35_000732 [Aphanomyces astaci]	59.14	80.17	22.48
p-cluster249388	KAG7376596.1	hypothetical protein PHYBOEH_001465 [Phytophthora boehmeriae]	44.74	41.01	88.89
p-cluster249495	TMW61996.1	hypothetical protein Poli38472_009489 [Pythium oligandrum]	37.42	59.27	60.26
p-cluster249535	KAE9004323.1	hypothetical protein PR001_g17746 [Phytophthora rubi]	36.89	63.58	90.35
p-cluster249649	TMW65212.1	hypothetical protein Poli38472_009379 [Pythium oligandrum]	50.63	19.60	18.99
p-cluster249673	GAX98090.1	isocitrate dehydrogenase, NADP-dependent [Pythium insidiosum]	91.75	81.36	10.02
p-cluster249811	TMW61315.1	hypothetical protein Poli38472_012506 [Pythium oligandrum]	54.29	38.41	70.06
p-cluster249863	KAF1321099.1	hypothetical protein FI667_g12071, partial [Globisporangium splendens]	31.05	29.79	9.24
p-cluster249944	TMW60552.1	hypothetical protein Poli38472_000594 [Pythium oligandrum]	41.31	73.16	54.12
p-cluster250015	TMW65955.1	hypothetical protein Poli38472_003720 [Pythium oligandrum]	69.95	79.20	19.39
p-cluster250206	GAY03411.1	26S proteasome non-ATPase regulatory subunit 6 [Pythium insidiosum]	100.00	40.64	14.06
p-cluster250411	KUF87766.1	hypothetical protein AM587_10001355 [Phytophthora nicotianae]	48.89	37.50	23.20
p-cluster250412	GAX94263.1	TKL protein kinase [Pythium insidiosum]	73.33	100.00	6.28
p-cluster250572	GAY03327.1	enoyl-CoA hydratase/isomerase family, putative [Pythium insidiosum]	75.91	100.00	100.00
p-cluster250652	TMW61927.1	hypothetical protein Poli38472_010990 [Pythium oligandrum]	70.73	87.23	11.58
p-cluster250757	XP_002894984.1	conserved hypothetical protein [Phytophthora infestans T30-4]	41.35	83.61	12.76
p-cluster250863	RLN88384.1	hypothetical protein BBJ28_00006152 [Nothophytophthora sp. Chile5]	35.08	53.20	39.40
p-cluster250930	XP_009845798.1	hypothetical protein H257_18442 [Aphanomyces astaci]	33.33	67.54	72.92
p-cluster251050	TMW62162.1	hypothetical protein Poli38472_009655 [Pythium oligandrum]	35.80	69.12	22.62
p-cluster251153	GAX99784.1	aspartyl protease family A01B, putative [Pythium insidiosum]	75.35	84.27	36.54
p-cluster251247	TMW55845.1	hypothetical protein Poli38472_008493 [Pythium oligandrum]	70.27	31.36	7.25
p-cluster251462	TMW69426.1	hypothetical protein Poli38472_001582 [Pythium oligandrum]	28.33	74.01	46.73
p-cluster251467	TMW67612.1	hypothetical protein Poli38472_011232 [Pythium oligandrum]	45.36	22.51	26.44
p-cluster251506	TMW69785.1	hypothetical protein Poli38472_001941 [Pythium oligandrum]	35.71	41.20	54.81
p-cluster251626	TMW62304.1	hypothetical protein Poli38472_009797 [Pythium oligandrum]	43.63	52.80	18.62
p-cluster251687	GAX96604.1	E3 ubiquitin ligase, putative [Pythium insidiosum]	78.46	51.85	30.02
p-cluster251734	TMW63714.1	hypothetical protein Poli38472_002655 [Pythium oligandrum]	28.84	84.71	16.83
p-cluster251869	OQS01806.1	hypothetical protein THRCLA_21607, partial [Thraustotheca clavata]	28.33	70.98	58.85
p-cluster251876	KAF1333590.1	hypothetical protein FI667_g2426, partial [Globisporangium splendens]	47.75	73.76	20.04
p-cluster252120	TMW59917.1	hypothetical protein Poli38472_004986 [Pythium oligandrum]	42.47	94.70	28.86
p-cluster252265	GAX96429.1	exosome complex exonuclease RRP41-like protein [Pythium insidiosum]	96.26	27.72	75.71
p-cluster252310	TMW57773.1	hypothetical protein Poli38472_014376 [Pythium oligandrum]	34.66	92.39	98.98
p-cluster252457	TMW66027.1	hypothetical protein Poli38472_003792 [Pythium oligandrum]	37.53	36.42	48.93
p-cluster252491	TMW66324.1	hypothetical protein Poli38472_004089 [Pythium oligandrum]	34.36	76.35	36.27
p-cluster252499	TMW60206.1	hypothetical protein Poli38472_000248 [Pythium oligandrum]	34.93	85.53	50.78
p-cluster252722	KAG6976941.1	hypothetical protein JG688_00000854 [Phytophthora aleatoria]	54.17	62.34	36.92
p-cluster253101	TMW64629.1	hypothetical protein Poli38472_011509 [Pythium oligandrum]	37.43	69.57	19.70
p-cluster253461	GAX92941.1	homoserine dehydrogenase [Pythium insidiosum]	96.26	100.00	29.56
p-cluster253496	TMW60573.1	hypothetical protein Poli38472_000615 [Pythium oligandrum]	54.05	84.34	32.37
p-cluster253504	KAH9121358.1	hypothetical protein LEN26_010709 [Aphanomyces euteiches]	35.90	52.70	37.68
p-cluster253601	GAX92391.1	myb-like DNA-binding protein, putative [Pythium insidiosum]	100.00	100.00	34.55
p-cluster253602	TMW64595.1	hypothetical protein Poli38472_011475 [Pythium oligandrum]	61.04	38.60	14.38
p-cluster253626	TMW55374.1	hypothetical protein Poli38472_013265 [Pythium oligandrum]	30.63	61.56	38.83
p-cluster253765	GAX92274.1	transmembrane protein, putative [Pythium insidiosum]	94.55	39.43	4.65
p-cluster253881	GAX96405.1	von Willebrand factor A [Pythium insidiosum]	91.33	25.17	49.34
p-cluster253989	TYZ58913.1	hypothetical protein PybrP1_009517, partial [[Pythium] brassicae (nom. inval.)]	55.75	54.59	11.19
p-cluster254031	KAG6611026.1	putative COPII coat assembly protein SEC16 [Phytophthora cinnamomi]	57.64	22.22	14.82
p-cluster254125	TMW68002.1	hypothetical protein Poli38472_007674 [Pythium oligandrum]	37.27	35.26	12.11
p-cluster254183	TMW60247.1	hypothetical protein Poli38472_000289 [Pythium oligandrum]	50.34	46.69	10.26
p-cluster254316	TYZ63996.1	hypothetical protein PybrP1_004231 [[Pythium] brassicae (nom. inval.)]	45.15	80.24	19.34
p-cluster254377	TMW69150.1	hypothetical protein Poli38472_001306 [Pythium oligandrum]	70.00	34.48	24.10
p-cluster254421	CCI48086.1	unnamed protein product [Albugo candida]	29.48	100.00	33.00
p-cluster254465	XP_048542626.1	histone H3.2-like [Triticum urartu]	54.69	39.02	34.68
p-cluster254682	TMW64191.1	hypothetical protein Poli38472_012813 [Pythium oligandrum]	38.33	47.20	15.89
p-cluster254880	KAG6948734.1	hypothetical protein JG687_00015285 [Phytophthora cactorum]	35.05	50.00	29.60
p-cluster254919	TMW59536.1	hypothetical protein Poli38472_004605 [Pythium oligandrum]	47.09	34.44	17.56
p-cluster254991	POM70964.1	Hypothetical protein PHPALM_12537, partial [Phytophthora palmivora var. palmivora]	49.75	36.82	75.00

p-cluster255047	KAF1319629.1	hypothetical protein FI667_g13088, partial [Globisporangium splendens]	66.00	48.54	5.06
p-cluster255172	TMW55680.1	hypothetical protein Poli38472_010562 [Pythium oligandrum]	52.33	75.68	12.56
p-cluster255428	TMW69716.1	hypothetical protein Poli38472_001872 [Pythium oligandrum]	48.99	83.33	91.35
p-cluster255434	TMW66196.1	hypothetical protein Poli38472_003961 [Pythium oligandrum]	33.49	54.99	59.13
p-cluster255469	TMW55025.1	hypothetical protein Poli38472_013787 [Pythium oligandrum]	49.42	83.09	34.06
p-cluster255580	TMW56648.1	hypothetical protein Poli38472_006658 [Pythium oligandrum]	33.66	77.39	20.76
p-cluster255638	TMW68706.1	hypothetical protein Poli38472_006174 [Pythium oligandrum]	39.56	100.00	100.00
p-cluster255720	TMW60229.1	hypothetical protein Poli38472_000271 [Pythium oligandrum]	26.34	95.75	13.68
p-cluster255945	RLN48993.1	hypothetical protein BBJ28_00012922, partial [Nothophytophthora sp. Chile5]	29.95	32.76	19.02
p-cluster255992	GAY04303.1	Intraflagellar Transport Protein 57 [Pythium insidiosum]	55.97	51.61	14.42
p-cluster256054	TMW64371.1	hypothetical protein Poli38472_012993 [Pythium oligandrum]	51.28	45.69	83.69
p-cluster256229	TMW63249.1	hypothetical protein Poli38472_002190 [Pythium oligandrum]	33.03	83.01	32.81
p-cluster256443	CCI11391.1	unnamed protein product [Albugo candida]	35.46	96.58	34.53
p-cluster256656	TMW61549.1	hypothetical protein Poli38472_012740 [Pythium oligandrum]	59.14	49.06	23.61
p-cluster256792	TMW57259.1	hypothetical protein Poli38472_003184 [Pythium oligandrum]	40.82	21.63	16.25
p-cluster256797	TMW62425.1	hypothetical protein Poli38472_005043 [Pythium oligandrum]	36.45	94.42	11.05
p-cluster256823	KAG9407416.1	hypothetical protein AC1031_002143 [Aphanomyces cochlioides]	38.18	78.57	35.97
p-cluster256848	GAY06760.1	G-protein coupled receptor [Pythium insidiosum]	95.00	50.42	11.27
p-cluster257079	GAY03501.1	R2R3 Myb protein [Pythium insidiosum]	98.59	31.28	13.15
p-cluster257084	GAX95438.1	LOW QUALITY PROTEIN: paternally-expressed gene 3 protein-like protein [Pythium insidiosum]	85.11	100.00	31.25
p-cluster257215	TMW59532.1	hypothetical protein Poli38472_004601 [Pythium oligandrum]	43.15	67.47	8.75
p-cluster257227	ETM47369.1	hypothetical protein L914_07907 [Phytophthora parasitica]	37.93	34.80	56.19
p-cluster257259	TMW63930.1	hypothetical protein Poli38472_014635 [Pythium oligandrum]	43.33	48.07	10.76
p-cluster257403	KAG1711761.1	hypothetical protein DVH05_009004 [Phytophthora capsici]	39.58	55.45	8.18
p-cluster257406	TMW68200.1	hypothetical protein Poli38472_007872 [Pythium oligandrum]	24.40	98.03	14.35
p-cluster257721	GAY02090.1	kinesin-like protein [Pythium insidiosum]	96.84	78.39	38.35
p-cluster257771	TMW62787.1	hypothetical protein Poli38472_005405 [Pythium oligandrum]	28.91	97.01	9.29
p-cluster257784	TMW61427.1	hypothetical protein Poli38472_012618 [Pythium oligandrum]	26.10	93.22	36.90
p-cluster257801	TMW63629.1	hypothetical protein Poli38472_002570 [Pythium oligandrum]	33.17	52.24	36.38
p-cluster257815	TMW57116.1	hypothetical protein Poli38472_003041 [Pythium oligandrum]	40.35	94.41	13.37
p-cluster258090	TMW60638.1	hypothetical protein Poli38472_000680 [Pythium oligandrum]	35.46	98.60	4.98
p-cluster258099	TMW61619.1	hypothetical protein Poli38472_010682 [Pythium oligandrum]	43.45	86.31	36.53
p-cluster258135	GAX93878.1	mediator of RNA polymerase II transcription subunit, putative [Pythium insidiosum]	47.50	100.00	51.40
p-cluster258514	TMW68411.1	hypothetical protein Poli38472_005879 [Pythium oligandrum]	62.50	50.26	6.18
p-cluster258558	RLN45971.1	hypothetical protein BBJ28_00019495 [Nothophytophthora sp. Chile5]	31.34	78.82	17.44
p-cluster258605	POM72079.1	DEAD/DEAH box RNA helicase [Phytophthora palmivora var. palmivora]	35.12	93.75	58.40
p-cluster258643	TMW62165.1	hypothetical protein Poli38472_009658 [Pythium oligandrum]	42.86	54.27	4.16
p-cluster258920	KAE9311446.1	hypothetical protein PR003_g20012 [Phytophthora rubi]	46.58	23.64	6.38
p-cluster258991	TMW61747.1	hypothetical protein Poli38472_010810 [Pythium oligandrum]	34.23	55.68	20.79
p-cluster259081	TMW60385.1	hypothetical protein Poli38472_000427 [Pythium oligandrum]	50.00	43.37	17.05
p-cluster259469	KAF1790631.1	Homeodomain-like [Phytophthora cactorum]	37.85	79.80	30.11
p-cluster259530	TMW68861.1	hypothetical protein Poli38472_001017 [Pythium oligandrum]	65.57	44.20	15.21
p-cluster259641	TMW66234.1	hypothetical protein Poli38472_003999 [Pythium oligandrum]	56.00	21.28	2.90
p-cluster259790	TMW56890.1	hypothetical protein Poli38472_002815 [Pythium oligandrum]	63.64	50.30	30.66
p-cluster259912	KAF1321913.1	Type i inositol-3,4-bisphosphate 4-phosphatase, partial [Globisporangium splendens]	41.14	71.96	8.12
p-cluster260004	TMW68415.1	hypothetical protein Poli38472_005883 [Pythium oligandrum]	40.00	100.00	10.07
p-cluster260117	GAY02622.1	DNA topoisomerase VI subunit A [Pythium insidiosum]	96.88	96.97	62.34
p-cluster260372	GAY00029.1	protein SUPPRESSOR OF FRI 4-like isoform X3 [Pythium insidiosum]	75.45	100.00	26.20
p-cluster260503	KAF1322448.1	hypothetical protein FI667_g11173, partial [Globisporangium splendens]	54.64	65.00	6.21
p-cluster260779	TMW68757.1	hypothetical protein Poli38472_006225 [Pythium oligandrum]	42.86	88.35	41.36
p-cluster260880	TMW69361.1	hypothetical protein Poli38472_001517 [Pythium oligandrum]	57.69	52.53	6.90
p-cluster261032	TMW66325.1	hypothetical protein Poli38472_004090 [Pythium oligandrum]	89.74	35.14	2.98
p-cluster261048	GAX95438.1	LOW QUALITY PROTEIN: paternally-expressed gene 3 protein-like protein [Pythium insidiosum]	95.83	100.00	11.69
p-cluster261182	TMW64839.1	hypothetical protein Poli38472_009006 [Pythium oligandrum]	40.63	98.39	28.44
p-cluster261200	GAX97356.1	dynamin [Pythium insidiosum]	95.28	100.00	14.32
p-cluster261266	GAY02450.1	AGC protein kinase [Pythium insidiosum]	99.24	100.00	13.52
p-cluster261333	GAX93395.1	DNA polymerase subunit alpha B, putative, partial [Pythium insidiosum]	98.21	82.35	5.15
p-cluster261432	KAG3145052.1	hypothetical protein PC128_g24283 [Phytophthora cactorum]	40.28	80.68	69.23
p-cluster261437	GAY04853.1	26S proteasome regulatory subunit N1 [Pythium insidiosum]	100.00	100.00	3.17
p-cluster261439	TMW64383.1	hypothetical protein Poli38472_013005 [Pythium oligandrum]	36.67	77.59	3.93
p-cluster261495	TMW57812.1	hypothetical protein Poli38472_014415 [Pythium oligandrum]	46.91	86.96	21.79
p-cluster261661	TMW63422.1	hypothetical protein Poli38472_002363 [Pythium oligandrum]	50.00	57.14	15.75

p-cluster261968	TMW68627.1	hypothetical protein Poli38472_006095 [Pythium oligandrum]	94.44	31.21	34.84
p-cluster262074	TMW63047.1	hypothetical protein Poli38472_005665 [Pythium oligandrum]	41.64	98.33	6.38
p-cluster262638	TMW55396.1	hypothetical protein Poli38472_013287 [Pythium oligandrum]	66.01	43.48	64.50
p-cluster262716	KAG3120136.1	hypothetical protein PI125_g1390 [Phytophthora idaei]	25.07	47.00	39.93
p-cluster262986	TMW68462.1	hypothetical protein Poli38472_005930 [Pythium oligandrum]	58.33	40.65	97.76
p-cluster263223	TMW62656.1	hypothetical protein Poli38472_005274 [Pythium oligandrum]	88.89	25.86	40.91
p-cluster263259	TMW64635.1	hypothetical protein Poli38472_011515 [Pythium oligandrum]	40.72	100.00	46.78
p-cluster263496	TMW60634.1	hypothetical protein Poli38472_000676 [Pythium oligandrum]	46.63	38.78	28.28
p-cluster263549	TMW58491.1	hypothetical protein Poli38472_010050 [Pythium oligandrum]	42.55	82.14	22.54
p-cluster263687	GAY00122.1	kinesin-like protein [Pythium insidiosum]	92.14	67.95	56.83
p-cluster263688	TMW56133.1	hypothetical protein Poli38472_008781 [Pythium oligandrum]	41.67	87.74	54.27
p-cluster263707	TMW58075.1	hypothetical protein Poli38472_013549 [Pythium oligandrum]	44.22	45.93	95.51
p-cluster264233	GAY05620.1	glycoprotein elicitor-like protein [Pythium insidiosum]	96.50	16.30	53.68
p-cluster264559	XP_002902461.1	conserved hypothetical protein [Phytophthora infestans T30-4]	41.86	37.28	75.44
p-cluster264599	KAF1328557.1	P-type atpase, partial [Globisporangium splendens]	41.86	66.40	7.64
p-cluster264758	TMW68362.1	hypothetical protein Poli38472_005830 [Pythium oligandrum]	68.18	48.09	24.86
p-cluster265052	GAY04316.1	elongator complex protein putative [Pythium insidiosum]	46.72	65.90	24.13
p-cluster265120	TMW56983.1	hypothetical protein Poli38472_002908 [Pythium oligandrum]	32.80	99.47	31.36
p-cluster265564	TMW59714.1	hypothetical protein Poli38472_004783 [Pythium oligandrum]	42.25	62.15	68.75
p-cluster265663	TMW62636.1	hypothetical protein Poli38472_005254 [Pythium oligandrum]	42.16	47.44	67.57
p-cluster265723	XP_008904825.1	hypothetical protein PPTG_11290 [Phytophthora parasitica INRA-310	38.79	55.75	31.48
p-cluster265786	TMW64834.1	hypothetical protein Poli38472_009001 [Pythium oligandrum]	43.02	70.49	34.62
p-cluster265917	KAF1334147.1	Cyclic nucleotide-binding protein, partial [Globisporangium splendens]	42.79	64.49	43.89
p-cluster266153	RLN52674.1	hypothetical protein BBJ28_00022208 [Nothophytophthora sp. Chile5]	33.09	97.22	39.05
p-cluster266236	TMW55985.1	hypothetical protein Poli38472_008633 [Pythium oligandrum]	91.18	15.81	5.19
p-cluster267040	POM71630.1	Hypothetical protein PHPALM_11777 [Phytophthora palmivora var. palmivora]	55.56	22.50	25.81
p-cluster267049	ETP29174.1	hypothetical protein F442_21640, partial [Phytophthora parasitica P10297]	52.73	21.79	78.47
p-cluster267239	XP_024575210.1	UNCHARACTERIZED [Plasmopara halstedii]CEG38841.1 UNCHARACTERIZED [Plasmopara halstedii]	60.61	50.77	98.70
p-cluster267242	XP_024585799.1	uncharacterized protein PHALS_07194 [Plasmopara halstedii]	50.00	51.25	55.41
p-cluster267555	KAG6610863.1	Tudor-like, plant [Phytophthora cinnamomi]	58.14	39.41	7.18
p-cluster267616	GAX97578.1	GCN5-related N-acetyltransferase [Pythium insidiosum]	93.02	42.05	51.67
p-cluster267623	TMW62813.1	hypothetical protein Poli38472_005431 [Pythium oligandrum]	88.00	32.26	97.09
p-cluster267696	KAG6574742.1	hypothetical protein IUM83_10959 [Phytophthora cinnamomi]	41.94	89.80	34.33
p-cluster267822	GAY00460.1	serine protease family S33, putative [Pythium insidiosum]	74.64	29.44	80.81
p-cluster267846	GAX93078.1	sodium/hydrogen exchanger 3 [Pythium insidiosum]	97.46	97.52	19.31
p-cluster268319	ETI32435.1	hypothetical protein F443_20769 [Phytophthora parasitica P1569]	28.57	72.36	19.19
p-cluster268496	OQS01441.1	Chloride Channel (ClC) Family [Achlya hypogyna]	42.92	42.51	77.34
p-cluster268782	KAF1326940.1	Pantothenate synthetase, partial [Globisporangium splendens]	39.53	36.92	18.77
p-cluster268880	TMW62845.1	hypothetical protein Poli38472_005463 [Pythium oligandrum]	38.76	59.72	34.51
p-cluster268906	TMW58355.1	hypothetical protein Poli38472_009914 [Pythium oligandrum]	74.60	9.40	9.75
p-cluster268925	GAY02632.1	negative elongation factor, putative [Pythium insidiosum]	79.10	87.46	26.56
p-cluster268941	GAX95259.1	CMGC/CDKL protein kinase [Pythium insidiosum]	96.78	100.00	48.67
p-cluster268947	TMW57108.1	hypothetical protein Poli38472_003033 [Pythium oligandrum]	52.21	98.43	46.45
p-cluster269371	GAX98032.1	UDP-glucosyl transferase family protein [Pythium insidiosum]	98.08	22.22	9.90
p-cluster269397	TMW59437.1	hypothetical protein Poli38472_004506 [Pythium oligandrum]	31.86	27.23	17.01
p-cluster269779	KAF1334095.1	putative seven transmembrane receptor-rhodopsin family, partial [Globisporangium splendens]	66.04	94.64	15.87
p-cluster269814	TMW57720.1	hypothetical protein Poli38472_014323 [Pythium oligandrum]	33.49	75.95	32.49
p-cluster270105	GAY01449.1	splicing factor 3 subunit putative [Pythium insidiosum]	97.22	57.14	4.00
p-cluster270526	CCA22524.1	putative polyprotein [Albugo laibachii Nc14]	44.76	54.74	60.00
p-cluster270545	GAY05172.1	cystathionine beta-synthase [Pythium insidiosum]	75.00	37.63	13.22
p-cluster270561	GAX93635.1	histone H1, putative [Pythium insidiosum]	99.42	47.53	100.00
p-cluster270701	KAE9284610.1	hypothetical protein PF008_g27118 [Phytophthora fragariae]	28.77	88.30	41.06
p-cluster270747	GAY05747.1	replication protein [Pythium insidiosum]	33.11	19.34	49.36
p-cluster270971	KAG3172102.1	hypothetical protein PI126_g1577 [Phytophthora idaei]	35.96	66.20	35.43
p-cluster271079	XP_002905231.1	conserved hypothetical protein [Phytophthora infestans T30-4]	53.97	82.89	14.35
p-cluster271414	XP_008911979.1	hypothetical protein PPTG_15517 [Phytophthora parasitica INRA-310]	53.49	14.95	46.37
p-cluster271444	KAF1326725.1	Atp-dependent helicase, partial [Globisporangium splendens]	30.35	85.32	20.75
p-cluster271477	GAY00603.1	serine protease family S33, putative [Pythium insidiosum]	50.62	49.38	7.94
p-cluster271679	KAH7472391.1	ERAD-associated E3 ubiquitin-protein ligase HRD1 [Phytophthora ramorum]	41.98	69.01	22.87
p-cluster271708	TMW55930.1	hypothetical protein Poli38472_008578 [Pythium oligandrum]	28.33	43.07	15.57
p-cluster271864	ETN61562.1	importin alpha [Anopheles darlingi]	30.00	88.96	26.82
p-cluster272055	GAX95022.1	50S ribosomal protein L36e [Pythium insidiosum]	91.18	33.33	31.78

p-cluster272079	RLN90587.1	hypothetical protein BBJ28_00021737 [Nothophytophthora sp. Chile5]	44.74	85.19	34.59
p-cluster272100	KUF84447.1	hypothetical protein AM587_10000297 [Phytophthora nicotianae]	94.39	82.95	30.92
p-cluster272274	TMW69129.1	hypothetical protein Poli38472_001285 [Pythium oligandrum]	65.17	42.69	70.99
p-cluster272320	TMW67430.1	hypothetical protein Poli38472_011050 [Pythium oligandrum]	48.05	64.71	33.64
p-cluster272381	TMW55613.1	hypothetical protein Poli38472_010495 [Pythium oligandrum]	58.71	77.58	43.03
p-cluster272464	TMW62987.1	hypothetical protein Poli38472_005605 [Pythium oligandrum]	52.61	77.91	41.63
p-cluster272472	TMW58632.1	hypothetical protein Poli38472_010191 [Pythium oligandrum]	37.59	26.06	18.92
p-cluster272499	TMW64261.1	hypothetical protein Poli38472_012883 [Pythium oligandrum]	37.81	94.76	54.35
p-cluster272507	TMW59488.1	hypothetical protein Poli38472_004557 [Pythium oligandrum]	46.81	93.74	30.62
p-cluster272759	POM65296.1	Hypothetical protein PHPALM_19013 [Phytophthora palmivora var. palmivora]	55.70	52.32	75.96
p-cluster272803	KAF0725274.1	hypothetical protein Ae201684_016239 [Aphanomyces euteiches]	33.56	60.33	62.03
p-cluster272928	GAX93075.1	protein kinase putative [Pythium insidiosum]	74.16	59.38	9.07
p-cluster273149	GAY03281.1	long-chain-fatty-acid-CoA ligase, putative [Pythium insidiosum]	82.86	96.55	12.99
p-cluster273238	GAY05759.1	RNA-binding protein, putative [Pythium insidiosum]	100.00	100.00	100.00
p-cluster273369	TYZ64244.1	hypothetical protein PybrP1_004416, partial [[Pythium] brassicae (nom. inval.)]	37.37	84.35	9.62
p-cluster273582	GAX96304.1	CRN-like CRN14 [Pythium insidiosum]	40.81	100.00	58.36
p-cluster273595	GAY03285.1	ribosomal protein S18 [Pythium insidiosum]	92.31	93.55	33.33
p-cluster273675	TMW62976.1	hypothetical protein Poli38472_005594 [Pythium oligandrum]	44.67	72.08	14.77
p-cluster273715	KAF1320924.1	Dicer, partial [Globisporangium splendens]	51.65	53.53	4.39
p-cluster273727	OWZ09001.1	LOW QUALITY PROTEIN: reverse transcriptase [Phytophthora megakarya]	32.39	94.35	46.83
p-cluster273819	XP_024583005.1	uncharacterized protein PHALS_03243, partial [Plasmopara halstedii]	59.38	17.34	78.05
p-cluster273885	TMW68236.1	hypothetical protein Poli38472_007908 [Pythium oligandrum]	65.04	35.60	16.60
p-cluster273955	KAF1333759.1	hypothetical protein FI667_g2431, partial [Globisporangium splendens]	60.27	48.99	48.67
p-cluster274066	GAY02111.1	elicitor-like transglutaminase [Pythium insidiosum]	92.97	95.52	17.56
p-cluster274081	RQM14627.1	hypothetical protein DD237_004486 [Peronospora effusa]	35.29	81.48	23.76
p-cluster274106	GAY01326.1	NADH-ubiquinone oxidoreductase, putative [Pythium insidiosum]	100.00	27.14	19.43
p-cluster274129	KAE8963683.1	hypothetical protein PR002_g29213 [Phytophthora rubi]	58.18	55.85	88.71
p-cluster274307	TMW63257.1	hypothetical protein Poli38472_002198 [Pythium oligandrum]	31.51	97.29	35.53
p-cluster274626	TMW64953.1	hypothetical protein Poli38472_009120 [Pythium oligandrum]	32.80	72.22	16.04
p-cluster274801	RLN88384.1	hypothetical protein BBJ28_00006152 [Nothophytophthora sp. Chile5]	38.46	46.76	14.90
p-cluster275076	RYG69685.1	hypothetical protein EON64_02115, partial [archaeon]	34.30	87.61	7.60
p-cluster275096	TMW56555.1	hypothetical protein Poli38472_006565 [Pythium oligandrum]	52.87	77.72	13.47
p-cluster275115	TMW56683.1	hypothetical protein Poli38472_006693 [Pythium oligandrum]	64.10	97.50	6.46
p-cluster275185	TMW57288.1	hypothetical protein Poli38472_003213 [Pythium oligandrum]	35.10	80.77	13.79
p-cluster275195	GAX98736.1	copine-like protein, putative [Pythium insidiosum]	85.71	87.50	2.27
p-cluster275231	GAY01889.1	chromodomain-helicase-DNA-binding protein, putative [Pythium insidiosum]	95.48	100.00	10.95
p-cluster275281	KAF1318776.1	Nucleolar and coiled-body phosphoprotein 1, partial [Globisporangium splendens]	72.88	31.22	11.92
p-cluster275296	TMW57815.1	hypothetical protein Poli38472_014418 [Pythium oligandrum]	51.47	50.81	19.37
p-cluster275369	GAY02247.1	putative glycosyll hydrolase family 5 protein [Pythium insidiosum]	98.57	100.00	9.10
p-cluster275508	GAX98698.1	TKL protein kinase, variant [Pythium insidiosum]	93.55	100.00	2.19
p-cluster275529	TMW56648.1	hypothetical protein Poli38472_006658 [Pythium oligandrum]	51.38	71.01	9.30
p-cluster275971	GAX95883.1	pepsin-like aspartic protease A1 [Pythium insidiosum]	98.68	100.00	17.47
p-cluster275986	GAY06347.1	calcineurin-like phosphoesterase [Pythium insidiosum]	96.36	92.44	9.29
p-cluster276011	TMW65237.1	hypothetical protein Poli38472_009404 [Pythium oligandrum]	53.78	97.48	15.32
p-cluster276014	XP_009518219.1	hypothetical protein PHYSODRAFT_483737 [Phytophthora sojae]	38.73	99.16	5.49
p-cluster276097	TMW63147.1	hypothetical protein Poli38472_002088 [Pythium oligandrum]	59.55	92.63	10.26
p-cluster276152	KAF4139182.1	Autophagocytosis associated protein active-site domain-containing protein [Phytophthora infestans]	38.82	100.00	26.15
p-cluster276200	TMW60730.1	hypothetical protein Poli38472_000772 [Pythium oligandrum]	51.96	33.44	14.23
p-cluster276366	GAX97727.1	Kazal-type serine proteinase inhibitor 1 [Pythium insidiosum]	84.65	42.49	80.52
p-cluster276434	GAX94250.1	phosphatidate cytidylyltransferase, putative, partial [Pythium insidiosum]	70.93	97.65	14.41
p-cluster276639	TMW55411.1	hypothetical protein Poli38472_013302 [Pythium oligandrum]	29.29	99.03	97.14
p-cluster276671	TMW55374.1	hypothetical protein Poli38472_013265 [Pythium oligandrum]	29.27	49.62	58.69
p-cluster276817	GAY06537.1	mitochondrial carrier, partial, partial [Pythium insidiosum]	48.57	78.95	99.34
p-cluster276907	KAF1320406.1	hypothetical protein FI667_g12417, partial [Globisporangium splendens]	44.05	21.56	18.26
p-cluster276983	TMW59745.1	hypothetical protein Poli38472_004814 [Pythium oligandrum]	62.70	43.60	29.58
p-cluster277090	KAG1711309.1	hypothetical protein DVH05_008562 [Phytophthora capsici]	40.24	54.68	30.59
p-cluster277164	KAE9271440.1	hypothetical protein PF008_g30354, partial [Phytophthora fragariae]	40.96	26.71	38.07
p-cluster277178	GAY04316.1	elongator complex protein putative [Pythium insidiosum]	71.79	60.69	63.37
p-cluster277406	GAY04631.1	acetolactate synthase, small subunit [Pythium insidiosum]	88.82	41.28	80.68
p-cluster277538	TDH68998.1	hypothetical protein CCR75_000927 [Bremia lactucae]	41.00	94.34	17.35
p-cluster277547	GAX94489.1	peroxiredoxin-4 [Pythium insidiosum]	88.26	22.39	48.09
p-cluster277595	TMW64064.1	hypothetical protein Poli38472_014181 [Pythium oligandrum]	55.68	58.28	10.42

p-cluster277629	RHZ29746.1	hypothetical protein DYB37_001402 [Aphanomyces astaci]	65.88	79.38	95.32
p-cluster277987	TMW59800.1	hypothetical protein Poli38472_004869 [Pythium oligandrum]	34.26	37.18	21.95
p-cluster278056	KAG7397444.1	hypothetical protein PHYBOEH_000723 [Phytophthora boehmeriae]	54.31	46.32	82.48
p-cluster278266	TYZ57587.1	hypothetical protein PybrP1_013208 [[Pythium] brassicae (nom. inval.)]	58.82	28.23	21.65
p-cluster278390	GAX94165.1	glucoamylase GlaM [Pythium insidiosum]	91.45	44.97	47.78
p-cluster278777	GAY00549.1	pre-mRNA-splicing factor RSE1 [Pythium insidiosum]	75.00	95.19	4.41
p-cluster278911	GAX93679.1	putative secreted protein [Pythium insidiosum]	79.33	100.00	100.00
p-cluster279037	KAH9148429.1	hypothetical protein LEN26_004499, partial [Aphanomyces euteiches]	64.38	31.30	36.50
p-cluster279162	TMW63676.1	hypothetical protein Poli38472_002617 [Pythium oligandrum]	35.95	100.00	25.13
p-cluster279484	KAG6587276.1	hypothetical protein IUM83_02707 [Phytophthora cinnamomi]	83.62	15.47	98.86
p-cluster279528	KAG6609759.1	Microfibrillar-associated protein 1 [Phytophthora cinnamomi]	66.00	25.39	10.44
p-cluster279533	RHZ34837.1	hypothetical protein DYB26_012314, partial [Aphanomyces astaci]	34.40	50.16	93.43
p-cluster279642	TMW67191.1	hypothetical protein Poli38472_012307 [Pythium oligandrum]	28.34	94.05	82.64
p-cluster279843	KAF1318637.1	hypothetical protein FI667_g13755, partial [Globisporangium splendens]	62.69	48.88	93.62
p-cluster279866	GAY04851.1	U4/U6 small nuclear ribonucleoprotein Prp3, putative [Pythium insidiosum]	70.70	100.00	36.95
p-cluster279903	KAF1332213.1	hypothetical protein FI667_g3809, partial [Globisporangium splendens]	38.51	46.61	47.66
p-cluster280398	TMW67441.1	hypothetical protein Poli38472_011061 [Pythium oligandrum]	55.41	36.35	61.03
p-cluster280559	TMW69700.1	hypothetical protein Poli38472_001856 [Pythium oligandrum]	58.44	25.41	34.68
p-cluster280703	TMW67954.1	hypothetical protein Poli38472_007626 [Pythium oligandrum]	80.12	43.38	98.85
p-cluster280772	XP_045611902.1	basic proline-rich protein-like [Procambarus clarkii]	37.46	47.80	33.25
p-cluster280778	TMW56043.1	hypothetical protein Poli38472_008691 [Pythium oligandrum]	70.71	69.15	33.50
p-cluster280816	TMW67485.1	hypothetical protein Poli38472_011105 [Pythium oligandrum]	37.50	76.15	18.66
p-cluster280960	KAE8913308.1	hypothetical protein PF003_g2393 [Phytophthora fragariae]	34.34	24.62	45.21
p-cluster281001	GAY02013.1	elicitin-like protein BRL1A [Pythium insidiosum]	80.28	94.55	89.08
p-cluster281251	OQR98765.1	hypothetical protein THRCLA_21870 [Thraustotheca clavata]	24.22	63.05	35.23
p-cluster281555	GAY00204.1	F-actin-capping protein subunit beta, putative [Pythium insidiosum]	80.88	97.14	7.72
p-cluster281675	TMW69287.1	hypothetical protein Poli38472_001443 [Pythium oligandrum]	45.21	43.45	10.29
p-cluster281749	RLN62533.1	hypothetical protein BBP00_00004701 [Phytophthora kernoviae]	72.22	40.74	11.36
p-cluster281952	KAG7395501.1	hypothetical protein PHYBOEH_003692 [Phytophthora boehmeriae]	41.84	44.78	40.09
p-cluster282198	GAX93036.1	threonine dehydratase catabolic putative [Pythium insidiosum]	100.00	47.37	2.88
p-cluster282404	TMW66839.1	hypothetical protein Poli38472_011955 [Pythium oligandrum]	43.19	44.22	67.62
p-cluster282512	TMW69636.1	hypothetical protein Poli38472_001792 [Pythium oligandrum]	51.69	37.77	44.20
p-cluster282513	GAX97987.1	cytochrome P450, putative [Pythium insidiosum]	87.20	40.45	67.89
p-cluster282531	TMW58033.1	hypothetical protein Poli38472_013507 [Pythium oligandrum]	34.24	95.51	36.69
p-cluster282664	GAX96280.1	homebox domain-containing protein [Pythium insidiosum]	92.14	64.52	22.29
p-cluster282740	KAG2532116.1	hypothetical protein JM16_000494 [Phytophthora kernoviae]	40.00	100.00	50.26
p-cluster283019	TMW66982.1	hypothetical protein Poli38472_012098 [Pythium oligandrum]	61.63	46.23	91.37
p-cluster283083	TMW64390.1	hypothetical protein Poli38472_013012 [Pythium oligandrum]	65.52	44.62	25.22
p-cluster283301	TMW59996.1	hypothetical protein Poli38472_000038 [Pythium oligandrum]	65.83	27.11	12.78
p-cluster283577	TMW57971.1	hypothetical protein Poli38472_013445 [Pythium oligandrum]	59.16	28.73	59.62
p-cluster283637	TMW56825.1	hypothetical protein Poli38472_006835 [Pythium oligandrum]	28.18	23.16	28.88
p-cluster283644	TMW66084.1	hypothetical protein Poli38472_003849 [Pythium oligandrum]	54.80	44.17	33.48
p-cluster283646	KAG2996115.1	hypothetical protein PC118_g2650 [Phytophthora cactorum]	57.14	86.90	39.69
p-cluster283808	GAX99263.1	CMGC/DYRK/YAK protein kinase [Pythium insidiosum]	73.45	97.29	26.43
p-cluster283897	TMW63930.1	hypothetical protein Poli38472_014635 [Pythium oligandrum]	41.84	43.52	64.23
p-cluster283898	GAY02261.1	eukaryotic translation initiation factor 3 subunit, putative [Pythium insidiosum]	93.23	48.49	99.86
p-cluster284054	KAG7390469.1	hypothetical protein PHYBOEH_007029 [Phytophthora boehmeriae]	52.04	98.68	74.68
p-cluster284101	KAF1315985.1	hypothetical protein FI667_g15689, partial [Globisporangium splendens]	48.26	39.19	33.66
p-cluster284150	XP_002909107.1	myb-like DNA-binding protein, putative [Phytophthora infestans T30-4]	48.72	22.19	37.19
p-cluster284302	KAF1328331.1	hypothetical protein FI667_g6951, partial [Globisporangium splendens]	33.50	58.89	61.13
p-cluster284431	GAY02843.1	thioredoxin-like protein [Pythium insidiosum]	78.72	94.32	26.22
p-cluster284544	TMW57872.1	hypothetical protein Poli38472_013346 [Pythium oligandrum]	39.45	65.16	32.16
p-cluster284663	GAX97001.1	inositol monophosphatase, putative [Pythium insidiosum]	89.40	100.00	28.31
p-cluster284866	GAX96532.1	calmodulin-like protein [Pythium insidiosum]	80.15	50.97	82.31
p-cluster285045	XP_024574916.1	CRN-like protein [Plasmopara halstedii]CEG38547.1 CRN-like protein [Plasmopara halstedii]	35.55	26.90	33.01
p-cluster285096	KAI5642356.1	SEA domain-containing protein [Phthorimaea operculella]	43.90	31.61	9.00
p-cluster285311	TMW66792.1	hypothetical protein Poli38472_014104 [Pythium oligandrum]	45.03	62.16	14.65
p-cluster285333	KAG1700736.1	hypothetical protein DVH05_011595 [Phytophthora capsici]	41.11	100.00	58.39
p-cluster285448	TMW66842.1	hypothetical protein Poli38472_011958 [Pythium oligandrum]	38.21	36.18	18.59
p-cluster285533	TMW68094.1	hypothetical protein Poli38472_007766 [Pythium oligandrum]	64.00	21.93	5.40
p-cluster285703	KAG7383577.1	hypothetical protein PHYPSEUDO_003556 [Phytophthora pseudosyringae]	58.54	47.98	7.96
p-cluster285933	TMW64732.1	hypothetical protein Poli38472_011612 [Pythium oligandrum]	34.61	85.28	27.03

p-cluster286727	TMW67123.1	hypothetical protein Poli38472_012239 [Pythium oligandrum]	32.39	78.60	69.50
p-cluster286829	TMW62866.1	hypothetical protein Poli38472_005484 [Pythium oligandrum]	48.48	45.96	17.41
p-cluster286857	MBR3273666.1	Cna B-type domain-containing protein [Clostridia bacterium]	34.10	71.43	5.18
p-cluster286954	TYZ64100.1	hypothetical protein PybrP1_009127 [[Pythium] brassicae (nom. inval.)]	48.31	33.29	49.47
p-cluster287215	TMW67345.1	hypothetical protein Poli38472_012461 [Pythium oligandrum]	59.93	72.70	34.06
p-cluster287232	TMW57575.1	hypothetical protein Poli38472_003500 [Pythium oligandrum]	30.31	98.22	52.16
p-cluster287320	GAY00751.1	glycoprotein elicitor-like protein [Pythium insidiosum]	72.34	50.81	24.72
p-cluster287361	KAG1703749.1	hypothetical protein DVH05_006764 [Phytophthora capsici]	64.52	33.88	10.32
p-cluster287511	TMW57636.1	hypothetical protein Poli38472_003561 [Pythium oligandrum]	54.85	72.19	65.65
p-cluster287592	TMW55555.1	hypothetical protein Poli38472_010437 [Pythium oligandrum]	55.41	41.01	28.46
p-cluster287664	KAG7392418.1	hypothetical protein PHYPSEUDO_000826 [Phytophthora pseudosyringae]	46.84	34.65	3.59
p-cluster287777	TMW63914.1	hypothetical protein Poli38472_014619 [Pythium oligandrum]	68.92	25.69	13.81
p-cluster288044	TMW57703.1	hypothetical protein Poli38472_014306 [Pythium oligandrum]	71.43	39.85	63.89
p-cluster288247	KAF1334195.1	Hect-domain containing peotein, partial [Globisporangium splendens]	39.13	34.18	2.60
p-cluster288771	KAF1319008.1	Nucleoredoxin, partial [Globisporangium splendens]	44.44	54.14	7.79
p-cluster288943	TMW55464.1	hypothetical protein Poli38472_010346 [Pythium oligandrum]	76.92	34.10	18.84
p-cluster288946	KAG3254073.1	hypothetical protein PI124_g1312 [Phytophthora idaei]	43.03	99.70	9.27
p-cluster289106	KAG1701159.1	hypothetical protein DVH05_011397 [Phytophthora capsici]	54.82	96.46	3.31
p-cluster289133	GAX92781.1	TKL protein kinase [Pythium insidiosum]	68.32	46.92	9.87
p-cluster289652	RLN32079.1	hypothetical protein BBJ28_00020207, partial [Nothophytophthora sp. Chile5]	55.56	72.26	71.32
p-cluster289777	TMW63729.1	hypothetical protein Poli38472_002670 [Pythium oligandrum]	31.78	96.19	22.80
p-cluster289796	TMW57596.1	hypothetical protein Poli38472_003521 [Pythium oligandrum]	35.71	85.58	6.44
p-cluster289897	TMW66169.1	hypothetical protein Poli38472_003934 [Pythium oligandrum]	42.03	37.30	3.87
p-cluster289919	RLN64429.1	hypothetical protein BBJ29_002015 [Phytophthora kernoviae]	30.53	90.97	16.86
p-cluster289994	GAX92749.1	presenilin-like protein [Pythium insidiosum]	93.98	100.00	14.61
p-cluster290022	TMW61735.1	hypothetical protein Poli38472_010798 [Pythium oligandrum]	70.59	45.27	8.63
p-cluster290129	TMW55465.1	hypothetical protein Poli38472_010347 [Pythium oligandrum]	46.46	59.76	19.64
p-cluster290160	GAX93438.1	TKL protein kinase [Pythium insidiosum]	88.98	100.00	39.43
p-cluster290197	TMW57162.1	hypothetical protein Poli38472_003087 [Pythium oligandrum]	76.19	67.31	10.76
p-cluster290208	GAX95191.1	glycosylltransferase-like domain-containing protein 1-like protein [Pythium insidiosum]	41.76	100.00	6.18
p-cluster290290	XP_002909239.1	conserved hypothetical protein [Phytophthora infestans T30-4]	39.36	84.68	6.04
p-cluster290331	TMW57162.1	hypothetical protein Poli38472_003087 [Pythium oligandrum]	48.59	100.00	14.66
p-cluster290480	KAG9403748.1	hypothetical protein AC1031_005238 [Aphanomyces cochlioides]	49.58	95.20	0.61
p-cluster290491	TMW57161.1	hypothetical protein Poli38472_003086 [Pythium oligandrum]	70.91	46.61	2.42
p-cluster291029	TMW58064.1	hypothetical protein Poli38472_013538 [Pythium oligandrum]	39.81	33.12	54.05
p-cluster291048	GAY02043.1	protein CREG1 precursor [Pythium insidiosum]	85.85	100.00	84.13
p-cluster291194	TMW65734.1	hypothetical protein Poli38472_008376 [Pythium oligandrum]	64.10	42.86	2.00
p-cluster291271	XP_002998910.1	hypothetical protein PITG_23301 [Phytophthora infestans T30-4]	38.10	23.28	52.29
p-cluster291541	RLN64016.1	hypothetical protein BBP00_00003719 [Phytophthora kernoviae]	42.86	31.40	70.25
p-cluster291614	TMW62172.1	hypothetical protein Poli38472_009665 [Pythium oligandrum]	34.87	74.63	83.33
p-cluster291814	TMW59836.1	hypothetical protein Poli38472_004905 [Pythium oligandrum]	27.17	97.73	53.50
p-cluster292214	TMW55330.1	hypothetical protein Poli38472_013221 [Pythium oligandrum]	45.45	91.04	29.77
p-cluster292285	TMW57096.1	hypothetical protein Poli38472_003021 [Pythium oligandrum]	62.16	7.63	2.76
p-cluster292345	TMW55376.1	hypothetical protein Poli38472_013267 [Pythium oligandrum]	46.13	74.17	64.35
p-cluster292589	KAE9020132.1	hypothetical protein PR002_g12609, partial [Phytophthora rubi]	70.45	35.34	32.00
p-cluster292611	TMW64745.1	hypothetical protein Poli38472_011625 [Pythium oligandrum]	32.85	23.00	19.26
p-cluster293156	TMW62073.1	hypothetical protein Poli38472_009566 [Pythium oligandrum]	60.82	38.90	100.00
p-cluster293392	TMW67111.1	hypothetical protein Poli38472_012227 [Pythium oligandrum]	50.46	93.75	22.71
p-cluster293428	TMW64088.1	hypothetical protein Poli38472_014205 [Pythium oligandrum]	32.65	64.47	24.37
p-cluster293502	TMW64925.1	hypothetical protein Poli38472_009092 [Pythium oligandrum]	42.34	87.64	32.12
p-cluster293618	TMW59419.1	hypothetical protein Poli38472_004488 [Pythium oligandrum]	39.30	63.94	59.98
p-cluster293667	TMW65751.1	hypothetical protein Poli38472_008393 [Pythium oligandrum]	34.69	94.96	27.10
p-cluster293767	TMW68431.1	hypothetical protein Poli38472_005899 [Pythium oligandrum]	42.03	99.75	32.36
p-cluster293899	GAX94892.1	peptidase inhibitor 16 isoform X3 [Pythium insidiosum]	96.18	94.58	29.77
p-cluster294014	TMW68134.1	hypothetical protein Poli38472_007806 [Pythium oligandrum]	34.19	48.59	66.08
p-cluster294170	KAE8331783.1	hypothetical protein BDV39DRAFT_218285 [Aspergillus sergii]	31.55	98.20	23.45
p-cluster294343	ETO58943.1	hypothetical protein F444_22680, partial [Phytophthora parasitica P1976]	42.86	90.91	85.37
p-cluster294354	KAF1331613.1	Glutathione-disulfide reductase, partial [Globisporangium splendens]	41.48	53.13	8.55
p-cluster294598	KAF5925127.1	hypothetical protein HPG69_008804, partial [Diceros bicornis minor]	60.78	11.28	44.35
p-cluster294691	GAX93491.1	sporangia induced 28 kDa inner dynein arm light chain [Pythium insidiosum]	90.63	30.77	2.37
p-cluster294877	TMW68318.1	hypothetical protein Poli38472_005786 [Pythium oligandrum]	38.92	27.02	17.63
p-cluster295180	TMW55656.1	hypothetical protein Poli38472_010538 [Pythium oligandrum]	44.88	99.59	23.56

p-cluster295268	TMW63572.1	hypothetical protein Poli38472_002513 [Pythium oligandrum]	36.11	44.61	17.57
p-cluster295516	KAG1695369.1	hypothetical protein DVH05_020407 [Phytophthora capsici]	38.51	55.06	39.35
p-cluster295527	TMW67370.1	hypothetical protein Poli38472_012486 [Pythium oligandrum]	34.44	90.03	31.03
p-cluster295655	TYZ65097.1	hypothetical protein PybrP1_006014 [[Pythium] brassicae (nom. inval.)]	48.72	53.06	12.81
p-cluster295701	TMW62925.1	hypothetical protein Poli38472_005543 [Pythium oligandrum]	60.00	17.37	23.73
p-cluster295707	TMW60715.1	hypothetical protein Poli38472_000757 [Pythium oligandrum]	56.32	97.18	15.49
p-cluster295847	TMW66206.1	hypothetical protein Poli38472_003971 [Pythium oligandrum]	48.09	69.68	32.93
p-cluster295926	TMW68805.1	hypothetical protein Poli38472_006273 [Pythium oligandrum]	63.04	20.72	4.13
p-cluster296237	TMW60909.1	hypothetical protein Poli38472_000951 [Pythium oligandrum]	55.83	28.30	9.85
p-cluster296357	KAF1315497.1	hypothetical protein FI667_g15994, partial [Globisporangium splendens]	25.05	95.79	41.00
p-cluster296396	KAG2523411.1	hypothetical protein JM16_005350 [Phytophthora kernoviae]	34.59	75.61	53.26
p-cluster296715	TMW69039.1	hypothetical protein Poli38472_001195 [Pythium oligandrum]	50.00	61.25	15.77
p-cluster296764	TMW60879.1	hypothetical protein Poli38472_000921 [Pythium oligandrum]	33.01	39.33	12.77
p-cluster296814	KAG7380998.1	hypothetical protein PHYPSEUDO_006551 [Phytophthora pseudosyringae]	29.29	54.43	51.47
p-cluster296897	TMW60867.1	hypothetical protein Poli38472_000909 [Pythium oligandrum]	33.07	54.21	8.55
p-cluster296946	OWY98720.1	hypothetical protein PHMEG_00030446, partial [Phytophthora megakarya]	34.02	67.35	63.51
p-cluster297225	GAX99195.1	microtubule -associated protein EB1 [Pythium insidiosum]	93.44	96.06	40.53
p-cluster297252	TMW56893.1	hypothetical protein Poli38472_002818 [Pythium oligandrum]	31.25	86.22	37.31
p-cluster297260	TMW68749.1	hypothetical protein Poli38472_006217 [Pythium oligandrum]	36.99	80.50	51.49
p-cluster297285	GAY06400.1	putative membrane protein [Pythium insidiosum]	89.47	42.54	41.91
p-cluster297420	TMW66528.1	hypothetical protein Poli38472_004293 [Pythium oligandrum]	33.94	79.02	18.31
p-cluster297466	TMW65820.1	hypothetical protein Poli38472_003585 [Pythium oligandrum]	37.58	46.20	24.62
p-cluster297545	TMW57089.1	hypothetical protein Poli38472_003014 [Pythium oligandrum]	69.81	60.23	16.19
p-cluster297551	TMW68782.1	hypothetical protein Poli38472_006250 [Pythium oligandrum]	40.89	87.08	26.46
p-cluster297589	TMW64122.1	hypothetical protein Poli38472_014239 [Pythium oligandrum]	47.46	62.11	9.61
p-cluster297631	GAX97551.1	CK1/TTBK protein kinase [Pythium insidiosum]	71.57	33.01	21.54
p-cluster297820	GAY04436.1	pepsin-like aspartic protease A1 [Pythium insidiosum]	94.12	100.00	4.34
p-cluster297829	TMW60723.1	hypothetical protein Poli38472_000765 [Pythium oligandrum]	35.42	66.83	13.89
p-cluster297836	TMW64861.1	hypothetical protein Poli38472_009028 [Pythium oligandrum]	47.89	97.15	9.16
p-cluster297887	KAG1684238.1	hypothetical protein DVH05_011515 [Phytophthora capsici]	32.10	97.67	10.34
p-cluster297923	TMW63364.1	hypothetical protein Poli38472_002305 [Pythium oligandrum]	36.28	72.18	12.41
p-cluster298074	TMW60726.1	hypothetical protein Poli38472_000768 [Pythium oligandrum]	38.27	87.07	40.22
p-cluster298202	TMW65024.1	hypothetical protein Poli38472_009191 [Pythium oligandrum]	53.21	35.05	16.70
p-cluster298220	TMW68259.1	hypothetical protein Poli38472_005727 [Pythium oligandrum]	47.57	44.33	10.67
p-cluster298392	TMW62127.1	hypothetical protein Poli38472_009620 [Pythium oligandrum]	36.73	46.73	15.72
p-cluster298438	GAX99197.1	5'-AMP-activated protein kinase subunit beta, putative [Pythium insidiosum]	100.00	100.00	9.26
p-cluster298443	GAY05760.1	tubulin-tyrosine ligase family, putative [Pythium insidiosum]	66.67	68.60	20.46
p-cluster298538	KAE8935973.1	hypothetical protein PF009_g14101 [Phytophthora fragariae]	57.50	64.91	69.64
p-cluster298608	KAF1325166.1	hypothetical protein FI667_g9423, partial [Globisporangium splendens]	35.84	68.73	38.18
p-cluster298733	TYZ57356.1	hypothetical protein PybrP1_012956 [[Pythium] brassicae (nom. inval.)]	76.00	22.61	23.51
p-cluster298736	TMW66734.1	hypothetical protein Poli38472_014046 [Pythium oligandrum]	67.65	48.15	3.91
p-cluster298993	TMW57730.1	hypothetical protein Poli38472_014333 [Pythium oligandrum]	32.75	95.28	9.24
p-cluster299062	TMW67764.1	hypothetical protein Poli38472_007436 [Pythium oligandrum]	31.34	99.53	20.44
p-cluster299134	TMW58666.1	hypothetical protein Poli38472_010225 [Pythium oligandrum]	53.64	29.62	10.09
p-cluster299176	RLN96599.1	hypothetical protein BBJ28_00000145 [Nothophytophthora sp. Chile5]	40.33	83.75	51.93
p-cluster299190	TMW68737.1	hypothetical protein Poli38472_006205 [Pythium oligandrum]	51.11	68.70	14.58
p-cluster299229	TMW55216.1	hypothetical protein Poli38472_013107 [Pythium oligandrum]	35.17	89.92	33.18
p-cluster299270	GAY02104.1	protein kinase, putative [Pythium insidiosum]	53.44	46.45	21.76
p-cluster299619	KAH7474589.1	hypothetical protein KRP23_8717 [Phytophthora ramorum]	34.67	99.34	26.04
p-cluster299646	TMW57288.1	hypothetical protein Poli38472_003213 [Pythium oligandrum]	26.29	71.34	21.53
p-cluster299670	TMW62668.1	hypothetical protein Poli38472_005286 [Pythium oligandrum]	53.98	78.93	44.29
p-cluster299672	KAH9244766.1	hypothetical protein BASA81_017802 [Batrachochytrium salamandrivorans]	26.77	77.47	20.88
p-cluster299750	TMW66086.1	hypothetical protein Poli38472_003851 [Pythium oligandrum]	31.23	100.00	26.81
p-cluster299786	KAH8055401.1	phosphatidylinositol kinase [Aureococcus anophagefferens]	45.83	20.81	4.28
p-cluster299858	TMW55768.1	hypothetical protein Poli38472_010650 [Pythium oligandrum]	87.80	36.94	8.61
p-cluster299895	TMW60190.1	hypothetical protein Poli38472_000232 [Pythium oligandrum]	54.05	28.17	8.87
p-cluster299926	TMW65080.1	hypothetical protein Poli38472_009247 [Pythium oligandrum]	39.38	95.03	23.47
p-cluster299948	TMW69106.1	hypothetical protein Poli38472_001262 [Pythium oligandrum]	33.33	44.64	21.99
p-cluster299980	RLN71598.1	hypothetical protein BBJ28_00022286 [Nothophytophthora sp. Chile5]	66.67	25.14	10.65
p-cluster300214	OWY96393.1	Acyl-CoA synthetase [Phytophthora megakarya]	53.54	55.75	22.47
p-cluster300224	TMW64941.1	hypothetical protein Poli38472_009108 [Pythium oligandrum]	40.58	36.56	13.28
p-cluster300347	KAF1318639.1	Transcription factor skn7, partial [Globisporangium splendens]	37.17	80.46	30.28

p-cluster300437	KAF1327428.1	hypothetical protein FI667_g7709, partial [Globisporangium splendens]	50.00	72.04	23.00
p-cluster300459	CCI43339.1	unnamed protein product [Albugo candida]	32.58	98.81	60.85
p-cluster300493	TMW55297.1	hypothetical protein Poli38472_013188 [Pythium oligandrum]	45.33	100.00	6.11
p-cluster300497	TMW63211.1	hypothetical protein Poli38472_002152 [Pythium oligandrum]	34.98	99.61	23.03
p-cluster300608	TMW65836.1	hypothetical protein Poli38472_003601 [Pythium oligandrum]	58.97	35.17	11.01
p-cluster300613	RLN90400.1	hypothetical protein BBJ28_00014818 [Nothophytophthora sp. Chile5]	36.17	38.10	16.95
p-cluster300751	TMW57697.1	hypothetical protein Poli38472_014300 [Pythium oligandrum]	27.78	82.49	11.97
p-cluster300826	TMW56286.1	hypothetical protein Poli38472_008934 [Pythium oligandrum]	41.39	67.90	11.97
p-cluster300837	KAF1330191.1	hypothetical protein FI667_g5395, partial [Globisporangium splendens]	47.56	41.00	25.11
p-cluster301042	TMW55993.1	hypothetical protein Poli38472_008641 [Pythium oligandrum]	44.00	43.26	13.22
p-cluster301071	TMW60410.1	hypothetical protein Poli38472_000452 [Pythium oligandrum]	41.70	86.81	33.38
p-cluster301092	GAX96781.1	transmembrane protein, putative [Pythium insidiosum]	88.89	47.79	13.93
p-cluster301097	RLN92793.1	hypothetical protein BBJ28_00012997 [Nothophytophthora sp. Chile5]	28.40	97.38	22.88
p-cluster301250	TMW57503.1	hypothetical protein Poli38472_003428 [Pythium oligandrum]	71.15	38.52	8.33
p-cluster301286	TMW61787.1	hypothetical protein Poli38472_010850 [Pythium oligandrum]	63.06	56.35	10.69
p-cluster301313	TMW69603.1	hypothetical protein Poli38472_001759 [Pythium oligandrum]	74.47	49.47	10.80
p-cluster301412	TMW60541.1	hypothetical protein Poli38472_000583 [Pythium oligandrum]	76.60	55.29	19.11
p-cluster301438	TMW55033.1	hypothetical protein Poli38472_013795 [Pythium oligandrum]	67.65	77.62	31.62
p-cluster301473	TMW60488.1	hypothetical protein Poli38472_000530 [Pythium oligandrum]	49.31	44.09	16.83
p-cluster301474	TMW68155.1	hypothetical protein Poli38472_007827 [Pythium oligandrum]	56.12	30.38	12.37
p-cluster301693	TMW56718.1	hypothetical protein Poli38472_006728 [Pythium oligandrum]	37.32	95.85	72.49
p-cluster301715	TMW55171.1	hypothetical protein Poli38472_013933 [Pythium oligandrum]	31.55	95.43	13.84
p-cluster301719	TMW63272.1	hypothetical protein Poli38472_002213 [Pythium oligandrum]	38.03	85.08	20.91
p-cluster301731	GAY01487.1	seryl-tRNA synthetase, partial [Pythium insidiosum]	82.08	37.19	19.09
p-cluster301817	TMW57994.1	hypothetical protein Poli38472_013468 [Pythium oligandrum]	37.58	100.00	6.87
p-cluster301878	TMW68220.1	hypothetical protein Poli38472_007892 [Pythium oligandrum]	52.15	97.49	40.16
p-cluster301979	TMW69106.1	hypothetical protein Poli38472_001262 [Pythium oligandrum]	34.02	38.19	13.33
p-cluster302029	GAX95399.1	myosin-like protein [Pythium insidiosum]	93.55	100.00	23.02
p-cluster302040	TMW60290.1	hypothetical protein Poli38472_000332 [Pythium oligandrum]	50.00	44.30	4.46
p-cluster302057	GAX92271.1	condensin complex subunit 2, putative [Pythium insidiosum]	72.49	100.00	28.24
p-cluster302284	TMW56734.1	hypothetical protein Poli38472_006744 [Pythium oligandrum]	51.29	97.01	32.29
p-cluster302287	TMW59924.1	hypothetical protein Poli38472_004993 [Pythium oligandrum]	36.74	75.29	43.18
p-cluster302380	TMW65780.1	hypothetical protein Poli38472_008422 [Pythium oligandrum]	45.87	100.00	12.58
p-cluster302422	RJP29183.1	M20/M25/M40 family metallo-hydrolase [Candidatus Omnitrophica bacterium]	23.75	92.83	4.43
p-cluster302450	KAE9271432.1	hypothetical protein PR003_g30509 [Phytophthora rubi]	24.91	99.15	64.85
p-cluster302517	GAX96830.1	26S protease regulatory subunit 6B [Pythium insidiosum]	84.00	24.24	2.92
p-cluster302535	KAF1327061.1	hypothetical protein FI667_g7917, partial [Globisporangium splendens]	28.16	51.16	9.07
p-cluster302546	TMW56087.1	hypothetical protein Poli38472_008735 [Pythium oligandrum]	34.15	42.96	12.09
p-cluster302601	KXS08907.1	hypothetical protein M427DRAFT_65057 [Gonapodya prolifera JEL478]	48.10	52.78	32.51
p-cluster302657	TMW69508.1	hypothetical protein Poli38472_001664 [Pythium oligandrum]	37.44	100.00	12.57
p-cluster302770	GAY06183.1	glutathione S-transferase, putative [Pythium insidiosum]	82.98	61.04	16.91
p-cluster302793	KAE8983591.1	hypothetical protein PR001_g23407 [Phytophthora rubi]	31.72	79.39	19.89
p-cluster302818	TMW69466.1	hypothetical protein Poli38472_001622 [Pythium oligandrum]	54.31	48.36	18.53
p-cluster302829	GAX92932.1	copper-translocating P-type ATPase [Pythium insidiosum]	96.15	26.35	8.99
p-cluster302880	TMW66192.1	hypothetical protein Poli38472_003957 [Pythium oligandrum]	43.13	98.58	14.80
p-cluster302882	KAH7469081.1	E3 ubiquitin ligase PQT3-like [Phytophthora ramorum]	54.34	99.32	23.39
p-cluster303015	GAX98959.1	argininosuccinate synthase [Pythium insidiosum]	93.18	42.31	6.12
p-cluster303087	TMW64468.1	hypothetical protein Poli38472_011348 [Pythium oligandrum]	39.90	75.89	27.34
p-cluster303230	GAX99814.1	tRNA pseudouridine synthase, putative [Pythium insidiosum]	88.89	65.45	10.50
p-cluster303382	TMW56211.1	hypothetical protein Poli38472_008859 [Pythium oligandrum]	45.76	99.23	39.75
p-cluster303418	KAG7400134.1	WD repeat-containing protein 70 [Phytophthora boehmeriae]	54.31	100.00	14.97
p-cluster303672	CCA15657.1	conserved hypothetical protein [Albugo laibachii Nc14]	44.19	91.40	14.74
p-cluster303749	TMW69126.1	hypothetical protein Poli38472_001282 [Pythium oligandrum]	45.90	46.25	11.97
p-cluster303867	KAF1314612.1	Snf2 family helicase/atpase and f-box protein, partial [Globisporangium splendens]	61.68	55.68	3.43
p-cluster303947	GAY04452.1	mucin-like protein [Pythium insidiosum]	93.87	100.00	27.59
p-cluster303960	GAX94275.1	phox domain-containing protein [Pythium insidiosum]	91.75	100.00	13.49
p-cluster303977	TMW56503.1	hypothetical protein Poli38472_006513 [Pythium oligandrum]	39.46	76.75	7.43
p-cluster303985	GAY01804.1	CMGC/DYRK/DYRK2 protein kinase [Pythium insidiosum]	78.07	100.00	32.92
p-cluster304092	TMW64946.1	hypothetical protein Poli38472_009113 [Pythium oligandrum]	37.41	93.04	12.96
p-cluster304170	TMW56286.1	hypothetical protein Poli38472_008934 [Pythium oligandrum]	36.36	94.17	9.01
p-cluster304274	TMW55752.1	hypothetical protein Poli38472_010634 [Pythium oligandrum]	37.50	100.00	19.89
p-cluster304302	KAF1314693.1	hypothetical protein FI667_g16455, partial [Globisporangium splendens]	35.24	80.38	30.47

p-cluster304364	GAX95965.1	DNA repair endonuclease XPF, putative [Pythium insidiosum]	75.00	70.59	5.56
p-cluster304393	GAY04489.1	trehalose-phosphatase [Pythium insidiosum]	81.01	75.22	12.74
p-cluster304446	TMW60026.1	hypothetical protein Poli38472_000068 [Pythium oligandrum]	62.32	27.06	10.52
p-cluster304514	TMW56286.1	hypothetical protein Poli38472_008934 [Pythium oligandrum]	46.24	49.21	4.04
p-cluster304663	KAF8819081.1	Rna recognition motif-containing protein, partial [Cardiosporidium cionae]	46.55	56.86	32.22
p-cluster304674	GAX94533.1	chromatin assembly factor 1 subunit B-like protein [Pythium insidiosum]	76.00	100.00	11.74
p-cluster304743	KAF1313140.1	Integrase catalytic core protein, partial [Globisporangium splendens]	45.64	58.20	8.16
p-cluster304748	TMW55415.1	hypothetical protein Poli38472_013306 [Pythium oligandrum]	43.88	67.97	19.91
p-cluster304807	TMW56887.1	hypothetical protein Poli38472_002812 [Pythium oligandrum]	55.00	98.21	4.43
p-cluster304818	TMW66181.1	hypothetical protein Poli38472_003946 [Pythium oligandrum]	48.39	100.00	23.93
p-cluster304938	GAY00823.1	peroxisome assembly factor, putative, partial [Pythium insidiosum]	70.72	100.00	42.54
p-cluster304960	TMW56434.1	hypothetical protein Poli38472_006444 [Pythium oligandrum]	75.61	29.93	6.81
p-cluster305105	TMW64682.1	hypothetical protein Poli38472_011562 [Pythium oligandrum]	30.14	89.13	23.54
p-cluster305439	TMW58703.1	hypothetical protein Poli38472_010262 [Pythium oligandrum]	39.16	68.93	24.51
p-cluster305461	TMW60164.1	hypothetical protein Poli38472_000206 [Pythium oligandrum]	43.87	82.57	41.19
p-cluster305496	GAY05730.1	serine protease family S33, putative [Pythium insidiosum]	51.76	98.84	14.31
p-cluster305571	RLN92091.1	hypothetical protein BBJ28_00024042 [Nothophytophthora sp. Chile5]	37.36	94.83	28.27
p-cluster305577	TMW55578.1	hypothetical protein Poli38472_010460 [Pythium oligandrum]	45.78	57.04	18.65
p-cluster305638	TMW68545.1	hypothetical protein Poli38472_006013 [Pythium oligandrum]	24.47	99.47	7.69
p-cluster305974	TMW68003.1	hypothetical protein Poli38472_007675 [Pythium oligandrum]	35.47	91.42	13.87
p-cluster305995	RLN47135.1	hypothetical protein BBJ29_009788, partial [Phytophthora kernoviae]	97.62	22.58	73.68
p-cluster306015	KAF1319605.1	Heat shock transcription factor, other eukaryote, partial [Globisporangium splendens]	33.52	91.33	28.08
p-cluster306049	TMW61592.1	hypothetical protein Poli38472_012783 [Pythium oligandrum]	55.28	53.25	13.41
p-cluster306120	KAF1319505.1	Fimbrin-like protein, partial [Globisporangium splendens]	49.52	87.95	19.69
p-cluster306185	TMW55577.1	hypothetical protein Poli38472_010459 [Pythium oligandrum]	55.56	39.66	22.09
p-cluster306187	XP_002899156.1	peroxisomal targeting signal 2 receptor, putative [Phytophthora infestans T30-4]	48.54	100.00	17.95
p-cluster306188	TMW68755.1	hypothetical protein Poli38472_006223 [Pythium oligandrum]	72.50	20.62	3.28
p-cluster306206	XP_009525676.1	hypothetical protein PHYSODRAFT_314345 [Phytophthora sojae]	71.74	36.22	30.07
p-cluster306236	TMW55994.1	hypothetical protein Poli38472_008642 [Pythium oligandrum]	45.90	51.26	6.62
p-cluster306345	TMW64410.1	hypothetical protein Poli38472_013032 [Pythium oligandrum]	44.87	100.00	6.81
p-cluster306356	RLN15274.1	hypothetical protein BBJ28_00016984 [Nothophytophthora sp. Chile5]	62.35	37.33	6.02
p-cluster306479	TMW57227.1	hypothetical protein Poli38472_003152 [Pythium oligandrum]	46.07	54.27	8.20
p-cluster306486	GAY04368.1	calpain-like protein [Pythium insidiosum]	83.04	100.00	20.53
p-cluster306521	GAX96765.1	zinc finger BED domain-containing protein 1-like protein [Pythium insidiosum]	82.57	100.00	21.99
p-cluster306617	TMW62162.1	hypothetical protein Poli38472_009655 [Pythium oligandrum]	46.53	63.55	21.13
p-cluster306634	KAE9021744.1	hypothetical protein PF011_g4792 [Phytophthora fragariae]	35.11	90.64	5.92
p-cluster306678	TMW67351.1	hypothetical protein Poli38472_012467 [Pythium oligandrum]	39.56	79.09	15.63
p-cluster306738	TMW58526.1	hypothetical protein Poli38472_010085 [Pythium oligandrum]	33.79	95.41	26.57
p-cluster306742	TMW67612.1	hypothetical protein Poli38472_011232 [Pythium oligandrum]	53.16	36.07	13.39
p-cluster306757	CCA24130.1	conserved hypothetical protein [Albugo laibachii Nc14]	89.47	26.03	66.28
p-cluster306818	TMW66191.1	hypothetical protein Poli38472_003956 [Pythium oligandrum]	47.37	88.37	12.92
p-cluster306859	GAX92849.1	AGC/PKC protein kinase [Pythium insidiosum]	68.89	29.03	13.89
p-cluster306913	TMW62972.1	hypothetical protein Poli38472_005590 [Pythium oligandrum]	55.41	41.18	6.51
p-cluster306964	TMW56627.1	hypothetical protein Poli38472_006637 [Pythium oligandrum]	31.20	65.10	9.73
p-cluster307254	TMW65626.1	hypothetical protein Poli38472_008268 [Pythium oligandrum]	36.77	100.00	15.46
p-cluster307267	TMW64993.1	hypothetical protein Poli38472_009160 [Pythium oligandrum]	28.50	94.29	3.29
p-cluster307313	GAY00070.1	midasin-like protein [Pythium insidiosum]	77.11	100.00	11.57
p-cluster307435	GAX96895.1	dual specificity tyrosine-phosphorylation-regulated kinase, putative [Pythium insidiosum]	90.54	81.77	20.85
p-cluster307608	GAX98922.1	serine protease family S01A, putative [Pythium insidiosum]	84.80	100.00	21.66
p-cluster307621	TMW68371.1	hypothetical protein Poli38472_005839 [Pythium oligandrum]	46.15	69.03	15.71
p-cluster307796	TYZ58417.1	hypothetical protein PybrP1_004249 [[Pythium] brassicae (nom. inval.)]	40.63	98.70	16.33
p-cluster307854	GAX95438.1	LOW QUALITY PROTEIN: paternally-expressed gene 3 protein-like protein [Pythium insidiosum]	85.63	100.00	14.04
p-cluster307877	KAF1318786.1	hypothetical protein FI667_g13614, partial [Globisporangium splendens]	51.85	50.94	11.51
p-cluster307922	TMW61504.1	hypothetical protein Poli38472_012695 [Pythium oligandrum]	39.76	100.00	20.94
p-cluster307939	TMW60885.1	hypothetical protein Poli38472_000927 [Pythium oligandrum]	39.29	58.08	20.88
p-cluster307966	XP_009528850.1	hypothetical protein PHYSODRAFT_509365 [Phytophthora sojae]	43.75	37.50	10.21
p-cluster307970	GAX99781.1	2oxoglutarate and iron-dependent oxygenase domain containing 1, putative [Pythium insidiosum]	91.39	93.79	31.82
p-cluster307982	KAF1323388.1	Splicing factor 3a, partial [Globisporangium splendens]	41.71	84.26	12.49
p-cluster308010	TMW62229.1	hypothetical protein Poli38472_009722 [Pythium oligandrum]	42.07	66.50	14.96
p-cluster308052	KAF1324561.1	Udp-n-acetylglucosamine--peptide n-acetylglucosaminyltransferase sec, partial [Globisporangium splendens]	49.61	70.18	12.61
p-cluster308054	GAY05142.1	U4/U6 small nuclear ribonucleoprotein Prp31 [Pythium insidiosum]	79.73	48.37	4.34
p-cluster308099	TMW55532.1	hypothetical protein Poli38472_010414 [Pythium oligandrum]	49.73	100.00	13.64

p-cluster308192	GAY04828.1	myb-like DNA-binding protein, putative [Pythium insidiosum]	64.39	100.00	69.52
p-cluster308255	TYZ56896.1	hypothetical protein PybrP1_003621 [[Pythium] brassicae (nom. inval.)]	34.08	96.63	4.61
p-cluster308288	TMW63556.1	hypothetical protein Poli38472_002497 [Pythium oligandrum]	35.08	95.45	34.20
p-cluster308365	TMW66161.1	hypothetical protein Poli38472_003926 [Pythium oligandrum]	47.06	85.44	24.57
p-cluster308381	KAF0682770.1	hypothetical protein As57867_025056, partial [Aphanomyces stellatus]	37.93	70.53	8.33
p-cluster308389	GAX97376.1	CMGC/MAPK protein kinase [Pythium insidiosum]	89.36	36.43	8.06
p-cluster308479	GAX95191.1	glycosylltransferase-like domain-containing protein 1-like protein [Pythium insidiosum]	75.71	88.61	4.86
p-cluster308493	TMW67121.1	hypothetical protein Poli38472_012237 [Pythium oligandrum]	50.00	98.37	9.57
p-cluster308506	GAX97433.1	ATP-dependent RNA helicase uap56 [Pythium insidiosum]	97.30	38.54	8.83
p-cluster308534	KAF1314614.1	hypothetical protein FI667_g16536, partial [Globisporangium splendens]	36.31	96.53	49.26
p-cluster308641	GAX98343.1	phosphatidylinositol 3 and 4-kinase-like protein [Pythium insidiosum]	91.40	100.00	13.63
p-cluster308666	KAG7392016.1	hypothetical protein PHYPSEUDO_002725 [Phytophthora pseudosyringae]	35.29	93.52	43.48
p-cluster308676	TMW57181.1	hypothetical protein Poli38472_003106 [Pythium oligandrum]	56.86	100.00	32.81
p-cluster308690	TMW56503.1	hypothetical protein Poli38472_006513 [Pythium oligandrum]	56.14	31.32	1.97
p-cluster308823	GAX98334.1	pepsin-like aspartic protease A1 [Pythium insidiosum]	75.14	100.00	41.19
p-cluster308833	GAY03529.1	ULK/FUSED protein kinase [Pythium insidiosum]	74.10	100.00	42.38
p-cluster308898	RLN48464.1	hypothetical protein BBJ28_00016009 [Nothophytophthora sp. Chile5]	72.94	53.80	17.49
p-cluster309063	OQR84250.1	hypothetical protein ACHHYP_13676 [Achlya hypogyna]	73.53	20.24	3.98
p-cluster309294	GAX98073.1	TKL/DRK protein kinase [Pythium insidiosum]	95.77	100.00	16.14
p-cluster309322	MVQ45756.1	BspA family leucine-rich repeat surface protein [Roseburia intestinalis]	43.30	59.74	10.68
p-cluster309346	KAF1332194.1	Protein transporter sec31a, partial [Globisporangium splendens]	57.36	99.20	6.37
p-cluster309466	TMW59436.1	hypothetical protein Poli38472_004505 [Pythium oligandrum]	33.68	58.94	6.39
p-cluster309593	KAH8889929.1	ankyrin, partial [Thozetella sp. PMI_491]	39.33	87.25	93.18
p-cluster309621	GAX96594.1	patatin-like phospholipase, putative [Pythium insidiosum]	97.65	100.00	11.77
p-cluster309627	AGC65391.1	OCM1, partial [Pythium insidiosum]	94.57	100.00	18.51
p-cluster309654	KAE9214195.1	hypothetical protein PF002_g17735 [Phytophthora fragariae]	52.78	77.91	7.08
p-cluster309708	TMW65837.1	hypothetical protein Poli38472_003602 [Pythium oligandrum]	55.70	82.14	16.83
p-cluster309835	KAH7468496.1	hypothetical protein KRP23_10876 [Phytophthora ramorum]	35.29	99.33	13.89
p-cluster309859	GAY05085.1	exosome complex exonuclease RRP44, putative [Pythium insidiosum]	82.05	99.13	10.66
p-cluster309896	KAF1323987.1	Kelch repeat-containing protein, partial [Globisporangium splendens]	60.00	44.87	11.73
p-cluster310060	GAX95191.1	glycosylltransferase-like domain-containing protein 1-like protein [Pythium insidiosum]	82.64	82.88	8.40
p-cluster310076	XP_009518380.1	phosphatidylinositol 3-kinase tor2-like protein [Phytophthora sojae]	42.07	100.00	3.73
p-cluster310084	TMW63422.1	hypothetical protein Poli38472_002363 [Pythium oligandrum]	42.07	81.71	37.01
p-cluster310290	TMW64929.1	hypothetical protein Poli38472_009096 [Pythium oligandrum]	53.44	97.01	35.63
p-cluster310302	TMW64000.1	hypothetical protein Poli38472_014705 [Pythium oligandrum]	56.76	68.94	12.97
p-cluster310353	TMW69183.1	hypothetical protein Poli38472_001339 [Pythium oligandrum]	41.90	84.00	8.26
p-cluster310451	KAF1324005.1	polymerase, partial [Globisporangium splendens]	78.00	34.25	8.00
p-cluster310491	GAX98413.1	serine protease family S15, putative [Pythium insidiosum]	95.24	100.00	8.57
p-cluster310565	TMW68844.1	hypothetical protein Poli38472_006312 [Pythium oligandrum]	42.57	99.30	11.39
p-cluster310638	TYZ58766.1	hypothetical protein PybrP1_003335 [[Pythium] brassicae (nom. inval.)]	77.27	28.39	3.14
p-cluster310688	TMW63817.1	hypothetical protein Poli38472_002758 [Pythium oligandrum]	40.38	100.00	2.66
p-cluster310754	TMW60514.1	hypothetical protein Poli38472_000556 [Pythium oligandrum]	45.81	100.00	7.96
p-cluster310777	TMW66435.1	hypothetical protein Poli38472_004200 [Pythium oligandrum]	53.13	84.21	5.18
p-cluster310839	TMW68636.1	hypothetical protein Poli38472_006104 [Pythium oligandrum]	63.45	97.32	30.67
p-cluster310848	TMW59736.1	hypothetical protein Poli38472_004805 [Pythium oligandrum]	54.55	42.76	10.89
p-cluster310865	GAX94661.1	TKL protein kinase [Pythium insidiosum]	40.00	78.51	21.44
p-cluster310975	KAF1327416.1	hypothetical protein FI667_g7725, partial [Globisporangium splendens]	39.63	100.00	13.48
p-cluster311060	TMW55908.1	hypothetical protein Poli38472_008556 [Pythium oligandrum]	35.14	98.65	7.50
p-cluster311096	GAX99183.1	AP-3 complex subunit delta, putative [Pythium insidiosum]	89.69	100.00	9.55
p-cluster311113	KAF1314940.1	Krr1-interacting protein involved in 40s ribosome biogenesis, partial [Globisporangium splendens]	82.14	40.15	7.47
p-cluster311119	GAX92729.1	prefoldin, alpha subunit [Pythium insidiosum]	80.29	97.86	25.44
p-cluster311138	KAG7379867.1	hypothetical protein PHYPSEUDO_008055 [Phytophthora pseudosyringae]	41.18	98.64	25.93
p-cluster311162	TMW63512.1	hypothetical protein Poli38472_002453 [Pythium oligandrum]	37.59	87.07	10.28
p-cluster311227	TMW60821.1	hypothetical protein Poli38472_000863 [Pythium oligandrum]	79.59	33.56	20.16
p-cluster311243	GAY04489.1	trehalose-phosphatase [Pythium insidiosum]	85.33	98.68	5.07
p-cluster311262	TMW63502.1	hypothetical protein Poli38472_002443 [Pythium oligandrum]	30.08	91.72	13.70
p-cluster311313	TMW67103.1	hypothetical protein Poli38472_012219 [Pythium oligandrum]	82.61	52.27	22.77
p-cluster311395	TMW69535.1	hypothetical protein Poli38472_001691 [Pythium oligandrum]	44.14	94.44	13.57
p-cluster311407	TMW57252.1	hypothetical protein Poli38472_003177 [Pythium oligandrum]	39.07	100.00	8.97
p-cluster311448	POM72776.1	Mitochondrial Protein Translocase (MPT) Family, partial [Phytophthora palmivora var. palmivora]	83.82	51.52	22.82
p-cluster311461	TMW57194.1	hypothetical protein Poli38472_003119 [Pythium oligandrum]	68.09	67.14	18.73
p-cluster311470	TYZ61701.1	hypothetical protein PybrP1_005713 [[Pythium] brassicae (nom. inval.)]	61.11	46.15	6.46

p-cluster311528	KAG6576309.1	RRNA biogenesis protein rrp5 [Phytophthora cinnamomi]	42.07	100.00	6.89
p-cluster311722	GAX92473.1	AGC/PKG protein kinase [Pythium insidiosum]	94.38	64.03	3.12
p-cluster311843	TMW64836.1	hypothetical protein Poli38472_009003 [Pythium oligandrum]	67.35	43.75	4.02
p-cluster311987	TMW64307.1	hypothetical protein Poli38472_012929 [Pythium oligandrum]	35.17	99.25	11.82
p-cluster311994	TMW64993.1	hypothetical protein Poli38472_009160 [Pythium oligandrum]	62.67	52.99	1.20
p-cluster312015	TMW62617.1	hypothetical protein Poli38472_005235 [Pythium oligandrum]	80.77	39.39	23.21
p-cluster312034	GAX97370.1	cyclin-H, putative [Pythium insidiosum]	92.93	85.34	25.00
p-cluster312104	TMW60410.1	hypothetical protein Poli38472_000452 [Pythium oligandrum]	59.52	75.00	12.46
p-cluster312131	GAY03229.1	AGC protein kinase [Pythium insidiosum]	78.38	87.95	9.64
p-cluster312153	GAX93228.1	phosphomethylpyrimidine kinase [Pythium insidiosum]	95.37	100.00	10.44
p-cluster312291	GAX92922.1	PEK/GCN2 protein kinase, variant [Pythium insidiosum]	87.70	93.85	7.56
p-cluster312411	GAY01053.1	ARS binding protein 1 putative [Pythium insidiosum]	76.99	99.10	9.39
p-cluster312447	TMW55760.1	hypothetical protein Poli38472_010642 [Pythium oligandrum]	55.45	96.12	19.96
p-cluster312485	XP_002905218.1	conserved hypothetical protein [Phytophthora infestans T30-4]	52.99	98.40	40.95
p-cluster312586	TMW68006.1	hypothetical protein Poli38472_007678 [Pythium oligandrum]	53.66	68.64	5.53
p-cluster312589	XP_009829821.1	hypothetical protein H257_06330 [Aphanomyces astaci]	33.72	97.70	1.25
p-cluster312699	GAX97518.1	DNA polymerase alpha catalytic subunit, putative [Pythium insidiosum]	82.46	100.00	3.68
p-cluster312726	GAY02568.1	phosphatidylinositol 3 and 4-kinase-like protein [Pythium insidiosum]	85.37	82.83	8.81
p-cluster312758	KAF1336811.1	hypothetical protein FI667_g55, partial [Globisporangium splendens]	44.94	71.07	19.78
p-cluster312851	GAX99180.1	lariat debranching enzyme B [Pythium insidiosum]	92.59	36.49	3.54
p-cluster312871	TMW63677.1	hypothetical protein Poli38472_002618 [Pythium oligandrum]	59.62	46.43	6.03
p-cluster312894	TMW58054.1	hypothetical protein Poli38472_013528 [Pythium oligandrum]	54.20	100.00	14.10
p-cluster312922	TMW55124.1	hypothetical protein Poli38472_013886 [Pythium oligandrum]	54.05	98.67	2.64
p-cluster312968	RMX65345.1	hypothetical protein DD238_004648 [Peronospora effusa]	54.67	96.15	42.13
p-cluster313114	TYZ63996.1	hypothetical protein PybrP1_004231 [[Pythium] brassicae (nom. inval.)]	45.76	90.77	3.72
p-cluster313150	GAY00045.1	eukaryotic translation initiation factor 2-alpha kinase, putative [Pythium insidiosum]	70.00	78.26	15.33
p-cluster313151	TMW64692.1	hypothetical protein Poli38472_011572 [Pythium oligandrum]	55.38	59.38	6.63
p-cluster313229	TMW60416.1	hypothetical protein Poli38472_000458 [Pythium oligandrum]	51.67	100.00	11.54
p-cluster313329	TMW62031.1	hypothetical protein Poli38472_009524 [Pythium oligandrum]	42.50	100.00	4.01
p-cluster313371	TMW66164.1	hypothetical protein Poli38472_003929 [Pythium oligandrum]	50.00	76.58	4.94
p-cluster313383	XP_002909186.1	crinkler (CRN) family protein, putative [Phytophthora infestans T30-4]	69.81	100.00	7.99
p-cluster313461	GAX92473.1	AGC/PKG protein kinase [Pythium insidiosum]	84.62	100.00	2.25
p-cluster313476	GAY04452.1	mucin-like protein [Pythium insidiosum]	93.58	100.00	11.52
p-cluster313484	GAY03311.1	Nacetylatedalphalinked acidic dipeptidaselike protein putative, partial [Pythium insidiosum]	86.96	100.00	10.69
p-cluster313552	GAX96180.1	WD40 repeatcontaining protein putative [Pythium insidiosum]	96.00	56.82	36.50
p-cluster313646	KAE9089031.1	hypothetical protein PF010_g19158 [Phytophthora fragariae]	57.14	81.90	11.52
p-cluster313668	KAF1322365.1	hypothetical protein FI667_g11339, partial [Globisporangium splendens]	37.36	87.50	9.45
p-cluster313701	TMW62304.1	hypothetical protein Poli38472_009797 [Pythium oligandrum]	52.05	65.22	8.00
p-cluster313730	GAX95730.1	arf/Sar family, other [Pythium insidiosum]	81.93	93.02	18.20
p-cluster313906	GAX94469.1	U3 small nucleolar RNA-associated protein, putative [Pythium insidiosum]	95.74	61.04	5.66
p-cluster314023	KAG3109560.1	hypothetical protein PI125_g10816 [Phytophthora idaei]	30.74	45.55	58.33
p-cluster314120	TMW57791.1	hypothetical protein Poli38472_014394 [Pythium oligandrum]	43.40	43.95	63.53
p-cluster314429	RLN75934.1	hypothetical protein BBO99_00007958 [Phytophthora kernoviae]	32.35	60.37	99.73
p-cluster314500	KAF1317303.1	Tkl protein kinase, partial [Globisporangium splendens]	42.78	42.11	76.48
p-cluster314650	POM63269.1	hypothetical protein PHPALM_27441 [Phytophthora palmivora var. palmivora]	29.75	68.04	68.00
p-cluster314660	KAG1695784.1	hypothetical protein DVH05_019123 [Phytophthora capsici]	44.87	17.15	48.72
p-cluster314800	TMW56133.1	hypothetical protein Poli38472_008781 [Pythium oligandrum]	63.01	28.52	43.90
p-cluster314957	GAY04101.1	gastricsin [Pythium insidiosum]	87.65	81.73	61.13
p-cluster314960	OQS04938.1	hypothetical protein THRCLA_02872 [Thraustotheca clavata]	35.75	42.05	86.94
p-cluster314984	XP_009531023.1	hypothetical protein PHYSODRAFT_335352 [Phytophthora sojae]	27.09	27.07	32.43
p-cluster315039	GAY01419.1	delta l-pyrroline-5-carboxylate synthetase [Pythium insidiosum]	96.54	40.47	100.00
p-cluster315050	TMW66522.1	hypothetical protein Poli38472_004287 [Pythium oligandrum]	27.45	96.90	96.58
p-cluster315122	TMW64558.1	hypothetical protein Poli38472_011438 [Pythium oligandrum]	55.88	33.51	10.86
p-cluster315389	KUG00113.1	hypothetical protein AM587_10008221 [Phytophthora nicotianae]	31.64	75.00	17.38
p-cluster315465	KAG7379946.1	hypothetical protein PHYPSEUDO_008004 [Phytophthora pseudosyringae]	29.96	96.00	86.12
p-cluster315671	GAY05747.1	replication protein [Pythium insidiosum]	55.70	29.41	54.51
p-cluster315787	TMW56585.1	hypothetical protein Poli38472_006595 [Pythium oligandrum]	51.74	26.42	90.51
p-cluster315960	TMW62327.1	hypothetical protein Poli38472_009820 [Pythium oligandrum]	51.22	31.09	69.32
p-cluster315964	TMW62322.1	hypothetical protein Poli38472_009815 [Pythium oligandrum]	41.38	87.60	62.21
p-cluster316002	GAY00282.1	heat shock protein [Pythium insidiosum]	87.96	86.99	31.40
p-cluster316017	TMW64551.1	hypothetical protein Poli38472_011431 [Pythium oligandrum]	29.06	79.54	73.54
p-cluster316022	KAF1317546.1	hypothetical protein FI667_g14720, partial [Globisporangium splendens]	48.55	47.21	84.30

p-cluster316133	TMW64154.1	hypothetical protein Poli38472_014271 [Pythium oligandrum]	34.58	48.76	27.30
p-cluster316363	TMW57617.1	hypothetical protein Poli38472_003542 [Pythium oligandrum]	52.08	69.46	6.94
p-cluster316516	KAH9092025.1	hypothetical protein Ae201684P_011564 [Aphanomyces euteiches]	29.13	79.23	74.58
p-cluster316524	TMW57816.1	hypothetical protein Poli38472_014419 [Pythium oligandrum]	42.68	44.73	95.86
p-cluster316613	TMW69303.1	hypothetical protein Poli38472_001459 [Pythium oligandrum]	23.98	89.76	33.66
p-cluster316845	KUF93051.1	serine/threonine-protein kinase drkB [Phytophthora nicotianae]	26.29	73.62	32.31
p-cluster316863	TMW57601.1	hypothetical protein Poli38472_003526 [Pythium oligandrum]	25.17	67.52	55.67
p-cluster316968	TMW61341.1	hypothetical protein Poli38472_012532 [Pythium oligandrum]	45.38	34.52	62.90
p-cluster317033	TMW63881.1	hypothetical protein Poli38472_014791 [Pythium oligandrum]	33.85	89.40	14.57
p-cluster317100	TMW57841.1	hypothetical protein Poli38472_013315 [Pythium oligandrum]	44.15	51.39	56.67
p-cluster317344	TMW57737.1	hypothetical protein Poli38472_014340 [Pythium oligandrum]	31.45	53.36	21.17
p-cluster317407	TMW59273.1	hypothetical protein Poli38472_004342 [Pythium oligandrum]	54.17	43.64	98.35
p-cluster317543	HIN78882.1	hypothetical protein [Gemmatimonadetes bacterium]	47.75	40.00	85.38
p-cluster317588	TMW68262.1	hypothetical protein Poli38472_005730 [Pythium oligandrum]	55.38	33.05	33.74
p-cluster317599	TMW65456.1	hypothetical protein Poli38472_008098 [Pythium oligandrum]	60.64	27.01	47.69
p-cluster317653	RLN49532.1	hypothetical protein BBJ28_00016734, partial [Nothophytophthora sp. Chile5]	40.91	68.42	46.81
p-cluster317684	CAD2196552.1	unnamed protein product [Meloidogyne enterolobii]	33.33	77.85	23.71
p-cluster317731	GAX93857.1	sulfatase-like protein [Pythium insidiosum]	76.19	15.91	5.73
p-cluster317765	TMW57365.1	hypothetical protein Poli38472_003290 [Pythium oligandrum]	33.08	77.30	19.74
p-cluster317794	TMW58471.1	hypothetical protein Poli38472_010030 [Pythium oligandrum]	61.63	44.25	96.74
p-cluster317807	GAX94157.1	lipase, putative [Pythium insidiosum]	97.56	100.00	9.48
p-cluster317812	WP_192334169.1	retention module-containing protein [Comamonas sp. CMM01]	52.75	30.64	12.52
p-cluster317886	KAF1318164.1	Fumarate hydratase, mitochondrial, partial [Globisporangium splendens]	47.62	62.05	8.14
p-cluster317904	TMW62926.1	hypothetical protein Poli38472_005544 [Pythium oligandrum]	26.36	77.01	82.84
p-cluster317929	TMW58480.1	hypothetical protein Poli38472_010039 [Pythium oligandrum]	72.00	27.62	96.97
p-cluster317983	KAG7391393.1	hypothetical protein PHYPSEUDO_004928 [Phytophthora pseudosyringae]	24.14	61.50	59.01
p-cluster318011	TMW68343.1	hypothetical protein Poli38472_005811 [Pythium oligandrum]	70.22	45.05	99.77
p-cluster318038	TMW68096.1	hypothetical protein Poli38472_007768 [Pythium oligandrum]	41.62	30.26	78.40
p-cluster318066	RLN97074.1	hypothetical protein BBJ28_00013629 [Nothophytophthora sp. Chile5]	35.03	64.44	94.79
p-cluster318267	TMW55948.1	hypothetical protein Poli38472_008596 [Pythium oligandrum]	51.02	48.77	46.82
p-cluster318284	TMW68823.1	hypothetical protein Poli38472_006291 [Pythium oligandrum]	64.68	40.35	43.79
p-cluster318348	TMW58697.1	hypothetical protein Poli38472_010256 [Pythium oligandrum]	31.52	54.34	80.77
p-cluster318752	TMW65785.1	hypothetical protein Poli38472_008427 [Pythium oligandrum]	33.62	38.48	97.85
p-cluster318859	KAF1315487.1	reverse transcriptase, partial [Globisporangium splendens]	52.12	37.08	62.50
p-cluster318952	KAF1315710.1	Pol protein, partial [Globisporangium splendens]	33.18	21.07	36.13
p-cluster318962	TMW67803.1	hypothetical protein Poli38472_007475 [Pythium oligandrum]	42.36	32.78	85.11
p-cluster319016	XP_013330623.1	hypothetical protein T310_1993 [Rasamsonia emersonii CBS 393.64]	41.76	22.65	42.71
p-cluster319082	KAE9351730.1	hypothetical protein PR003_g4760 [Phytophthora rubi]	45.45	23.73	57.95
p-cluster319132	KAF1335486.1	hypothetical protein FI667_g1174, partial [Globisporangium splendens]	41.22	77.42	11.66
p-cluster319153	TMW60710.1	hypothetical protein Poli38472_000752 [Pythium oligandrum]	34.63	69.70	73.85
p-cluster319323	TMW66258.1	hypothetical protein Poli38472_004023 [Pythium oligandrum]	42.76	58.68	9.20
p-cluster319598	TMW56796.1	hypothetical protein Poli38472_006806 [Pythium oligandrum]	56.15	45.04	87.31
p-cluster319705	TMW62688.1	hypothetical protein Poli38472_005306 [Pythium oligandrum]	56.65	48.53	81.80
p-cluster319911	KAF1336564.1	hypothetical protein FI667_g503, partial [Globisporangium splendens]	42.45	41.32	100.00
p-cluster320026	TMW65255.1	hypothetical protein Poli38472_009422 [Pythium oligandrum]	34.17	68.05	66.19
p-cluster320031	GAY05762.1	glyoxylate reductase/hydroxypyruvate reductase, putative [Pythium insidiosum]	86.50	57.36	48.56
p-cluster320170	XP_008894973.1	hypothetical protein PPTG_04482 [Phytophthora parasitica INRA-310]	52.34	37.36	96.07
p-cluster320295	TMW69414.1	hypothetical protein Poli38472_001570 [Pythium oligandrum]	42.35	54.91	89.73
p-cluster320348	GAY02104.1	protein kinase, putative [Pythium insidiosum]	100.00	51.05	20.10
p-cluster320647	TMW60767.1	hypothetical protein Poli38472_000809 [Pythium oligandrum]	50.90	100.00	100.00
p-cluster320671	POM66025.1	Hypothetical protein PHPALM_18176 [Phytophthora palmivora var. palmivora]	53.62	29.74	41.07
p-cluster320680	GAX98756.1	mitochondrial substrate carrier family protein [Pythium insidiosum]	62.07	81.12	35.49
p-cluster320687	TMW59713.1	hypothetical protein Poli38472_004782 [Pythium oligandrum]	55.22	49.03	99.25
p-cluster320718	GAX92554.1	COPII GTPase subunit SAR1 [Pythium insidiosum]	98.75	36.53	83.77
p-cluster320769	TMW56916.1	hypothetical protein Poli38472_002841 [Pythium oligandrum]	67.71	49.74	100.00
p-cluster321022	TMW68231.1	hypothetical protein Poli38472_007903 [Pythium oligandrum]	70.76	26.46	100.00
p-cluster321264	GAX94046.1	transmembrane protein putative [Pythium insidiosum]	100.00	97.30	35.29
p-cluster321398	GAY05861.1	leucine rich repeat protein [Pythium insidiosum]	85.83	97.91	39.33
p-cluster321488	ETO59307.1	hypothetical protein F444_22322, partial [Phytophthora parasitica P1976]	36.69	50.38	66.50
p-cluster321529	GAY03674.1	inorganic pyrophosphatase, putative [Pythium insidiosum]	99.64	37.72	100.00
p-cluster321582	KAF1782486.1	hypothetical protein GQ600_12246 [Phytophthora cactorum]	32.14	60.68	97.68
p-cluster321632	TMW59757.1	hypothetical protein Poli38472_004826 [Pythium oligandrum]	41.67	43.32	32.33

p-cluster321747	TMW65787.1	hypothetical protein Poli38472_008429 [Pythium oligandrum]	34.66	65.26	54.57
p-cluster321801	GAX92391.1	myb-like DNA-binding protein, putative [Pythium insidiosum]	99.09	46.71	100.00
p-cluster322120	RLN58853.1	hypothetical protein BBJ28_00010774 [Nothophytophthora sp. Chile5]	46.06	28.43	49.56
p-cluster322122	TMW65120.1	hypothetical protein Poli38472_009287 [Pythium oligandrum]	37.02	50.71	42.42
p-cluster322387	TMW65536.1	hypothetical protein Poli38472_008178 [Pythium oligandrum]	37.03	59.25	71.00
p-cluster322528	XP_012207253.1	hypothetical protein SPRG_12218 [Saprolegnia parasitica CBS 223.65]	34.33	15.24	48.35
p-cluster322555	TMW58599.1	hypothetical protein Poli38472_010158 [Pythium oligandrum]	27.40	72.18	81.93
p-cluster322598	KAF1319054.1	reverse transcriptase, partial [Globisporangium splendens]	34.15	50.28	54.71
p-cluster322679	KUF78499.1	Sperm-tail PG-rich repeat-containing protein 2 [Phytophthora nicotianae]	49.20	70.79	84.65
p-cluster322716	TMW66771.1	hypothetical protein Poli38472_014083 [Pythium oligandrum]	28.07	36.78	81.71
p-cluster322786	TMW55218.1	hypothetical protein Poli38472_013109 [Pythium oligandrum]	63.04	23.86	91.87
p-cluster322813	TMW69640.1	hypothetical protein Poli38472_001796 [Pythium oligandrum]	91.04	35.18	96.81
p-cluster322973	TMW61441.1	hypothetical protein Poli38472_012632 [Pythium oligandrum]	70.59	17.15	44.25
p-cluster322976	TMW61445.1	hypothetical protein Poli38472_012636 [Pythium oligandrum]	68.67	35.67	100.00
p-cluster322981	TMW61479.1	hypothetical protein Poli38472_012670 [Pythium oligandrum]	54.50	43.52	84.47
p-cluster322995	TMW65739.1	hypothetical protein Poli38472_008381 [Pythium oligandrum]	84.08	48.54	85.40
p-cluster323120	CAE7469296.1	unnamed protein product [Symbiodinium pilosum]	38.08	84.10	81.53
p-cluster323131	TMW67669.1	hypothetical protein Poli38472_011289 [Pythium oligandrum]	47.80	36.04	58.41
p-cluster323640	TMW63172.1	hypothetical protein Poli38472_002113 [Pythium oligandrum]	71.78	30.93	90.42
p-cluster323662	TMW60617.1	hypothetical protein Poli38472_000659 [Pythium oligandrum]	47.04	40.35	99.66
p-cluster323673	TMW60606.1	hypothetical protein Poli38472_000648 [Pythium oligandrum]	77.13	17.57	67.77
p-cluster323702	TMW64157.1	hypothetical protein Poli38472_014274 [Pythium oligandrum]	46.74	50.87	63.09
p-cluster323750	CEJ81816.1	hypothetical protein VHEMI01926 [[Torrubiella] hemipterigena]	34.21	37.31	88.17
p-cluster323794	XP_024586380.1	uncharacterized protein PHALS_07741 [Plasmopara halstedii]	40.55	40.35	47.30
p-cluster323961	KAF1315734.1	hypothetical protein FI667_g15840, partial [Globisporangium splendens]	22.76	56.44	13.80
p-cluster324077	TMW57852.1	hypothetical protein Poli38472_013326 [Pythium oligandrum]	77.67	38.13	84.20
p-cluster324121	TYZ61674.1	hypothetical protein PybrP1_010673 [[Pythium] brassicae (nom. inval.)]	50.00	52.80	15.38
p-cluster324498	GAY02711.1	replication protein [Pythium insidiosum]	100.00	36.08	85.98
p-cluster324726	GAY05927.1	Ribosomal protein S23 putative [Pythium insidiosum]	100.00	13.66	91.72
p-cluster324836	GAY04598.1	tubulin/FtsZ family [Pythium insidiosum]	92.97	34.55	99.53
p-cluster324861	KAE8968253.1	hypothetical protein PR002_g27812 [Phytophthora rubi]	41.10	45.09	91.10
p-cluster324929	KAF1330747.1	Scp-like extracellular protein, partial [Globisporangium splendens]	43.10	58.01	95.52
p-cluster324969	WP_090320517.1	leucine-rich repeat domain-containing protein [Nitrosomonas oligotropha]	32.32	46.87	26.42
p-cluster325164	KAF1328250.1	Lhy protein, partial [Globisporangium splendens]	45.93	15.61	39.18
p-cluster325301	TMW57620.1	hypothetical protein Poli38472_003545 [Pythium oligandrum]	60.00	40.85	50.00
p-cluster325319	TMW64137.1	hypothetical protein Poli38472_014254 [Pythium oligandrum]	41.36	50.52	95.99
p-cluster325365	TMW56761.1	hypothetical protein Poli38472_006771 [Pythium oligandrum]	69.41	44.59	98.26
p-cluster325366	TDH67035.1	hypothetical protein CCR75_005281 [Bremia lactucae]	45.40	67.62	25.80
p-cluster325586	TMW65643.1	hypothetical protein Poli38472_008285 [Pythium oligandrum]	55.90	34.87	99.16
p-cluster325605	TMW67817.1	hypothetical protein Poli38472_007489 [Pythium oligandrum]	40.87	60.65	96.07
p-cluster325735	GAX96833.1	2Fe-2S ferredoxin, putative [Pythium insidiosum]	98.21	49.12	100.00
p-cluster325752	GAY05747.1	replication protein [Pythium insidiosum]	33.63	37.63	39.41
p-cluster325819	TMW63257.1	hypothetical protein Poli38472_002198 [Pythium oligandrum]	44.86	54.43	100.00
p-cluster326032	TMW56530.1	hypothetical protein Poli38472_006540 [Pythium oligandrum]	28.53	56.46	90.80
p-cluster326060	GAX94263.1	TKL protein kinase [Pythium insidiosum]	92.31	77.50	14.62
p-cluster326081	KAF1318034.1	hypothetical protein FI667_g14263, partial [Globisporangium splendens]	47.83	41.01	18.11
p-cluster326130	TMW62243.1	hypothetical protein Poli38472_009736 [Pythium oligandrum]	29.30	61.20	93.36
p-cluster326190	KAI3656794.1	hypothetical protein MP638_004171, partial [Amoeboaphelidium occidentale]	37.93	37.62	73.97
p-cluster326343	TMW62696.1	hypothetical protein Poli38472_005314 [Pythium oligandrum]	40.34	40.14	24.33
p-cluster326349	TMW62713.1	hypothetical protein Poli38472_005331 [Pythium oligandrum]	46.98	33.46	47.72
p-cluster326368	TMW61989.1	hypothetical protein Poli38472_009482 [Pythium oligandrum]	49.47	58.70	25.11
p-cluster326394	XP_009845798.1	hypothetical protein H257_18442 [Aphanomyces astaci]	45.09	10.70	90.10
p-cluster326586	CAB1097217.1	unnamed protein product [Ectocarpus sp. CCAP 1310/34]	26.95	86.17	1.40
p-cluster326908	TMW55566.1	hypothetical protein Poli38472_010448 [Pythium oligandrum]	50.97	26.24	71.94
p-cluster326993	KAG1694207.1	hypothetical protein DVH05_021863 [Phytophthora capsici]	23.52	63.31	48.45
p-cluster327001	TMW59054.1	hypothetical protein Poli38472_007199 [Pythium oligandrum]	56.73	41.62	90.66
p-cluster327004	TMW58628.1	hypothetical protein Poli38472_010187 [Pythium oligandrum]	30.32	44.98	14.85
p-cluster327051	GAY00889.1	centrosome-associated protein, putative [Pythium insidiosum]	67.25	73.36	28.45
p-cluster327099	TMW67389.1	hypothetical protein Poli38472_011009 [Pythium oligandrum]	45.85	34.57	60.75
p-cluster327312	KAE8878500.1	hypothetical protein PF003_g37346 [Phytophthora fragariae]	46.89	92.48	31.00
p-cluster327396	TMW55299.1	hypothetical protein Poli38472_013190 [Pythium oligandrum]	64.56	40.65	100.00
p-cluster327419	TMW61209.1	hypothetical protein Poli38472_013672 [Pythium oligandrum]	27.52	39.86	53.88

p-cluster327538	KAF1322448.1	hypothetical protein FI667_g11173, partial [Globisporangium splendens]	54.50	41.27	98.92
p-cluster327721	GAX97649.1	charged multivesicular body protein 2a [Pythium insidiosum]	99.57	48.76	100.00
p-cluster327779	ETI34159.1	hypothetical protein F443_19290 [Phytophthora parasitica P1569]	57.38	52.55	87.56
p-cluster327833	GAY03794.1	F-box protein, putative [Pythium insidiosum]	89.33	87.21	19.84
p-cluster328061	KAF1328454.1	hypothetical protein FI667_g6865, partial [Globisporangium splendens]	46.46	45.24	45.54
p-cluster328071	TMW65305.1	hypothetical protein Poli38472_007947 [Pythium oligandrum]	52.66	34.10	28.63
p-cluster328123	TMW66085.1	hypothetical protein Poli38472_003850 [Pythium oligandrum]	49.29	38.67	39.70
p-cluster328185	GAY04101.1	gastricsin [Pythium insidiosum]	75.61	9.38	15.47
p-cluster328239	XP_024580671.1	uncharacterized protein PHALS_00671 [Plasmopara halstedii]	32.56	43.43	29.72
p-cluster328469	POM74496.1	Hypothetical protein PHPALM_8533 [Phytophthora palmivora var. palmivora]	31.26	69.42	95.17
p-cluster328655	TMW57324.1	hypothetical protein Poli38472_003249 [Pythium oligandrum]	27.42	69.79	88.81
p-cluster328729	GAY00460.1	serine protease family S33, putative [Pythium insidiosum]	80.53	97.84	36.20
p-cluster328855	TMW60256.1	hypothetical protein Poli38472_000298 [Pythium oligandrum]	60.42	29.63	5.71
p-cluster328869	TMW68867.1	hypothetical protein Poli38472_001023 [Pythium oligandrum]	41.27	78.23	88.43
p-cluster328926	TYZ61834.1	hypothetical protein PybrP1_000946 [[Pythium] brassicae (nom. inval.)]	61.54	16.16	26.95
p-cluster329059	XP_012197280.1	hypothetical protein SPRG_03313 [Saprolegnia parasitica CBS 223.65]	46.24	30.29	46.59
p-cluster329235	TMW64072.1	hypothetical protein Poli38472_014189 [Pythium oligandrum]	41.91	32.02	76.92
p-cluster329330	TMW55178.1	hypothetical protein Poli38472_013940 [Pythium oligandrum]	32.69	92.77	96.30
p-cluster329337	GAY03407.1	phosphatidylinositol 3 and 4-kinase-like protein, partial [Pythium insidiosum]	91.94	13.72	7.79
p-cluster329464	TMW64387.1	hypothetical protein Poli38472_013009 [Pythium oligandrum]	48.63	49.65	22.12
p-cluster329482	XP_009527062.1	hypothetical protein PHYSODRAFT_331892 [Phytophthora sojae]	32.43	47.84	24.94
p-cluster329539	GAY01107.1	dynein light chain [Pythium insidiosum]	100.00	26.32	18.39
p-cluster329546	GAY01113.1	DNA polymerase delta catalytic subunit [Pythium insidiosum]	99.49	24.94	98.50
p-cluster329709	TMW59458.1	hypothetical protein Poli38472_004527 [Pythium oligandrum]	46.42	85.70	82.91
p-cluster329713	GAY02610.1	GPI-anchored leucine-rich lipoprotein [Pythium insidiosum]	87.59	43.89	92.36
p-cluster329766	GAY04393.1	mannosyl-oligosaccharide alpha-1,2-mannosidase, putative [Pythium insidiosum]	81.77	66.44	16.72
p-cluster329952	TMW63566.1	hypothetical protein Poli38472_002507 [Pythium oligandrum]	79.10	28.49	63.54
p-cluster329954	TMW63568.1	hypothetical protein Poli38472_002509 [Pythium oligandrum]	68.42	27.57	27.62
p-cluster330068	TMW57737.1	hypothetical protein Poli38472_014340 [Pythium oligandrum]	26.15	77.91	40.53
p-cluster330121	TMW56237.1	hypothetical protein Poli38472_008885 [Pythium oligandrum]	37.40	97.28	99.02
p-cluster330145	TMW66219.1	hypothetical protein Poli38472_003984 [Pythium oligandrum]	61.88	48.97	56.44
p-cluster330199	GAX94126.1	pre-mRNA polyadenylation factor, putative, partial [Pythium insidiosum]	81.88	43.75	73.09
p-cluster330203	XP_009844223.1	hypothetical protein H257_17218 [Aphanomyces astaci]	47.83	19.08	44.97
p-cluster330222	TMW68678.1	hypothetical protein Poli38472_006146 [Pythium oligandrum]	37.83	90.55	23.45
p-cluster330255	TMW61062.1	hypothetical protein Poli38472_014523 [Pythium oligandrum]	60.54	28.41	98.15
p-cluster330291	KAF1332625.1	hypothetical protein FI667_g3589, partial [Globisporangium splendens]	43.06	13.83	75.79
p-cluster330339	XP_009534332.1	hypothetical protein PHYSODRAFT_523336 [Phytophthora sojae]	30.11	65.30	20.95
p-cluster330694	TMW57192.1	hypothetical protein Poli38472_003117 [Pythium oligandrum]	44.40	96.51	97.67
p-cluster330909	KAH7509569.1	hypothetical protein KRP22_1069 [Phytophthora ramorum]	44.23	62.67	4.28
p-cluster330978	GAY01393.1	ATP-dependent chaperone ClpB [Pythium insidiosum]	97.93	49.61	99.74
p-cluster331147	TYZ60874.1	hypothetical protein PybrP1_001446 [[Pythium] brassicae (nom. inval.)]	49.35	55.64	6.75
p-cluster331219	KAF4148815.1	hypothetical protein GN958_ATG02013 [Phytophthora infestans]	28.14	99.65	31.47
p-cluster331240	TMW55564.1	hypothetical protein Poli38472_010446 [Pythium oligandrum]	59.31	44.39	93.77
p-cluster331385	TMW63233.1	hypothetical protein Poli38472_002174 [Pythium oligandrum]	30.07	97.22	97.14
p-cluster331400	KAE9063360.1	hypothetical protein PF010_g29023 [Phytophthora fragariae]	35.76	16.83	81.18
p-cluster331407	KAG7389577.1	hypothetical protein PHYBOEH_007414 [Phytophthora boehmeriae]	42.37	43.26	71.86
p-cluster331497	TMW58121.1	hypothetical protein Poli38472_011709 [Pythium oligandrum]	38.30	50.14	96.47
p-cluster331544	TMW55196.1	hypothetical protein Poli38472_013087 [Pythium oligandrum]	88.69	26.79	98.21
p-cluster331614	KAE9052009.1	hypothetical protein PR001_g915 [Phytophthora rubi]	44.55	27.64	30.25
p-cluster332019	TMW55989.1	hypothetical protein Poli38472_008637 [Pythium oligandrum]	42.56	62.98	94.64
p-cluster332078	TMW61954.1	hypothetical protein Poli38472_009447 [Pythium oligandrum]	61.66	43.04	100.00
p-cluster332335	KAF4037588.1	CCCH-type zinc finger [Phytophthora infestans]	37.67	81.92	88.98
p-cluster332377	GAX93164.1	histone methylation protein DOT1, putative [Pythium insidiosum]	82.09	51.94	35.08
p-cluster332409	TMW67373.1	hypothetical protein Poli38472_012489 [Pythium oligandrum]	76.92	8.64	19.92
p-cluster332430	TMW55473.1	hypothetical protein Poli38472_010355 [Pythium oligandrum]	59.59	32.77	97.29
p-cluster332565	TMW63653.1	hypothetical protein Poli38472_002594 [Pythium oligandrum]	50.65	23.58	19.83
p-cluster332737	TMW60798.1	hypothetical protein Poli38472_000840 [Pythium oligandrum]	75.00	34.29	23.12
p-cluster332743	KAG2774714.1	hypothetical protein Pcac1_g14466 [Phytophthora cactorum]	57.14	30.77	12.88
p-cluster332777	RLN74237.1	hypothetical protein BBJ28_00000600 [Nothophytophthora sp. Chile5]	47.73	32.82	45.36
p-cluster332807	TMW64877.1	hypothetical protein Poli38472_009044 [Pythium oligandrum]	52.63	49.67	7.06
p-cluster332812	TYZ64128.1	hypothetical protein PybrP1_001652 [[Pythium] brassicae (nom. inval.)]	23.71	96.53	10.40
p-cluster332910	GAY02518.1	elicitor-like transglutaminase [Pythium insidiosum]	91.76	100.00	10.61

p-cluster332980	TMW55659.1	hypothetical protein Poli38472_010541 [Pythium oligandrum]	38.51	41.24	91.46
p-cluster333145	TMW69476.1	hypothetical protein Poli38472_001632 [Pythium oligandrum]	55.94	22.24	87.20
p-cluster333243	RLN88144.1	hypothetical protein BBJ28_00022437 [Nothophytophthora sp. Chile5]	31.56	80.99	21.51
p-cluster333356	TMW56655.1	hypothetical protein Poli38472_006665 [Pythium oligandrum]	44.83	28.03	28.02
p-cluster333380	GAY06300.1	DNA-binding protein RHL1-like protein [Pythium insidiosum]	74.44	91.36	35.32
p-cluster333398	TMW63398.1	hypothetical protein Poli38472_002339 [Pythium oligandrum]	26.19	73.48	20.30
p-cluster333411	KAF1328096.1	Ring finger protein nhl-1, partial [Globisporangium splendens]	45.16	33.58	15.64
p-cluster333510	TMW68375.1	hypothetical protein Poli38472_005843 [Pythium oligandrum]	48.15	76.06	22.22
p-cluster333539	GAX99639.1	autophagy-like protein 8 [Pythium insidiosum]	95.12	46.95	99.15
p-cluster333543	KAH9264525.1	hypothetical protein BASA83_011996 [Batrachochytrium salamandrivorans]	29.82	28.89	57.09
p-cluster333601	TMW58495.1	hypothetical protein Poli38472_010054 [Pythium oligandrum]	59.48	44.61	50.00
p-cluster333686	TMW61207.1	hypothetical protein Poli38472_013670 [Pythium oligandrum]	40.00	87.84	75.32
p-cluster333688	ETI29765.1	hypothetical protein F443_23119, partial [Phytophthora parasitica P1569]	41.94	17.42	43.06
p-cluster333707	TMW60135.1	hypothetical protein Poli38472_000177 [Pythium oligandrum]	65.85	44.97	100.00
p-cluster333891	TMW59141.1	hypothetical protein Poli38472_007286 [Pythium oligandrum]	33.33	56.31	44.57
p-cluster333935	TMW57596.1	hypothetical protein Poli38472_003521 [Pythium oligandrum]	37.50	65.79	6.19
p-cluster333938	KAE8990206.1	hypothetical protein PR002_g21217 [Phytophthora rubi]	45.00	32.97	17.19
p-cluster333966	TMW55679.1	hypothetical protein Poli38472_010561 [Pythium oligandrum]	50.77	45.32	12.08
p-cluster334092	TMW62303.1	hypothetical protein Poli38472_009796 [Pythium oligandrum]	63.80	40.75	99.54
p-cluster334756	KAE9345656.1	hypothetical protein PF008_g8640 [Phytophthora fragariae]	35.36	14.51	22.85
p-cluster334779	TMW63235.1	hypothetical protein Poli38472_002176 [Pythium oligandrum]	36.33	60.14	29.69
p-cluster335165	OWZ21837.1	RNA (guanine-9-)-methyltransferase [Phytophthora megakarya]	57.94	78.52	40.38
p-cluster335285	TDH68553.1	hypothetical protein CCR75_000293 [Bremia lactucae]	40.17	94.96	41.92
p-cluster335307	TMW55951.1	hypothetical protein Poli38472_008599 [Pythium oligandrum]	52.47	33.85	36.30
p-cluster335636	KUF76044.1	Coiled-coil domain-containing protein [Phytophthora nicotianae]	50.54	84.47	53.29
p-cluster336302	CCA17226.1	conserved hypothetical protein [Albugo laibachii Nc14]	28.14	58.82	61.51
p-cluster336332	TMW67825.1	hypothetical protein Poli38472_007497 [Pythium oligandrum]	61.27	29.77	75.00
p-cluster336463	GAX95999.1	exonuclease 1, putative, partial [Pythium insidiosum]	100.00	31.07	24.19
p-cluster336490	KAF1329266.1	hypothetical protein FI667_g6005, partial [Globisporangium splendens]	33.71	28.03	22.02
p-cluster336498	GAY04700.1	chromatin assembly factor 1 subunit B-like protein [Pythium insidiosum]	96.22	90.24	29.32
p-cluster336679	TMW61205.1	hypothetical protein Poli38472_013668 [Pythium oligandrum]	30.98	54.22	24.12
p-cluster336743	TMW67314.1	hypothetical protein Poli38472_012430 [Pythium oligandrum]	31.43	70.04	64.81
p-cluster337011	CAG8510172.1	16020_t:CDS:2 [Funneliformis caledonium]	50.47	37.68	11.05
p-cluster337230	TMW55696.1	hypothetical protein Poli38472_010578 [Pythium oligandrum]	37.13	66.31	90.42
p-cluster337375	TMW56643.1	hypothetical protein Poli38472_006653 [Pythium oligandrum]	59.65	28.08	17.70
p-cluster337826	GAX98005.1	seryl-tRNA synthetase [Pythium insidiosum]	87.85	44.34	98.94
p-cluster337922	TMW58276.1	hypothetical protein Poli38472_011864 [Pythium oligandrum]	81.39	45.83	92.01
p-cluster338261	KAF0730094.1	hypothetical protein Ae201684_012390 [Aphanomyces euteiches]	51.78	29.50	96.26
p-cluster338428	OQS04262.1	Crinkler (CRN) family protein [Thraustotheca clavata]	84.62	46.43	11.71
p-cluster338733	TMW69842.1	hypothetical protein Poli38472_001998 [Pythium oligandrum]	74.65	32.27	46.41
p-cluster339123	TMW57152.1	hypothetical protein Poli38472_003077 [Pythium oligandrum]	25.00	56.18	98.11
p-cluster339143	TMW58168.1	hypothetical protein Poli38472_011756 [Pythium oligandrum]	52.08	34.34	54.03
p-cluster339226	TMW57092.1	hypothetical protein Poli38472_003017 [Pythium oligandrum]	57.58	65.00	8.88
p-cluster339527	TMW68857.1	hypothetical protein Poli38472_001013 [Pythium oligandrum]	66.20	42.07	83.91
p-cluster339978	KAG7388086.1	hypothetical protein PHYBOEH_008011 [Phytophthora boehmeriae]	42.74	84.23	87.23
p-cluster340696	POM67973.1	Hypothetical protein PHPALM_15923 [Phytophthora palmivora var. palmivora]	56.38	5.57	41.23
p-cluster341603	GAX98550.1	putative methyltransferase METT10D [Pythium insidiosum]	54.26	85.43	26.83
p-cluster342914	TMW62596.1	hypothetical protein Poli38472_005214 [Pythium oligandrum]	33.69	96.81	46.27
p-cluster343160	TMW65804.1	hypothetical protein Poli38472_003569 [Pythium oligandrum]	51.56	52.50	18.18
p-cluster343376	GAX92779.1	homeodomain transcription factor, putative [Pythium insidiosum]	68.92	57.81	13.03
p-cluster343407	XP_009828220.1	DNA topoisomerase VI, B subunit, variant [Aphanomyces astaci]	59.11	99.02	26.27
p-cluster343433	KAE8983593.1	hypothetical protein PR001_g23409 [Phytophthora rubi]	59.62	88.14	41.27
p-cluster343629	KAE9167965.1	hypothetical protein PF002_g30739, partial [Phytophthora fragariae]	25.74	97.27	54.13
p-cluster344080	GAY02875.1	exosome complex exonuclease RRP45-like protein [Pythium insidiosum]	96.46	100.00	22.97
p-cluster344529	TMW62212.1	hypothetical protein Poli38472_009705 [Pythium oligandrum]	69.97	32.64	83.92
p-cluster344807	RQM27221.1	hypothetical protein B5M09_009506 [Aphanomyces astaci]	25.26	83.05	76.77
p-cluster344976	GAY05747.1	replication protein [Pythium insidiosum]	38.49	44.26	50.09
p-cluster344982	RMX62774.1	hypothetical protein DD238_007334 [Peronospora effusa]	55.68	84.85	14.18
p-cluster345730	RLN44037.1	hypothetical protein BBJ28_00024823 [Nothophytophthora sp. Chile5]	34.53	19.94	27.70
p-cluster346261	KAE9317214.1	hypothetical protein PR003_g18528 [Phytophthora rubi]	33.33	50.68	15.77
p-cluster346505	GAX92592.1	phox-like domain containing protein [Pythium insidiosum]	75.59	30.87	29.11
p-cluster346777	KAF1321113.1	hypothetical protein FI667_g12129, partial [Globisporangium splendens]	31.34	71.43	5.71

p-cluster346852	GAY03291.1	phosphoethanolamine N-methyltransferase, putative [Pythium insidiosum]	63.74	82.85	8.42
p-cluster346898	ETO64182.1	hypothetical protein F444_18275, partial [Phytophthora parasitica P1976]	38.70	80.74	82.18
p-cluster347074	RLN73590.1	hypothetical protein BBJ28_00023107 [Nothophytophthora sp. Chile5]	48.89	33.71	9.50
p-cluster347306	KAF1324561.1	Udp-n-acetylglucosamine--peptide n-acetylglucosaminyltransferase sec, partial [Globisporangium splendens]	51.69	100.00	8.69
p-cluster347402	GAX97649.1	charged multivesicular body protein 2a [Pythium insidiosum]	96.51	93.48	36.60
p-cluster347515	KOO35698.1	hypothetical protein Ctob_016172, partial [Chrysochromulina tobinii]	30.91	52.03	50.19
p-cluster347583	GAX95033.1	TRAF3-interacting protein, putative [Pythium insidiosum]	91.71	57.37	24.01
p-cluster347937	XP_008610175.1	hypothetical protein SDRG_06189 [Saprolegnia diclina VS20]	44.40	100.00	36.89
p-cluster348091	GAX92992.1	RNA polymerase-associated protein CTR9 [Pythium insidiosum]	60.87	100.00	7.72
p-cluster348308	TMW66234.1	hypothetical protein Poli38472_003999 [Pythium oligandrum]	37.34	55.54	20.92
p-cluster348325	RQM27595.1	hypothetical protein B5M09_010155 [Aphanomyces astaci]	38.10	79.62	96.04
p-cluster348433	TMW60355.1	hypothetical protein Poli38472_000397 [Pythium oligandrum]	43.94	41.18	24.66
p-cluster348474	GAX98228.1	CMGC/MAPK protein kinase [Pythium insidiosum]	81.59	100.00	28.29
p-cluster348826	KAF1328042.1	hypothetical protein FI667_g7374, partial [Globisporangium splendens]	56.41	37.32	45.22
p-cluster349066	TMW60484.1	hypothetical protein Poli38472_000526 [Pythium oligandrum]	38.04	93.88	27.76
p-cluster349221	GAX95191.1	glycosylltransferase-like domain-containing protein 1-like protein [Pythium insidiosum]	81.53	94.37	23.13
p-cluster349291	GAX97319.1	V-type proton ATPase subunit H, putative [Pythium insidiosum]	68.24	100.00	25.49
p-cluster349333	GAY04039.1	CAMKK protein kinase [Pythium insidiosum]	67.68	94.29	5.36
p-cluster349411	TMW55887.1	hypothetical protein Poli38472_008535 [Pythium oligandrum]	60.63	62.82	17.91
p-cluster349533	TMW56366.1	hypothetical protein Poli38472_006376 [Pythium oligandrum]	31.94	98.66	37.52
p-cluster349931	TMW66876.1	hypothetical protein Poli38472_011992 [Pythium oligandrum]	32.99	77.85	27.25
p-cluster350032	TMW66819.1	hypothetical protein Poli38472_011935 [Pythium oligandrum]	40.28	85.57	10.35
p-cluster350070	GAY00967.1	DNA-binding protein, 42 kDa [Pythium insidiosum]	94.12	60.49	12.91
p-cluster350375	GAY02207.1	STE/STE11 protein kinase [Pythium insidiosum]	84.00	100.00	13.66
p-cluster350388	GAY06700.1	replication protein [Pythium insidiosum]	74.45	95.13	50.13
p-cluster350419	TMW69151.1	hypothetical protein Poli38472_001307 [Pythium oligandrum]	36.21	53.43	46.70
p-cluster350818	TMW64732.1	hypothetical protein Poli38472_011612 [Pythium oligandrum]	32.49	92.31	13.35
p-cluster351204	GAX92617.1	trans-acting enoyl reductase, putative [Pythium insidiosum]	61.19	83.75	5.35
p-cluster351384	GAX96101.1	glucosidase, putative, partial [Pythium insidiosum]	77.91	78.10	6.25
p-cluster351546	QTZ83151.1	MAG: capsid protein [Emberiza pusilla parvo-like hybrid virus]	38.06	86.21	39.62
p-cluster351564	RLN86915.1	hypothetical protein BBJ28_00007391 [Nothophytophthora sp. Chile5]	32.30	99.28	14.35
p-cluster351623	GAX94671.1	HECT ubiquitin ligase, putative [Pythium insidiosum]	99.54	100.00	20.44
p-cluster352882	GAX92657.1	serine protease family S33, putative [Pythium insidiosum]	82.35	100.00	5.06
p-cluster353116	TMW68157.1	hypothetical protein Poli38472_007829 [Pythium oligandrum]	57.03	100.00	18.02
p-cluster353238	GAX92992.1	RNA polymerase-associated protein CTR9 [Pythium insidiosum]	98.45	96.99	11.45
p-cluster353277	GAX97677.1	eukaryotic translation initiation factor 5B [Pythium insidiosum]	82.79	100.00	12.63
p-cluster353290	KAG3117558.1	hypothetical protein PI125_g3650 [Phytophthora idaei]	60.34	92.06	18.67
p-cluster353339	TMW60707.1	hypothetical protein Poli38472_000749 [Pythium oligandrum]	35.61	99.20	5.29
p-cluster353831	TMW64805.1	hypothetical protein Poli38472_008972 [Pythium oligandrum]	32.40	70.23	34.96
p-cluster354193	TMW58892.1	hypothetical protein Poli38472_007037 [Pythium oligandrum]	55.63	29.05	91.95
p-cluster354370	TMW64557.1	hypothetical protein Poli38472_011437 [Pythium oligandrum]	32.65	61.46	58.44
p-cluster354979	GAX97439.1	vacuolar protein sortingassociated protein VTA1 put, partial [Pythium insidiosum]	57.39	100.00	28.30
p-cluster354991	TMW66084.1	hypothetical protein Poli38472_003849 [Pythium oligandrum]	60.38	20.62	3.97
p-cluster355298	GAY01252.1	transmembrane protein, putative [Pythium insidiosum]	76.00	21.61	39.11
p-cluster356455	KAF1321521.1	Tkl protein kinase, partial [Globisporangium splendens]	43.32	39.07	47.09
p-cluster357064	GAX98664.1	transmembrane protein, putative [Pythium insidiosum]	75.51	56.65	22.72
p-cluster357191	RLN74125.1	hypothetical protein BBJ28_00013297 [Nothophytophthora sp. Chile5]	74.07	40.60	2.51
p-cluster357293	KAG4245600.1	hypothetical protein PC116_g6624 [Phytophthora cactorum]	43.05	47.71	99.34
p-cluster357403	TMW62295.1	hypothetical protein Poli38472_009788 [Pythium oligandrum]	37.74	67.86	37.69
p-cluster358351	TMW63799.1	hypothetical protein Poli38472_002740 [Pythium oligandrum]	68.96	25.53	55.90
p-cluster358605	GAY00742.1	epsin-like protein [Pythium insidiosum]	95.89	48.03	27.65
p-cluster358701	TMW68414.1	hypothetical protein Poli38472_005882 [Pythium oligandrum]	47.52	78.50	72.88
p-cluster359248	KAG3082942.1	hypothetical protein PI125_g19859 [Phytophthora idaei]	52.48	25.00	14.22
p-cluster360110	GAY00538.1	A/G-specific adenine glycosyllase [Pythium insidiosum]	61.14	100.00	15.12
p-cluster360187	GAX96588.1	multiple inositol polyphosphate phosphatase, putative [Pythium insidiosum]	77.03	8.85	29.03
p-cluster360419	TMW59272.1	hypothetical protein Poli38472_004341 [Pythium oligandrum]	41.73	80.00	50.19
p-cluster360542	TMW65290.1	hypothetical protein Poli38472_007932 [Pythium oligandrum]	50.34	47.58	66.67
p-cluster360689	TMW57977.1	hypothetical protein Poli38472_013451 [Pythium oligandrum]	59.17	33.07	47.18
p-cluster361036	GAX99420.1	catalase [Pythium insidiosum]	89.16	30.62	82.63
p-cluster361207	TMW63131.1	hypothetical protein Poli38472_002072 [Pythium oligandrum]	81.08	30.08	10.88
p-cluster361249	XP_009828885.1	TKL protein kinase [Aphanomyces astaci]	32.36	72.42	73.44
p-cluster361406	TMW69271.1	hypothetical protein Poli38472_001427 [Pythium oligandrum]	78.01	45.19	70.85

p-cluster361421	GAY00528.1	H/ACA ribonucleoprotein complex non-core subunit NAF1, putative [Pythium insidiosum]	76.96	74.12	69.96
p-cluster361526	KAE8909473.1	hypothetical protein PF003_g6601 [Phytophthora fragariae]	31.58	50.76	43.23
p-cluster362100	TMW62245.1	hypothetical protein Poli38472_009738 [Pythium oligandrum]	39.27	27.80	35.93
p-cluster362110	TMW56766.1	hypothetical protein Poli38472_006776 [Pythium oligandrum]	34.30	77.66	15.59
p-cluster362159	GAY01449.1	splicing factor 3 subunit putative [Pythium insidiosum]	97.98	100.00	27.47
p-cluster362189	TMW65373.1	hypothetical protein Poli38472_008015 [Pythium oligandrum]	42.64	40.09	68.64
p-cluster362884	TMW65751.1	hypothetical protein Poli38472_008393 [Pythium oligandrum]	32.76	54.65	20.95
p-cluster363028	TMW64170.1	hypothetical protein Poli38472_014287 [Pythium oligandrum]	55.52	41.90	51.29
p-cluster363106	TMW59425.1	hypothetical protein Poli38472_004494 [Pythium oligandrum]	58.46	57.55	10.62
p-cluster363826	TMW62713.1	hypothetical protein Poli38472_005331 [Pythium oligandrum]	33.74	44.86	34.24
p-cluster364131	KAF6734811.1	Disks large-like protein 5 [Oryzias melastigma]	47.45	52.23	5.13
p-cluster364264	TMW64200.1	hypothetical protein Poli38472_012822 [Pythium oligandrum]	45.54	96.84	24.13
p-cluster364544	TMW61250.1	hypothetical protein Poli38472_013713 [Pythium oligandrum]	59.75	49.38	46.82
p-cluster364627	TMW69703.1	hypothetical protein Poli38472_001859 [Pythium oligandrum]	42.99	78.31	52.82
p-cluster364860	GAY06760.1	G-protein coupled receptor [Pythium insidiosum]	97.67	57.03	20.19
p-cluster365027	TMW61005.1	hypothetical protein Poli38472_014466 [Pythium oligandrum]	30.62	29.06	29.80
p-cluster365325	GAX98679.1	transcription elongation factor SPT5, putative [Pythium insidiosum]	96.65	100.00	8.26
p-cluster365374	TMW66522.1	hypothetical protein Poli38472_004287 [Pythium oligandrum]	27.96	86.24	38.54
p-cluster365772	TMW55103.1	hypothetical protein Poli38472_013865 [Pythium oligandrum]	48.51	92.94	39.78
p-cluster366174	GAX95972.1	putative vesicle coat complex COPII subunit SEC24/subunit SFB2 [Pythium insidiosum]	83.50	81.60	9.35
p-cluster366262	TMW68474.1	hypothetical protein Poli38472_005942 [Pythium oligandrum]	42.16	54.62	18.33
p-cluster366332	TMW66523.1	hypothetical protein Poli38472_004288 [Pythium oligandrum]	36.42	75.86	26.32
p-cluster366507	KAE8988953.1	hypothetical protein PR002_g21595 [Phytophthora rubi]	43.86	39.76	62.86
p-cluster366685	KAF1313803.1	G-protein coupled receptor, partial [Globisporangium splendens]	55.22	94.37	11.24
p-cluster366864	OWZ19923.1	LOW QUALITY PROTEIN: hypothetical protein PHMEG_0005748 [Phytophthora megakarya]	25.08	61.47	78.08
p-cluster366918	RLN72468.1	hypothetical protein BBJ28_00025282 [Nothophytophthora sp. Chile5]	50.47	89.78	32.27
p-cluster367464	GAX94254.1	glucosidase 2 subunit beta-like protein [Pythium insidiosum]	73.48	44.27	97.04
p-cluster368226	TMW64627.1	hypothetical protein Poli38472_011507 [Pythium oligandrum]	46.17	54.42	55.54
p-cluster368454	TMW58632.1	hypothetical protein Poli38472_010191 [Pythium oligandrum]	32.04	98.34	24.45
p-cluster368581	KAF1321493.1	hypothetical protein FI667_g11940, partial [Globisporangium splendens]	36.32	69.41	46.62
p-cluster368733	GAX99588.1	Axonemal Inner Arm I1 Intermediate Chain Dynein [Pythium insidiosum]	98.03	80.42	27.09
p-cluster369059	TMW68012.1	hypothetical protein Poli38472_007684 [Pythium oligandrum]	67.74	26.88	52.25
p-cluster369263	POM77269.1	hypothetical protein PHPALM_5372, partial [Phytophthora palmivora var. palmivora]	50.00	100.00	89.19
p-cluster369266	OQR98765.1	hypothetical protein THRCLA_21870 [Thraustotheca clavata]	25.42	51.32	48.03
p-cluster369273	TMW57385.1	hypothetical protein Poli38472_003310 [Pythium oligandrum]	41.79	79.14	23.35
p-cluster369416	XP_008869565.1	hypothetical protein, variant 1 [Aphanomyces invadans]	39.68	32.90	22.92
p-cluster369588	TMW58615.1	hypothetical protein Poli38472_010174 [Pythium oligandrum]	47.62	49.61	4.44
p-cluster369976	TMW67376.1	hypothetical protein Poli38472_012492 [Pythium oligandrum]	34.32	33.22	53.67
p-cluster370221	TMW68033.1	hypothetical protein Poli38472_007705 [Pythium oligandrum]	64.81	37.76	15.56
p-cluster370356	TMW59055.1	hypothetical protein Poli38472_007200 [Pythium oligandrum]	48.89	45.49	27.43
p-cluster370407	TMW60279.1	hypothetical protein Poli38472_000321 [Pythium oligandrum]	54.46	38.28	97.24
p-cluster370785	GAY03725.1	ABC transporter lipid exporter ABCA1 family [Pythium insidiosum]	59.26	75.65	13.59
p-cluster371269	TMW64682.1	hypothetical protein Poli38472_011562 [Pythium oligandrum]	51.00	87.39	10.92
p-cluster371728	KAE9261061.1	hypothetical protein PF008_g32942 [Phytophthora fragariae]	31.43	51.87	82.99
p-cluster371864	TMW66838.1	hypothetical protein Poli38472_011954 [Pythium oligandrum]	56.02	31.19	36.70
p-cluster372203	OWY92127.1	hypothetical protein PHMEG_00038995 [Phytophthora megakarya]	45.35	83.84	68.25
p-cluster372215	TMW69721.1	hypothetical protein Poli38472_001877 [Pythium oligandrum]	24.88	77.45	24.64
p-cluster372501	GAX92860.1	ribosomal protein S2 [Pythium insidiosum]	81.67	68.24	21.82
p-cluster372508	KAG6964218.1	hypothetical protein JG688_00007795 [Phytophthora aleatoria]	25.85	40.29	85.23
p-cluster372751	GAX96946.1	tartrate-resistant acid phosphatase type 5 [Pythium insidiosum]	61.88	100.00	35.73
p-cluster372878	QTZ83151.1	MAG: capsid protein [Emberiza pusilla parvo-like hybrid virus]	36.65	39.90	41.24
p-cluster373497	KAG2508721.1	hypothetical protein JM16_008306 [Phytophthora kernoviae]	38.18	43.65	49.30
p-cluster373513	GAY05747.1	replication protein [Pythium insidiosum]	46.18	49.21	50.09
p-cluster373603	TMW63958.1	hypothetical protein Poli38472_014663 [Pythium oligandrum]	47.55	50.72	73.40
p-cluster373748	KAE9090258.1	hypothetical protein PF010_g18656 [Phytophthora fragariae]	80.49	62.12	10.96
p-cluster373970	GAY05747.1	replication protein [Pythium insidiosum]	60.00	49.18	10.50
p-cluster374021	KAE8957867.1	hypothetical protein PR001_g31226, partial [Phytophthora rubi]	30.36	51.04	67.42
p-cluster374022	ETL99743.1	hypothetical protein L917_03449 [Phytophthora parasitica]	69.57	55.42	23.12
p-cluster374323	CAF4114896.1	unnamed protein product [Rotaria magnacalcarata]	29.95	49.00	22.47
p-cluster375485	TMW68563.1	hypothetical protein Poli38472_006031 [Pythium oligandrum]	31.53	98.29	10.73
p-cluster375783	TMW63268.1	hypothetical protein Poli38472_002209 [Pythium oligandrum]	40.23	80.89	27.73
p-cluster375816	KAG3202215.1	hypothetical protein PC129_g23307 [Phytophthora cactorum]	35.03	93.88	51.82

p-cluster376106	KAE8958858.1	hypothetical protein PR001_g30910 [Phytophthora rubi]	63.77	49.64	16.39
p-cluster377026	KAF1316114.1	Integrase catalytic core protein, partial [Globisporangium splendens]	30.38	91.19	20.40
p-cluster377088	KAF0690190.1	hypothetical protein As57867_018319 [Aphanomyces stellatus]	48.08	74.29	46.22
p-cluster377102	TMW63644.1	hypothetical protein Poli38472_002585 [Pythium oligandrum]	34.97	100.00	19.30
p-cluster377103	KAF1327825.1	Breast cancer type 2 susceptibility, partial [Globisporangium splendens]	32.32	61.90	9.47
p-cluster377122	GAY03286.1	H/ACA ribonucleoprotein complex subunit 4 [Pythium insidiosum]	87.29	86.72	24.13
p-cluster377453	RMX69642.1	hypothetical protein DD238_002237 [Peronospora effusa]	92.68	46.86	13.82
p-cluster377787	GAY06760.1	G-protein coupled receptor [Pythium insidiosum]	48.68	82.61	7.04
p-cluster378096	TMW54981.1	hypothetical protein Poli38472_014752 [Pythium oligandrum]	67.74	48.06	10.16
p-cluster378384	MCL2386855.1	stalk domain-containing protein [Defluviitaleaceae bacterium]	60.00	50.00	6.99
p-cluster378439	KAE9362126.1	hypothetical protein PF008_g367 [Phytophthora fragariae]	51.76	69.83	44.50
p-cluster378510	KAG6584586.1	hypothetical protein IUM83_07934 [Phytophthora cinnamomi]	47.85	95.29	3.60
p-cluster378650	KAE8982983.1	hypothetical protein PR001_g23574 [Phytophthora rubi]	46.00	86.34	25.30
p-cluster379195	TMW55930.1	hypothetical protein Poli38472_008578 [Pythium oligandrum]	31.54	96.90	9.04
p-cluster379249	TMW59954.1	hypothetical protein Poli38472_005023 [Pythium oligandrum]	52.94	93.33	17.68
p-cluster379403	BAS04780.1	exo-1,3-beta glucanase, partial [Pythium insidiosum]	76.85	100.00	14.75
p-cluster379421	GAY04117.1	serine protease family S33, putative [Pythium insidiosum]	52.94	62.96	3.71
p-cluster379458	CEP01103.1	hypothetical protein PBRA_008415 [Plasmodiophora brassicae]	62.86	76.09	86.42
p-cluster379616	GAX97384.1	FAD dependent oxidoreductase [Pythium insidiosum]	95.24	100.00	19.76
p-cluster383523	TMW65957.1	hypothetical protein Poli38472_003722 [Pythium oligandrum]	43.33	100.00	96.75
p-cluster388968	TMW64235.1	hypothetical protein Poli38472_012857 [Pythium oligandrum]	92.59	100.00	12.98
p-cluster409750	TMW56714.1	hypothetical protein Poli38472_006724 [Pythium oligandrum]	47.78	100.00	9.91
p-cluster461125	KAE9309726.1	hypothetical protein PR003_g20452 [Phytophthora rubi]	50.18	63.07	48.77
p-cluster463687	TMW59540.1	hypothetical protein Poli38472_004609 [Pythium oligandrum]	54.52	89.15	89.52
p-cluster486426	OQR84071.1	hypothetical protein ACHHYP_13943 [Achlya hypogyna]	32.60	86.20	83.04
p-cluster496436	TMW61321.1	hypothetical protein Poli38472_012512 [Pythium oligandrum]	40.00	71.90	70.55
p-cluster498223	KAH9118114.1	hypothetical protein AeMF1_008575, partial [Aphanomyces euteiches]	41.04	98.56	58.79
p-cluster499434	KAF1313151.1	putative retroelement, partial [Globisporangium splendens]	77.36	22.65	2.72
p-cluster500680	KAG7388086.1	hypothetical protein PHYBOEH_008011 [Phytophthora boehmeriae]	42.55	99.66	44.05
