# Supplementary material for: Comparative Genomic Analysis Reveals Gene Content Diversity, Phylogenomic Contour, Putative Virulence Determinants, and Potential Diagnostic Markers within Pythium insidiosum Traits
Source: J Fungi (Basel). 2023 Jan 27;9(2):169. doi: 10.3390/jof9020169 (PMC9962146; doi:10.3390/jof9020169)
Supplement: Supplementary file 1 [file jof-09-00169-s001.zip › 221128_Supplementary Table S3 (PDF).pdf]

Supplementary Table S3. BLAST search hits of the *P. insidiosum*-specific Core 2 gene-translated proteins against the MvirDB database.

Query gene ID	Hit ID	Hit Description [Organism]	Alignment Sequence Identity (%)	Query Alignment Coverage (%)	Hit Alignment Coverage (%)
p-cluster315039	VFID 12058	Gamma-glutamyl phosphate reductase	35.84	23.09	98.79
p-cluster324836	VFID 13367	leucine-rich repeat-containing protein [Salmonella typhimurium]	40.00	6.80	10.72
p-cluster293156	VFID 13703	unknown [Shigella flexneri]	24.31	35.17	93.89
p-cluster285703	VFID 13899	hypothetical protein [Escherichia coli]	37.65	19.00	7.17
p-cluster283019	VFID 14152	serine/threonine protein kinase PpkA [Pseudomonas aeruginosa]	31.80	20.02	19.96
p-cluster330978	VFID 15128	ClpB protein	55.85	45.51	80.68
p-cluster204558	VFID 15145	Thioredoxin	31.94	13.91	65.42
p-cluster227399	VFID 15950	cell surface protein [Bacteroides thetaiotaomicron]	37.25	12.78	22.35
p-cluster204852	VFID 15953	hypothetical protein BT_2630 [Bacteroides thetaiotaomicron]	29.64	27.62	91.77
p-cluster216792	VFID 18169	WD-40 repeat-containing protein [Nostoc sp.]	25.32	89.06	18.74
p-cluster356455	VFID 19118	protein kinase-like protein [Streptomyces coelicolor]	29.74	31.03	30.97
p-cluster205114	VFID 20214	protein kinase [Salmonella enterica]	33.33	9.97	26.33
p-cluster233975	VFID 20412	putative dehydrogenase [Salmonella enterica]	25.37	10.49	54.08
p-cluster337826	VFID 20578	seryl-tRNA synthetase [Salmonella enterica]	34.41	35.28	92.33
p-cluster207977	VFID 20579	recombination factor protein RarA [Salmonella enterica]	24.17	56.34	45.19
p-cluster235704	VFID 23251	putative trimethoprim resistance dihydrofolate reductase [Escherichia coli]	34.27	11.34	89.07
p-cluster246548	VFID 24337	multidrug efflux protein [Vibrio vulnificus]	26.19	32.09	98.90
p-cluster318952	VFID 2457	IgA-specific serine endopeptidase [Neisseria meningitidis]	27.01	1.99	9.59
p-cluster321022	VFID 25936	alpha-latrotoxin precursor [Latrodectus tredecimguttatus]	21.75	13.21	18.99
p-cluster277547	VFID 26375	thrombospondin related adhesive protein [Cryptosporidium parvum]	27.34	19.59	40.61
p-cluster314500	VFID 26400	cGMP dependent protein kinase [Toxoplasma gondii]	33.19	26.33	22.64
p-cluster249109	VFID 26400	cGMP dependent protein kinase [Toxoplasma gondii]	27.65	13.77	24.14
p-cluster305995	VFID 26447	heat shock protein 70 [Toxoplasma gondii]	91.18	18.28	5.10
p-cluster361036	VFID 26459	Peroxisomal catalase	44.16	28.93	84.66
p-cluster230530	VFID 26470	cGMP-dependent protein kinase [Eimeria tenella]	36.36	19.10	5.48
p-cluster371864	VFID 26470	cGMP-dependent protein kinase [Eimeria tenella]	34.26	17.82	9.97
p-cluster282404	VFID 26470	cGMP-dependent protein kinase [Eimeria tenella]	31.88	27.63	20.54
p-cluster317812	VFID 26599	85 kDa merozoite protein [Babesia bovis]	31.70	32.95	41.44
p-cluster324929	VFID 26605	CTRP [Plasmodium falciparum]	38.71	11.97	2.89
p-cluster309322	VFID 26605	CTRP [Plasmodium falciparum]	33.33	35.06	2.46
p-cluster326349	VFID 26643	reticulocyte binding protein 2 homolog a [Plasmodium falciparum]	38.58	8.84	3.90
p-cluster293767	VFID 26643	reticulocyte binding protein 2 homolog a [Plasmodium falciparum]	31.48	39.75	4.86
p-cluster249718	VFID 26643	reticulocyte binding protein 2 homolog a [Plasmodium falciparum]	25.61	25.31	2.62
p-cluster350032	VFID 26643	reticulocyte binding protein 2 homolog a [Plasmodium falciparum]	24.44	35.57	5.34
p-cluster245167	VFID 26643	reticulocyte binding protein 2 homolog a [Plasmodium falciparum]	21.67	59.73	5.34
p-cluster233915	VFID 27170	cathepsin B-like cysteine protease [Trypanosoma brucei]	32.98	30.96	77.94
p-cluster321529	VFID 27172	acidocalcisomal pyrophosphatase [Trypanosoma brucei]	39.44	23.06	81.64
p-cluster252457	VFID 27247	trans-sialidase [Trypanosoma cruzi]	27.03	30.18	23.65

p-cluster280772	VFID 27786	trans-sialidase [Trypanosoma cruzi]	38.33	48.17	23.09
p-cluster361406	VFID 27998	small GTPase Rab7A [Entamoeba histolytica]	31.93	37.18	56.80
p-cluster319705	VFID 27999	EhRab7B protein [Entamoeba histolytica]	34.66	16.72	85.02
p-cluster163192	VFID 28596	Mitogen-activated protein kinase 13	43.86	13.73	15.62
p-cluster233538	VFID 28596	Mitogen-activated protein kinase 13	30.96	19.70	90.96
p-cluster321398	VFID 30479	Outer membrane protein yopM	27.76	78.27	66.50
p-cluster331544	VFID 30732	Calglandulin	30.22	22.17	83.97
p-cluster358351	VFID 6246	CMP6L	22.17	12.83	84.81
p-cluster338261	VFID 6873	AMV167	86.84	6.67	93.83
p-cluster328123	VFID 7458	putative chaperone DnAJ [Bacteroides thetaiotaomicron]	50.94	14.64	16.51
p-cluster239806	VFID 7458	putative chaperone DnAJ [Bacteroides thetaiotaomicron]	33.78	14.66	22.43
p-cluster278911	VFID 7841	filamentous hemagglutinin/adhesin [Bordetella pertussis]	29.89	44.71	4.74
p-cluster346852	VFID 7841	filamentous hemagglutinin/adhesin [Bordetella pertussis]	27.44	40.45	4.57
p-cluster288943	VFID 7841	filamentous hemagglutinin/adhesin [Bordetella pertussis]	26.24	68.52	5.65
p-cluster357293	VFID 7922	polysaccharide biosynthesis protein CpsM V [Streptococcus agalactiae]	21.66	38.36	83.82
